# Experimental and computational investigations of RNA duplexes containing N7-regioisomers of adenosine and LNA-adenosine

**DOI:** 10.1093/nar/gkae1222

**Published:** 2024-12-23

**Authors:** Ilyas Yildirim, Witold Andralojc, Amirhossein Taghavi, Daniel Baranowski, Zofia Gdaniec, Ryszard Kierzek, Elzbieta Kierzek

**Affiliations:** Department of Chemistry and Biochemistry, Florida Atlantic University, 5353 Parkside Drive, Jupiter, FL 33458, USA; Institute of Bioorganic Chemistry, Polish Academy of Sciences, Noskowskiego 12/14, 61-704 Poznan, Poland; Department of Chemistry and Biochemistry, Florida Atlantic University, 5353 Parkside Drive, Jupiter, FL 33458, USA; Department of Chemistry, The Scripps Research Institute, 130 Scripps Way, Jupiter, FL 33458, USA; Institute of Bioorganic Chemistry, Polish Academy of Sciences, Noskowskiego 12/14, 61-704 Poznan, Poland; Institute of Bioorganic Chemistry, Polish Academy of Sciences, Noskowskiego 12/14, 61-704 Poznan, Poland; Institute of Bioorganic Chemistry, Polish Academy of Sciences, Noskowskiego 12/14, 61-704 Poznan, Poland; Institute of Bioorganic Chemistry, Polish Academy of Sciences, Noskowskiego 12/14, 61-704 Poznan, Poland

## Abstract

Although glycosidic bonds in purines typically involve the N9 position, the chemical synthesis of adenosine produces N7-ribofuranosyladenine (7A) as a kinetically favorable ribosylation product. Similarly, in the synthesis of LNA-adenosine (A^L^), a minor product, N7-LNA-adenosine (7A^L^), is observed. While extensive research has focused on investigating the properties of N9-regioisomers of adenosine, 7A has been largely overlooked and considered as a side-product. In this study, we conducted comprehensive experimental and computational investigations to elucidate the structural and thermodynamic properties of 7A and 7A^L^. Our results reveal that 7A and 7A^L^ primarily enhance the thermodynamic stability of 1 × 1 mismatches when paired with purines but decrease stability when paired with pyrimidines. Utilizing nuclear magnetic resonance and computational techniques, we discovered that 1 × 1 7A:A and 7A^L^:A prefer *anti*-*anti* conformations, while 1 × 1 7A:G and 7A^L^:G prefer *syn*-*anti* orientations, both forming two hydrogen bond states, resulting in enhanced duplex stabilities. Altogether, these findings underscore the unique properties of 7A and 7A^L^ when incorporated in RNA, which could advance structure-based RNA studies and potentially be utilized to modulate binding affinity, selectivity and biostability of RNA molecules.

## Graphical Abstract

**Graphical Abstract:**
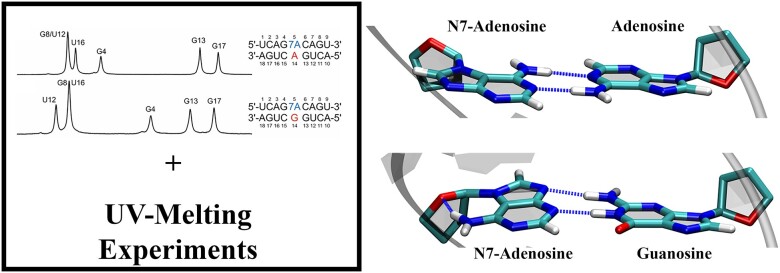

## Introduction

**Figure 1.**
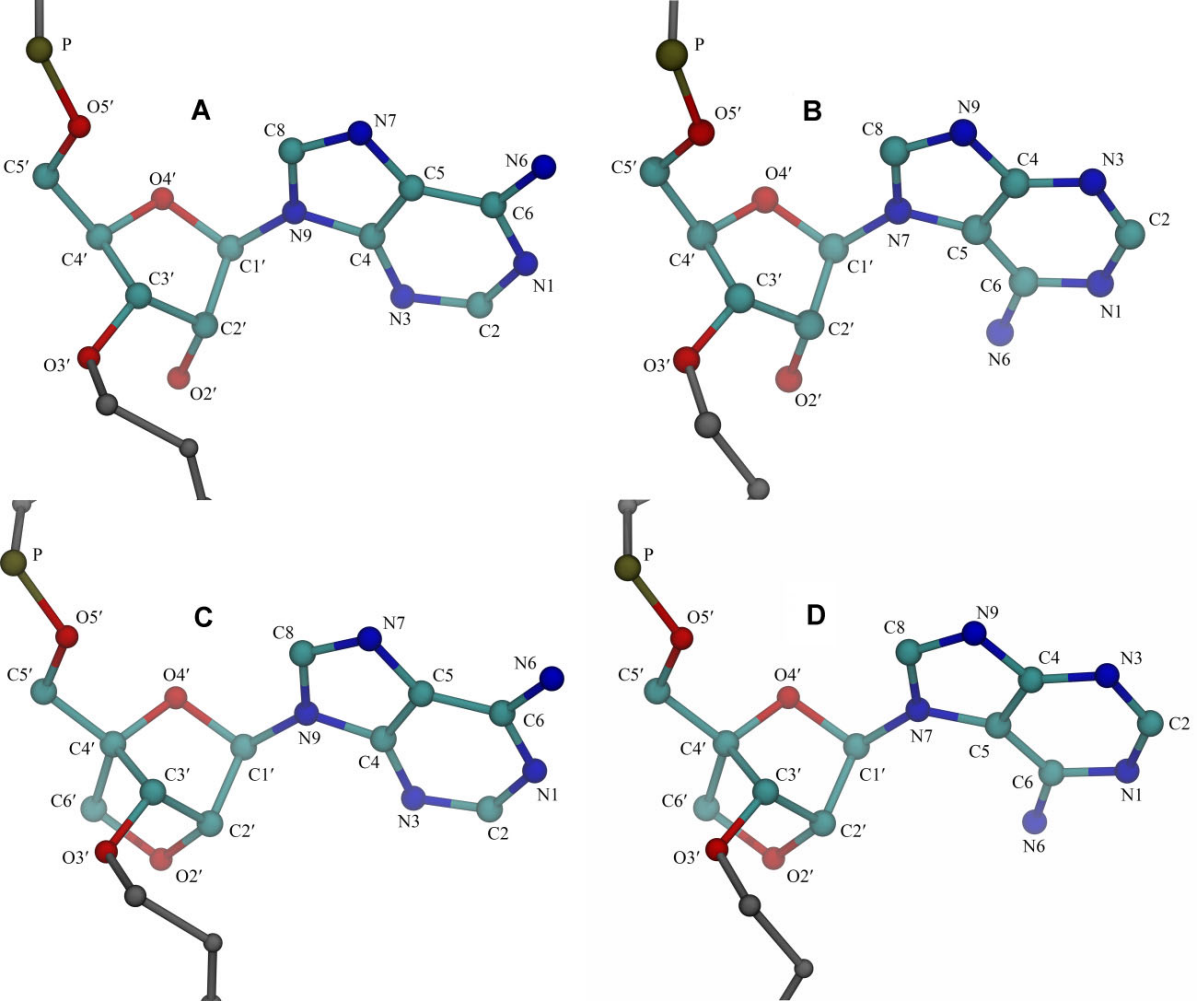
(**A**) N9-ribofuranosyladenine, A, (**B**) N7-ribofuranosyladenine, 7A, (**C**) N9-LNA-adenosine, A^L^ and (**D**) N7-LNA-adenosine, 7A^L^. For simplicity, hydrogen atoms are omitted, and only common atom names are displayed. Note that the O2′−C4′ methylene bridges seen in C and D lock the sugar pucker to C3′-endo.

The glycosidic bonds in purines involve the N9 position. Nevertheless, N7-ribofuranosyladenine (7A) is a modified RNA residue and a kinetically favorable minor product during the chemical synthesis of adenosine (Figure 1A and B) (1). Many efforts have been dedicated to N9-regioisomers of adenosine, while 7A has been largely neglected and considered a side-product. A similar result was observed in the chemical synthesis of LNA-adenosine (A^L^), where a minor product, N7-LNA-adenosine (7A^L^), was formed as well (Figure 1C and D). While limited information is available on 7A and 7A^L^, several reports have investigated the biological activities of N7-guanosine (7G) and its derivatives. For example, substitution of the cytosine with 7G in a triple helix was shown to increase the stability by changing the base-pairing pattern, where 7G mimics the properties of protonated cytosines (2–4). Furthermore, replacing 2′-deoxyguanosine with its 7-regioisomer in several oligonucleotides did not enhance the formation of antiparallel triple helices (5), while acyclic 7G was shown to form DNA triplexes (6). Recently, the base pairing properties of 7-deoxyriboguanine and its 9-deaza analog in duplexes and triplexes were investigated and compared (7,8). Finally, the effects of N7-2′-deoxyadenosine and N7-2′-deoxy-3-deazaguanosine on the duplex stabilities of DNA were studied (9). As mentioned earlier, there is a notable absence of studies focusing on the N7-regioisomers of adenosine, which could elucidate distinctive properties of these systems. Such perspectives have the potential to reveal novel applications in both biophysical and therapeutic research.

Binding affinity, selectivity and biostability of RNA are some of the properties that can be regulated by modified RNA residues in oligonucleotide therapeutics. For example, locked nucleic acid (LNA) residues have been shown to enhance binding affinities and specificities in RNA and DNA (10–13). LNA residues are unnatural nucleotides, where O2′ and C4′ atoms are connected by a methylene bridge that locks the sugar pucker specifically to C3′-endo conformation. This unique property of LNAs affects neighboring residues in RNA and DNA to favor C3′-endo conformations, thereby inducing global structural changes in the duplex (14–17). The rigidification of the sugar pucker and, therefore, the structure is one of the reasons why LNA residues enhance duplex stabilities. The investigation and extraction of such unique properties of modified RNA residues are, thus, critical because they hold the potential to offer novel applications in oligonucleotide-based therapeutics for human diseases.

RNA molecules are inherently flexible which creates unique structural motifs such as internal, hairpin, multibranch and bulge loops to yield unique functional roles to RNA important in cell biology (18,19). Depending on the preferred sugar pucker, base orientation and stacking, RNA residues can adopt a variety of configurations in loop motifs that can affect stability. For example, while C3′-endo sugar pucker is mainly preferred by RNA residues, C2′-endo is another conformation they can adopt. Furthermore, RNA residues can adopt *syn* and *anti* orientations with regards to the glycosidic bond angle, known also as the χ torsional angle, that can dramatically change the conformational stability. The 1 × 1 RNA internal loop motifs are one of the simplest systems one can utilize to understand the properties of single mismatches that have been a focus in multiple studies (20–24). Furthermore, conformational variability adopted by internal loops can be exploited for therapeutic purposes (25–29). Incorporation of modified nucleotides such as A^L^, 7A and 7A^L^ can affect the dynamics of RNA. The presence of A^L^ and 7A^L^ modifications makes the RNA resistant to nuclease degradation at the 3′-O-phosphodiester bonds. Incorporating 7A in oligonucleotides should not inhibit nuclease degradation. Thus, the discovery of the unique properties of these modified RNA residues can have potential uses in the design principles of oligonucleotides targeting RNA in the cell.

In this contribution, we report a comprehensive study on the thermodynamic stabilities of RNA duplexes incorporating 7A, A^L^ and 7A^L^ at select positions using both experimental and computational methods. Ultraviolet (UV) melting experiments were performed on RNA duplexes containing the 1 × 1 *X*:*Y* mismatches (*X* = A, A^L^, 7A and 7A^L^; *Y* = A, C, G and U) to measure duplex hybridization free energies. Extensive structural studies using molecular dynamics (MD) calculations supported by nuclear magnetic resonance (NMR) experiments were performed to explain the structural and thermodynamic properties of RNA duplexes carrying 7A, A^L^ and 7A^L^ residues. Thermodynamic data verified by molecular mechanics calculations display that 7A and 7A^L^ destabilize RNA duplexes when paired with U and C and stabilize RNA duplexes when paired with A and G except 1 × 1 7A:G. MD calculations, combined with NMR data, suggest that both the 1 × 1 7A:G and 7A^L^:G mismatches adopt *syn*-*anti* orientations, with the former being more dynamic compared with the latter. These orientations give rise to states characterized by two hydrogen bonds and intramolecular salt-bridge-like interactions. Remarkably, these structural features are akin to those observed in 1 × 1 G:G internal loop motifs. Furthermore, we determined that 1 × 1 7A:A and 1 × 1 7A^L^:A both adopt *anti*-*anti* orientations and form two hydrogen bond states, creating more stable duplexes than 1 × 1 A:A, which has a single hydrogen bond. We propose that properties of 7A and 7A^L^ can be utilized in stabilizing RNA mismatches involving purines in structure-based RNA studies, thereby potentially yield novel therapeutic strategies targeting RNA-associated diseases.

## Materials and methods

### Synthesis of protected N7-regioisomer of adenosine and its phosphoramidite

N7-regioisomer of adenosine (7A) was synthesized according to published procedure with some modifications (30). Adenine was suspended in hexamethyldisilazane (HMDS; 4 ml/1 mmol adenine) and 10 mg of ammonium sulfate per 1 mmol of adenine was added and refluxed over 16 h. Reaction mixture was then evaporated, dissolved in anhydrous acetonitrile (4 ml/1 mmol scale of synthesis), combined with ß-D-ribofuranose 1,2,3, 5-tetraacetate (1.5 equivalent) and trimethylsilyl trifluoromethanesulfonate (1.5 equivalent) and finally stirred at room temperature for 80 min. After performing thin layer chromatography (TLC) analysis, a saturated aqueous solution of sodium bicarbonate was added to the reaction mixture, which was then extracted three times with dichloromethane. The combined organic layers were dried over anhydrous sodium sulfate, filtered and evaporated. Silica gel column chromatography purification was performed, and acetylated N7-adenosine was isolated with a yield of ∼60%. The structure of 7A was confirmed through 1D and 2D ^1^H and ^13^C NMR experiments (see ‘NMR spectra of 7A and 7AL’ section in Supplementary material). Remaining steps of the synthesis of 5′, 2′ and N6-protected N7-adenosine are described in Supplementary material (see ‘Synthesis of protected N7-regioisomer of adenosine and its phosphoramidite - Extended’ section in Supplementary material). Protected 3′-O-phosphoramidite of 7A was prepared according to published procedure using 2-cyanoethyl N,N,N′,N′-tetraisopropylphosphorodiamidite and equivalent amount of tetrazole (31). After silica gel column chromatography, the yield of the phosphoramidite was ∼80%.

### Synthesis of protected N7-regioisomer of LNA-adenosine and its phosphoramidite

The N7-regioisomer of LNA-adenosine (7A^L^) was a minor product with a yield of ∼20% during the synthesis of N9-LNA-adenosine (A^L^). The condensation reaction utilized 4-C-methanesulfonyloxymethyl-1,2-di-O-acetyl-3,5-di-O-benzyl-D-ribofuranose and HMDS-silylated adenine in the presence of trimethylsilyl triflate in anhydrous acetonitrile. The remaining seven steps of the reaction leading to 5′-O-dimethoxytrityl-N6-isobutyryl-LNA-adenosine were similar to the procedure described by Koshkin *et al.* (32). During silica gel column chromatography, the protected derivative of N7-LNA-A was isolated with a yield of ∼20%. The structure of 7A^L^ was confirmed through 1D and 2D ^1^H and ^13^C NMR experiments (see ‘NMR spectra of 7A and 7A^L^’ section in Supplementary material). Protected 3′-O-phosphoramidite of 7A^L^ was prepared according to published procedure using 2-cyanoethyl N,N,N′,N′-tetraisopropylphosphorodiamidite and equivalent amount of tetrazole (31,33). After silica gel column chromatography, the yield of the phosphoramidite was ∼80%.

### Oligonucleotide synthesis

Oligonucleotides were synthesized on a BioAutomation MerMade12 DNA/RNA synthesizer using β-cyanoethyl phosphoramidite chemistry and commercially available phosphoramidites (ChemGenes, GenePharma), where standard protocols were followed as described previously (33,34). For deprotection, oligoribonucleotides were treated with a mixture of 30% aqueous ammonia and ethanol (3:1 v/v) for 16 h at 55°C. Silyl protecting groups were removed with the use of triethylamine trihydrofluoride. The deprotected oligonucleotides were purified using silica gel TLC in a mixture of 1-propanol, aqueous ammonia and water (55:35:10 v/v/v), as described previously (35). Mass spectrometry analyses (Matrix-Assisted Laser Desorption/Ionization [MALDI]) were performed for most of the oligonucleotides (Supplementary Table S1).

### UV-melting experiments

Thermodynamic measurements were conducted for each RNA duplex at nine concentrations ranging from 1 to 100 μM. Initially, three samples were prepared at high duplex concentrations, and melting analysis was performed, designated as ‘Run 1’. After completing ‘Run 1’, the same three samples were recovered, diluted with buffer and subjected to a second analysis, referred to as ‘Run 2’. Note that the samples in ‘Run 2’ had lower duplex concentrations than those in ‘Run 1’. Following ‘Run 2’, the samples were further diluted and used for a third analysis, designated as ‘Run 3’. All measurements were carried out in a buffer containing 1 M sodium chloride, 20 mM sodium cacodylate and 0.5 mM Na_2_EDTA at pH 7. The concentrations of single-strand oligonucleotides were calculated using the absorbance at 80°C, and the extinction coefficients of the single strands were approximated using a nearest-neighbor model. Absorbance versus temperature melting curves were measured at 260 nm with a heating rate of 1°C min^−1^ from 0 to 90°C on JASCO V-650 UV/Vis spectrophotometer with a thermoprogrammer. The melting curves were analyzed, and the thermodynamic parameters were calculated from a two-state model using MeltWin 3.5 (36). For most duplexes, the Δ*H*° values derived from the *T*_M_^−1^ versus ln(*C*_T_/4) plots were within 15% of those derived from averaging the fits to individual melting curves, as expected from the two-state model. Thus, the thermodynamic data obtained from *T*_M_^−1^ versus log *C*_T_ plots, which are more consistent and reliable, are utilized while discussing the thermodynamic properties of RNA duplexes. It is important to note that the MeltWin allows some flexibility in analyzing UV-melting measurements, which can lead to small variations in the measured thermodynamic parameters. Factors such as how the truncation of melting curves is handled, or which curves are excluded from the final analysis can influence the results. These variations may alter the thermodynamic parameters by 3–5% (see further discussions below).

### NMR spectroscopy of RNA duplexes

For the acquisition of the NMR data, all RNA duplexes were dissolved in a 10 mM sodium phosphate buffer (pH 6.8) containing 150 mM sodium chloride and 0.1 mM EDTA. In order to ensure that RNA is present uniquely in the duplex form, the samples were further washed with the same buffer on an Amicon centrifugal filter with 3-kDa molecular weight cutoff, where any excess single-stranded RNA would pass through the pores of the Amicon membrane. NMR data were collected on a Bruker AVANCE III 700 MHz spectrometer equipped with a QCI CryoProbe. The resonance assignment of non-exchangeable aromatic and anomeric protons was achieved using standard procedures through the analysis of NOESY spectra recorded in 100% D_2_O at 25°C and 35°C (37). In the case of duplexes incorporating 7A^L^:A and A^L^:A mismatches, the assignment of the H2 protons of the two adenine bases in the mismatch was confirmed using an heteronuclear multiple bond correlation (HMBC) type experiment. The H2 and H8 protons of most adenine residues were correlated to each other through cross-peaks to the same C4 carbon frequency. Unfortunately, for 7A^L^ the H8 resonance was slightly broadened and did not yield HMBC correlations. The H2 proton of this residue was thus assigned based on its atypical C4 chemical shift of 161.2 ppm consistent with monomeric 7A^L^ (Supplementary Table S2). All the H2 protons of ‘standard’ adenosine residues in the same duplex correlated to C4 carbon atoms resonating between 148.8 and 150.0 ppm, which is a typical range for A-C4 carbons. No attempt was made to assign the rest of the ribose protons. The exchangeable protons were assigned using NOESY spectra measured in 90% H_2_O/10% D_2_O at 5°C and 25°C. All spectra were analyzed in NMRFAM-Sparky (38).

### Parameterization of 7A and 7A^L^

Nucleoside versions of 7A and 7A^L^ were first created with the LEAP module of AMBER 18 (39). Each molecule was first optimized and then electrostatic potentials at a set of grid points were calculated using Hartree–Fock theory in combination with the 6–31G(d) basis set [HF/6–31G(d)] using Gaussian 09 (40). Restrained electrostatic potential (RESP) charges were derived following the RESP protocol (41). During the charge calculations of 7A and 7A^L^, RESP charges of all except the C1′, H1′ and the base atoms were set to be the same as in adenosine and LNA-adenosine, respectively (Supplementary Tables S3 and S4). Furthermore, χ and α/γ torsional parameters of adenosine and LNA-adenosine were used in 7A and 7A^L^, respectively (14,42,43).

### Preparation of the model systems for MD studies

Twelve RNA systems were created to investigate the properties of 7A and 7A^L^ (Supplementary Table S5). Simulations were carried out with the AMBER 18 (39) simulation package using the PARM99 force field (44) with revised χ (45) and α/γ (43) torsional parameters. The nucleic acid builder module of AMBER 18 (39) was utilized to build the initial structures for the unmodified systems of A-U, A-C, A-A and A-G in A-form RNA orientations, which then were used to homology model 7A-U, 7A-C, 7A-A, 7A-G, 7A^L^-A and 7A^L^-G (see Supplementary Table S5 for details). To determine the preferred conformational state for each system, four initial structures were created for each system, where the initial structures of the middle base pairs were designed to be in *syn*-*syn*, *anti*-*syn*, *syn*-*anti* and *anti*-*anti* orientations, which allowed us to scan a wider conformational space while determining the global minimum structures. For this purpose, torsional restraints on χ were imposed on these residues to create the initial states in *syn* and *anti* orientations, which were then used in explicit solvent MD simulations without any restraints. Each system was first neutralized with Na^+^ ions (46), which then was solvated with 9000 TIP3P (47) water molecules in a truncated octahedral box using the LEAP module of AMBER 18 (39). Each system was then added with extra 13 Na^+^ and Cl^−^ ions to mimic physiological conditions, where after equilibration each system had 0.17 M Na^+^ concentrations.

### MD simulations

The structures were minimized in two steps, where positional restraints with restraint weights of 1 kcal mol^−1^ Å^−2^ were applied only in the first step while no restraints were imposed in the second step. Each minimization step included 5000 steps of steepest descent minimization subsequently followed by 5000 steps of conjugate-gradient minimization. Minimization was followed by an equilibration protocol first in constant volume with positional restraints imposed on all the atoms of RNA with a restraint weight of 1 kcal mol^−1^ Å^−2^. During the first step of equilibration, temperature was increased from 0 to 300 K in 2 ns using the Langevin thermostat with a 1 ps^−1^ collision frequency at constant volume. Another 2 ns of MD run was followed in the second step of equilibration using constant pressure MD with temperature at 300 K, and pressure at 1 bar with pressure relaxation time of 1 ps. No restraints were used in explicit solvent MD simulations. After minimization and equilibration, a 4 μs MD simulation with a 2 ps time step was performed on each system at constant pressure MD with isotropic position scaling. In 7A-A and 7A-G, which were started in *syn*-*syn* orientations, MD simulations were extended to cover over 6 and 10 μs, respectively (Supplementary Table S6). SHAKE (48) was turned on for constraining bonds involving hydrogen atoms. An atom-based long-range cutoff of 10.0 Å was used in all the minimization and MD runs. The Particle Mesh Ewald method was used to handle the electrostatics (49). Simulations were performed using pmemd.MPI, pmemd.cuda and pmemd.cuda.MPI of AMBER 18.

### Computational analyses

Dihedral, hydrogen-bond and root-mean-square deviation (RMSD) analyses were completed using the cpptraj module of AMBER 18 (39). Hydrogen-bond analyses were done only on the middle base pairs to see if formation or loss of hydrogen bonds are correlated with the experimental data (Supplementary Script S1). An in-house code was utilized to perform cluster analyses as described before (50–60) (Supplementary Script S2). For each system, all four MD trajectories (*anti*-*anti*, *syn*-*anti*, *anti*-*syn* and *syn*-*syn*) were first combined. Snapshots with RMSD ≤0.5 Å of the middle base pair were clustered into the same group. Average structures of each cluster were calculated at the end using all the structures in that cluster. In order to calculate the hybridization free energies, we utilized the molecular mechanics/three-dimensional reference interaction site model (MM/3D-RISM) and normal mode (NMODE) approaches of AMBER 18, where both of the terminal two base pairs were removed prior to calculations (Supplementary Script S3). The reference interaction site model of molecular solvation provides the 3D map of density distribution of the solvent around the solute (61,62). The probability density *ρ*_γ_*g*_γ_(*r*) of finding the interaction sites (γ) of solvent molecules positioned around the solute, in the 3D space (*r*) is used to represent the solvent structure. The Kovalenko–Hirata closure (3D-RISM-KH) (63) molecular solvation theory was utilized in the calculations.

## Results

### Determination of the structure of 7A and 7A^L^

The structures of 7A and 7A^L^ nucleosides were verified by a series of various NMR experiments. The diagnostic signals that unequivocally confirmed the substitution at N7 position of adenine with D-ribose were heteronuclear long-range correlations C5-H1′ and C8-H1′ observed on ^1^H-^13^C gHMBC spectra (see ‘NMR spectra of 7A and 7A^L^’ section in Supplementary material, and Supplementary Figures S1–S14). Further evidence supporting N7 substitution is provided by a comparative analysis of the ^1^H and ^13^C chemical shifts of compounds A and 7A (see ‘NMR spectra of 7A and 7A^L^’ section in Supplementary material, and Supplementary Tables S2 and S7). Additionally, orientation around glycosidic bond of 7A and 7A^L^ preferred *anti* or *high-anti* orientation and conformation of β-D-ribofuranose moiety was determined as C1′-exo and C3′-endo, respectively (Supplementary Table S8). These N7-derivatives were used to synthesize the 3′-O-phosphoramidites and respective N7-modified RNA oligonucleotides for thermodynamic UV-melting studies.

### Thermodynamic properties of RNA duplexes with 7A and 7A^L^

Natural RNA and DNA nucleotides contain glycosidic bonds between C1′ of ribose and N9/N1 of purine/pyrimidine. The exception is pseudouridine (Ψ) and its derivatives, which display glycosidic bonds between C1′ and C5 atoms. Herein, we investigate the properties of 7A and 7A^L^ modified RNA oligonucleotides. Inclusion of N7-regioisomer analogues of adenosine in oligonucleotides dramatically changes base paring and thermodynamic properties. In order to perform the thermodynamic studies, we utilized RNA duplexes, such as 5′UCAG***X***CAGU/3′AGUC***Y***GUCA, where *X* was A, 7A, A^L^ and 7A^L^ and *Y* was A, C, G and U (Table 1). As a result, model duplexes were forming either canonical base pairs in the middle of the duplexes, such as A:U and A^L^:U, or 1 × 1 non-canonical base pairs.

**Table 1.** Thermodynamic parameters of RNA duplexes containing adenosine (A), N7-adenosine (7A), LNA-adenosine (A^L^) and N7-LNA-adenosine (7A^L^) positioned centrally within the duplex^a^

		Average of curve fits				*T*_M_^−1^ versus log *C*_T_ plots				Comparison	
Duplexes (5′-3′)	Short notation	−Δ*H*° (kcal mol^−1^)	−Δ*S*° (eu)	−Δ*G*°_37_ (kcal mol^−1^)	*T*_M_^b^ (°C)	−Δ*H*° (kcal mol^−1^)	−Δ*S*° (eu)	−Δ*G*°_37_ (kcal mol^−1^)	*T*_M_^b^ (°C)	ΔΔ*G*°_37_^c^ (kcal mol^−1^)	Δ*T*_M_^d^ (°C)
5′ UCAG **A** CAGU 3′ AGUC **U** GUCA	A-U	78.2 ± 1.3	213.2 ± 3.8	12.04 ± 0.11	60.5	75.8 ± 2.7	204.1 ± 8.1	11.86 ± 0.17	60.7	0.00	0.0
5′ UCAG **7A** CAGU 3′ AGUC **U** GUCA	7A-U	71.1 ± 5.7	204.4 ± 18.6	7.73 ± 0.14	42.3	61.4 ± 3.6	173.2 ± 11.5	7.71 ± 0.06	43.1	4.15 ± 0.18	−17.6
5′ UCAG **A^L^**CAGU 3′ AGUC**U** GUCA	A^L^-U	83.3 ± 2.3	224.5 ± 6.6	13.66 ± 0.32	66.0	72.4 ± 5.5	192.2 ± 16.4	12.81 ± 0.44	66.4	−0.95 ± 0.47	5.7
5′ UCAG **7A^L^**CAGU 3′ AGUC **U** GUCA	7A^L^-U	74.6 ± 2.8	209.8 ± 8.5	9.47 ± 0.26	49.7	80.8 ± 7.6	229.5 ± 23.8	9.66 ± 0.28	49.5	2.20 ± 0.33	−11.2
5′ UCAG **A** CAGU 3′ AGUC **C** GUCA	A-C	78.4 ± 3.9	224.3 ± 12.4	8.87 ± 0.14	46.5	89.7 ± 12.7	260.4 ± 40.7	9.00 ± 0.21	45.8	0.00	0.0
5′ UCAG **7A** CAGU 3′ AGUC **C** GUCA	7A-C	74.0 ± 5.6	216.1 ± 17.9	6.99 ± 0.16	38.9	82.7 ± 14.1	244.4 ± 45.6	6.93 ± 0.38	38.5	2.07 ± 0.43	−7.3
5′ UCAG **A^L^**CAGU 3′ AGUC **C** GUCA	A^L^-C	71.3 ± 0.9	199.2 ± 2.8	9.48 ± 0.11	50.4	62.1 ± 1.0	170.2 ± 3.1	9.27 ± 0.02	51.3	−0.27 ± 0.21	5.5
5′ UCAG **7A^L^**CAGU 3′ AGUC **C** GUCA	7A^L^-C	71.8 ± 2.8	203.5 ± 8.9	8.73 ± 0.12	46.8	67.6 ± 2.1	190.1 ± 6.8	8.61 ± 0.05	46.8	0.39 ± 0.22	1.0
5′ UCAG **A** CAGU 3′ AGUC **A** GUCA	A-A	70.7 ± 5.8	202.6 ± 18.6	7.84 ± 0.16	42.9	68.4 ± 10.2	195.4 ± 32.5	7.80 ± 0.33	42.8	0.00	0.0
5′ UCAG **7A** CAGU 3′ AGUC **A** GUCA	7A-A	72.3 ± 4.1	205.9 ± 12.7	8.49 ± 0.19	45.6	79.1 ± 7.1	227.9 ± 22.6	8.53 ± 0.14	45.0	−0.73 ± 0.36	2.2
5′ UCAG **A^L^**CAGU 3′ AGUC **A** GUCA	A^L^-A	64.2 ± 3.8	180.5 ± 11.7	8.23 ± 0.18	45.4	58.5 ± 4.1	162.5 ± 13.0	8.13 ± 0.10	45.7	−0.33 ± 0.34	2.9
5′ UCAG **7A^L^**CAGU 3′ AGUC **A** GUCA	7A^L^-A	73.2 ± 2.0	204.6 ± 5.9	9.80 ± 0.17	51.5	67.6 ± 2.1	187.0 ± 6.7	9.60 ± 0.07	51.8	−1.80 ± 0.34	9.0
5′ UCAG **A** CAGU 3′ AGUC **G** GUCA	A-G	64.9 ± 2.2	197.0 ± 6.8	8.73 ± 0.13	47.1	64.5 ± 3.3	180.3 ± 10.3	8.61 ± 0.08	47.3	0.00	0.0
5′ UCAG **7A** CAGU 3′ AGUC **G** GUCA	7A-G	59.4 ± 1.5	166.8 ± 4.0	7.62 ± 0.06	42.8	60.0 ± 4.3	168.8 ± 13.9	7.62 ± 0.08	42.8	0.99 ± 0.11	−4.5
5′ UCAG **A^L^**CAGU 3′ AGUC **G** GUCA	A^L^-G	69.1 ± 2.8	192.1 ± 9.0	9.49 ± 0.02	50.9	61.1 ± 1.0	167.0 ± 3.2	9.32 ± 0.02	51.8	−0.71 ± 0.08	4.5
5′ UCAG **7A^L^**CAGU 3′ AGUC **G** GUCA	7A^L^-G	74.8 ± 2.4	208.9 ± 7.7	9.98 ± 0.08	52.0	71.8 ± 2.6	199.8 ± 8.3	9.89 ± 0.08	52.2	−1.28 ± 0.11	4.90

^a^Solution: 1 M sodium chloride, 20 mM sodium cacodylate, 0.5 mM Na_2_EDTA, pH 7.

^b^Calculated for 10^−4^ M oligomer concentration (7A = N7-regioisomer of A; 7A^L^ = N7-regioisomer of LNA-A; and A^L^ = LNA-A).

^c^Comparison of free energies ($\Delta G_{37}^{\circ}$) to A-*X* (*X* = A, C, G and U), where $\Delta \Delta G_{37,Y - X}^{\circ} = \Delta G_{37,Y - X}^{\circ} - \Delta G_{37,A - X}^{\circ}$ (*Y* = A, 7A, A^L^ and 7A^L^). The standard deviation (SD) of $\Delta \Delta G_{37,Y - X}^{\circ}$ is calculated as $SD( {\Delta \Delta G_{37,Y - X}^{\circ}}) = \sqrt {{{({SD( {\Delta G_{37,Y - X}^{\circ}} )} )}^2} + {{( {SD( {\Delta G_{37,A - X}^{\circ}} )} )}^2}}$ because $\Delta G_{37,Y - X}^{\circ}$ and $\Delta G_{37,A - X}^{\circ}$ are independent.

^d^Comparison of melting temperatures to A-*X* (where *X* = A, C, G and U), respectively. Residues in bold indicate the 1 × 1 internal loop mismatch pairs.

### Contribution of 7A to the thermodynamic stabilities of RNA duplexes

The major difference between A and 7A is related to the glycoside bond linking ribose to adenine. Inversion of adenine moiety with respect to sugar changes the locations of functional groups involved in base pairing with the complementary strand. To evaluate the effects of 7A, thermodynamic stabilities of different RNA duplexes were measured using UV-melting method (Table 1). Duplex hybridization energies, Δ*G*°_37_, were measured to be −7.71, −6.93, −8.53 and −7.62 kcal mol^−1^ in 7A-U, 7A-C, 7A-A and 7A-G, respectively, whereas Δ*G*°_37_ of A-U, A-C, A-A and A-G were measured to be −11.86, −9.00, −7.80 and −8.61 kcal mol^−1^, respectively, suggesting that A and 7A possess distinct structural properties when paired with pyrimidines and purines, consequently altering their thermodynamic characteristics in comparison to N9 counterparts. The substitution of A with 7A reduces thermodynamic stability by 4.15, 2.07 and 0.99 kcal mol^−1^ when paired with U, C and G, respectively (Table 1). In contrast, a 0.73 kcal mol^−1^ increase in thermodynamic stability was observed in 7A-A compared with the A-A (Table 1).

### Contribution of 7A^L^ to thermodynamic stabilities of RNA duplexes

Compared with natural RNA residues, LNAs enhance thermodynamic stabilities of RNA helices by 1.5–1.7 kcal mol^−1^ per each LNA residue (34,64). It was previously reported that improved stacking and hydrogen bonding patterns as well as structural preorganization, which is due to exclusive preference of C3′-endo sugar pucker in LNA residues, were responsible for the enhancements (14,64). Using UV-melting experiments, duplex hybridization energies for 7A^L^-U, 7A^L^-C, 7A^L^-A and 7A^L^-G were measured to be −9.66, −8.61, −9.60 and −9.89 kcal mol^−1^, respectively, while the corresponding energies for A^L^-U, A^L^-C, A^L^-A and A^L^-G were measured to be −12.81, −9.27, −8.13 and −9.32 kcal mol^−1^, respectively (Table 1). Substitution of A^L^ with 7A^L^ indicates that 1 × 1 7A^L^:U and 1 × 1 7A^L^:C mismatches destabilize the duplexes by 3.15 and 0.66 kcal mol^−1^, respectively, compared with 1 × 1 A^L^:U and 1 × 1 A^L^:C mismatches. In contrast 1 × 1 7A^L^:A and 1 × 1 7A^L^:G mismatches stabilize the duplexes by 1.47 and 0.57 kcal mol^−1^, respectively, compared with 1 × 1 A^L^:A and 1 × 1 A^L^:G, respectively. These results are similar to the outcomes observed on substitution of A with 7A as described above. When paired with pyrimidines, both 7A and 7A^L^ destabilize the duplexes, whereas when paired with purines, they stabilize them except 7A-G (Table 1).

### Conformational preferences of 7A and 7A^L^ according to NMR

**Figure 2.**
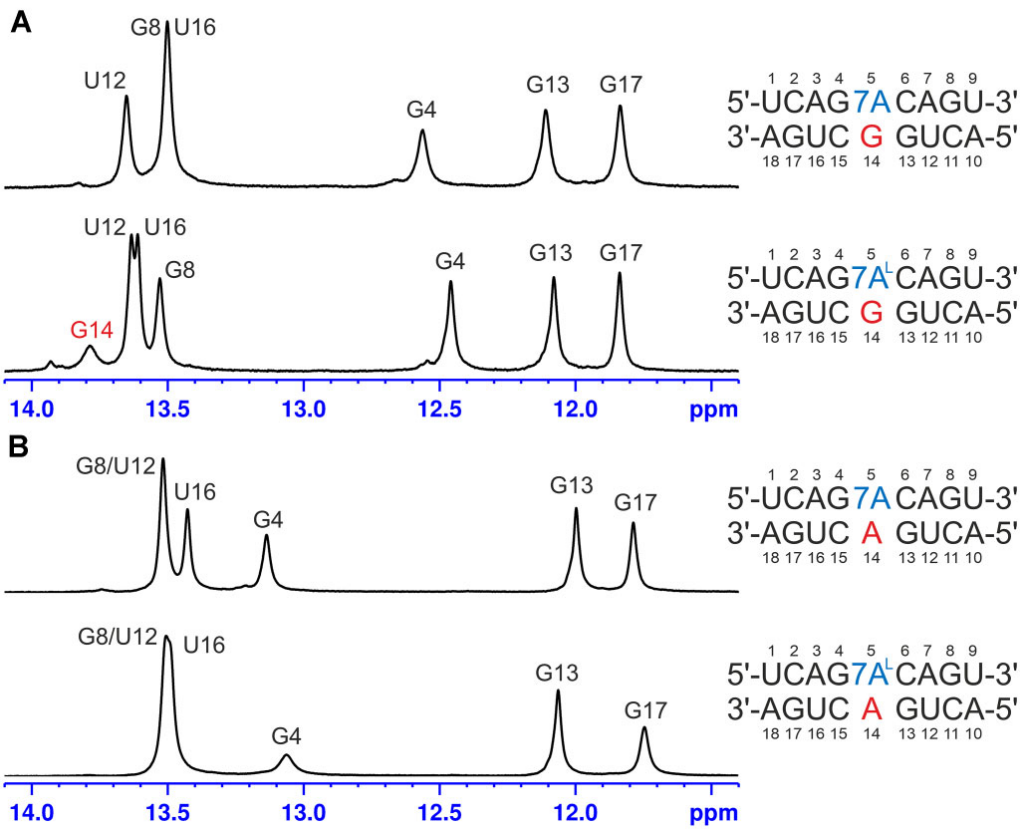
The imino proton regions of the 1D ^1^H NMR spectra of duplexes containing (**A**) 1 × 1 7A:G and 1 × 1 7A^L^:G mismatches recorded at 20°C and (**B**) 1 × 1 7A:A and 1 × 1 7A^L^:A mismatches recorded at 25°C.

Our thermodynamic results show that 7A stabilizes the duplex when paired with A relative to A-A but destabilizes it when paired with G compared to A-G (Table 1). In contrast, 7A^L^ stabilizes duplexes when paired with both G and A relative to A-A and A-G (Table1). We thus decided to investigate the structural basis for such increased stabilities using NMR spectroscopy. Four duplexes containing the 7A-G, 7A^L^-G, 7A-A and 7A^L^-A pairings (Table 1) were obtained in NMR-compatible quantities and studied using 1D (Figure 2) and 2D NMR experiments in order to provide hints regarding the mutual arrangement of the two interacting bases. The NMR observables used for this purpose included (i) the intensities of intraresidue H8/H6-H1′ NOE cross-peaks reporting the states of glycosidic bonds of the bases (*syn/anti*) (Table 2), (ii) the identities of exchangeable protons observed in the central mismatch to identify groups likely involved in hydrogen bonding interactions (*vide infra*) and (iii) a list of NOEs and their intensities, which are structurally significant and involve base protons of the central mismatch (Supplementary Table S9).

**Table 2.** Comparison of experimental results with computational predictions (for details see Tables 1 and Supplementary Tables S12–S23)

		Experimental		Computational^a^			
Short notation	Duplexes (5′-3′)	ΔΔ*G*°_37_^b^	Loop orientation^c^	Δ*G*_pred_^d^	ΔΔ*G*_pred_^e^	Loop orientation	Hydrogen bonds^f^
A-U	5′ UCAG **A** CAGU 3′ AGUC **U** GUCA	0.00	*anti*-*anti*	1.86	0.00	*anti*-*anti*	1.98
7A-U^g^	5′ UCAG **7A** CAGU 3′ AGUC **U** GUCA	4.15		5.43	3.57	*anti*-*anti*	1.16
7A^L^-U	5′ UCAG **7A^L^**CAGU 3′ AGUC **U** GUCA	2.20		2.85	0.99	*syn*-*anti*	1.00
A-C	5′ UCAG **A** CAGU 3′ AGUC **C** GUCA	0.00		4.34	0.00	*anti*-*syn*	1.00
7A-C^h^	5′ UCAG **7A** CAGU 3′ AGUC **C** GUCA	2.07		5.51	1.17	*syn*-*anti*	0.97
7A^L^-C^i^	5′ UCAG **7A^L^**CAGU 3′ AGUC **C** GUCA	0.39		4.76	0.42	*anti*-*anti*	0.99
A-A	5′ UCAG **A** CAGU 3′ AGUC **A** GUCA	0.00	*anti*-*anti*^j^	3.48	0.00	*anti*-*anti*	0.96
7A-A	5′ UCAG **7A** CAGU 3′ AGUC **A** GUCA	−0.73	*anti*-*anti*	1.50	−1.98	*anti*-*anti*	1.99
7A^L^-A	5′ UCAG **7A^L^**CAGU 3′ AGUC **A** GUCA	−1.80	*anti*-*anti*	1.44	−2.04	*anti*-*anti*	1.99
A-G	5′ UCAG **A** CAGU 3′ AGUC **G** GUCA	0.00		2.81	0.00	*syn*-*anti*	1.99
7A-G	5′ UCAG **7A** CAGU 3′ AGUC **G** GUCA	0.99	*syn*-*anti*	2.10	−0.71	*syn*-*anti*	1.54
7A^L^-G	5′ UCAG **7A^L^**CAGU 3′ AGUC **G** GUCA	−1.28	*syn*-*anti*	1.22	−1.59	*syn*-*anti*	1.75

^a^Predicted global minima bound states are displayed (see Supplementary Tables S12–S23 for details).

^b^Experimental ΔΔ*G*°_37_ results in kcal mol^−1^ measured from *T*_M_^−1^ versus log *C*_T_ plots (Table 1).

^c^Loop orientations observed in NMR.

^d^Δ*G*_pred_ = Δ*G*_MM/3D-RISM_ – Δ*G*_NMODE_ (in kcal mol^−1^) (Supplementary Tables S12–S23).

^e^ΔΔ*G*_pred_ is the predicted free energy difference with respect to A-*X*, where *X* = U, C, A and G. For example, ΔΔ*G*_pred,7A-U_ = Δ*G*_pred,7A-U_ − Δ*G*_pred,A-U_.

^f^Average number of hydrogen bonds observed in the middle base pair.

^g^Cluster analyses display that this system has both *anti*-*anti* and *syn*-*anti* orientations with almost similar predicted Δ*G*_pred_ and hydrogen bonds (Supplementary Table S13).

^h^Cluster analyses display that this system has both *syn*-*anti* and *anti*-*anti* orientations with relatively similar predicted Δ*G*_pred_ and hydrogen bonds (Supplementary Table S16).

^i^Cluster analyses display that this system has *anti*-*anti*, *syn*-*syn*, *syn*-*anti* and *anti*-*syn* orientations with relatively similar predicted Δ*G*_pred_ and hydrogen bonds (Supplementary Table S17).

^j^From literature (71,74,75). Residues in bold indicate the 1 × 1 internal loop mismatch pairs.

**Figure 3.**
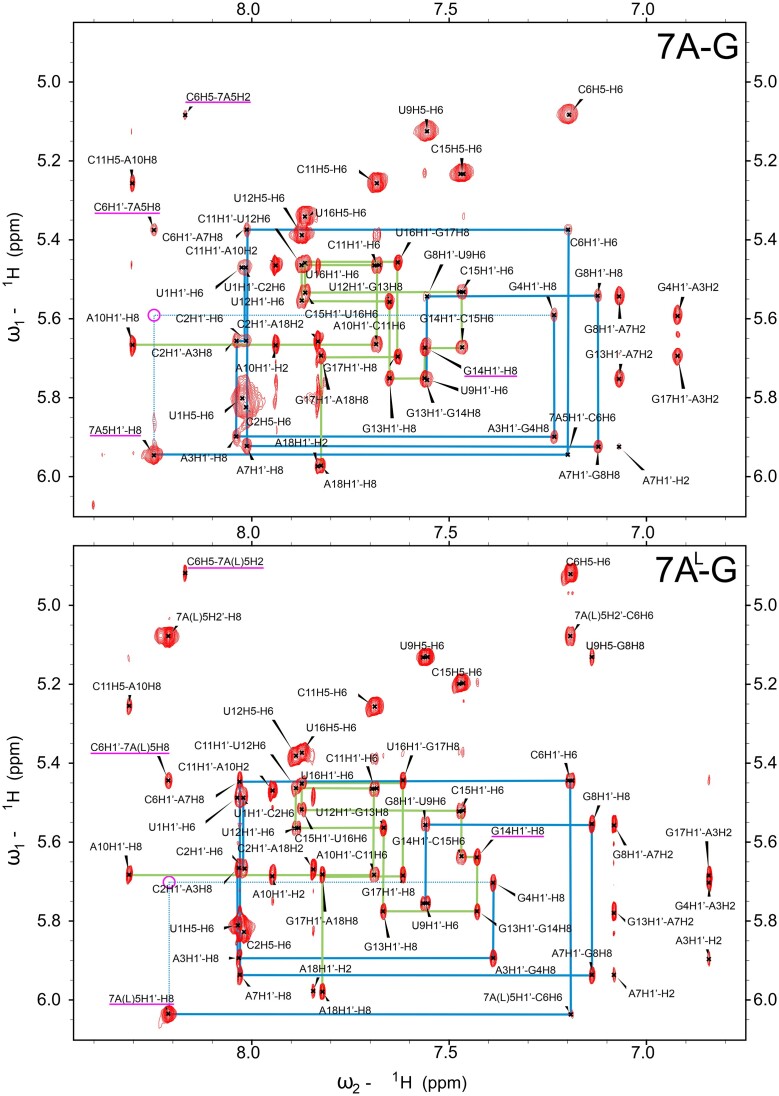
The aromatic-to-anomeric regions of the 2D ^1^H-^1^H NOESY spectra recorded for duplexes containing 1 × 1 7A:G and 1 × 1 7A^L^:G mismatches. Significant NOEs discussed in the text are marked in magenta. Sequential connectivity paths for the two strands are displayed in green and blue lines. For residue numbering see Figure 2.

For the 7A-G and 7A^L^-G containing duplexes the NMR results show that the central 1 × 1 mismatches adopt *syn-anti* conformations, however with 7A-G likely being more dynamic than 7A^L^-G (Table 2). In both systems, the NOE intensities of 7A5H1′-H8 and 7A^L^5H1′-H8 are four times greater than the averages established over the other residues throughout the duplexes (Figures 2 and 3). For the opposing G14 residue, on the other hand, the intensities of G14H1′-H8 in 7A^L^-G and 7A-G are within the said average and slightly above 1 SD from it, respectively (Figures 2 and 3). In addition, the 7A5H8 and 7A^L^5H8 protons do not produce the expected cross-peaks to the preceding G4H1′, but correlate to the following C6H1′ (Figures 2 and 3), which is a characteristic pattern observed in helices having a single nucleotide in *syn* state (65). On the other hand, the exchangeable imino proton of G14, G14H1, is observed in 7A^L^-G but not in 7A-G (Figure 2). At low temperatures, the imino resonance in 7A^L^-G appears to be relatively sharp, but as the temperature increases, it progressively broadens and becomes barely detectable at 35°C (Supplementary Figure S15). Such a level of protection against solvent exchange suggests that this resonance is involved in a relatively stable hydrogen bonding interaction. The -NH_2_ group of the central G14 was unfortunately not observable in the spectra of both systems, which is, however, expected even for -NH_2_ groups of standard Watson–Crick GC pairs (66). As a result, we could not evaluate the hydrogen bonding states of the amino groups of G14. Interestingly, the imino proton of the central G14 exhibits a relatively strong NOE cross-peak to 7A^L^5-H2 proton, but not to 7A^L^5-H8 (Supplementary Figure S16). NOESY spectrum recorded in D_2_O also reveals that the 7A5H2 and 7A^L^5H2 protons give only a single and weak NOE to C6-H5 (Figures 2 and 3).

**Figure 4.**
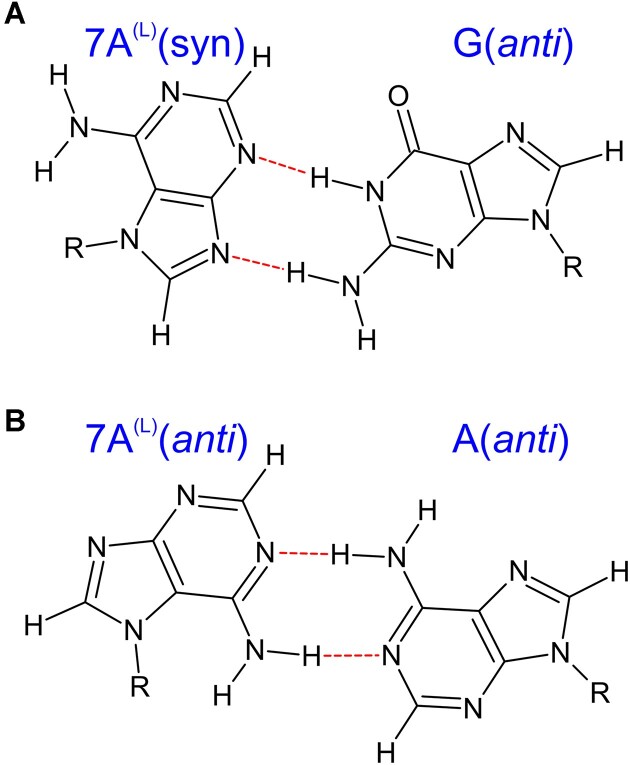
Proposed base pairing geometries for 1 × 1 7A:G and 7A^L^:G (**A**) and 1 × 1 7A:A and 7A^L^:A (**B**) mismatches using NMR data.

Given the fact that the 7A^L^ is in *syn* conformation, which is supported by the NOE patterns mentioned above, the hydrogen bonding partner of the imino proton of G14 in 7A^L^-G is most likely the N3 nitrogen of 7A^L^ (Figure 4). Such a geometry would also bring the -NH_2_ group of G14 close to N9 nitrogen of 7A^L^ suggesting a base pairing stabilized by two hydrogen bonds (Figures 1A, 2 and 4). For the 7A-G system, where the imino proton of G in 1 × 1 7A:G mismatch is not observable, it is more difficult to confidently establish the base pairing geometry. However, given the fact that both 7A^L^-G and 7A-G adopt the same glycosidic states and exhibit very similar aromatic and anomeric chemical shift patterns (Supplementary Table S10) it is reasonable to suggest that both systems display similar pairing geometries, with perhaps the pairing in 7A-G being more labile, precluding the observation of the hydrogen bonded imino proton. This may also explain why replacing adenosine in A-G with 7A^L^ enhances duplex stability, whereas the opposite effect is observed with 7A (Table 1).

**Figure 5.**
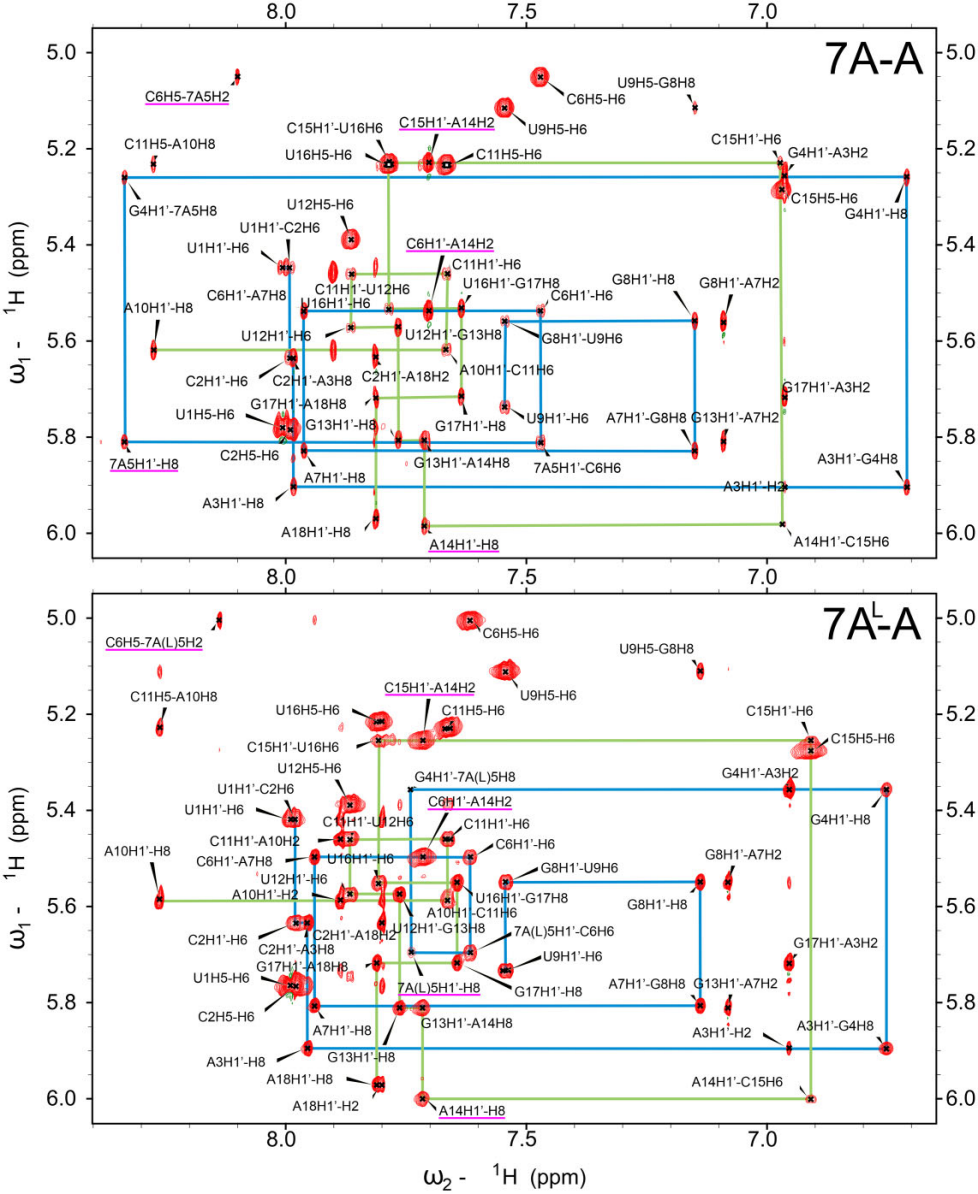
The aromatic-to-anomeric regions of the 2D ^1^H-^1^H NOESY spectra recorded for duplexes containing 1 × 1 7A:A and 1 × 1 7A^L^:A mismatches. Significant NOEs discussed in the text are marked in magenta. Sequential connectivity paths for the two strands are displayed in green and blue lines. For residue numbering see Figure 2.

In duplexes containing 7A-A and 7A^L^-A (Table 2), H1′-H8 NOE cross-peak intensities within the central pairs do not differ significantly from other pairs implying that the 7A-A and 7A^L^-A mismatches adopt the *anti-anti* glycosidic conformations (Figures 2 and 5). Unfortunately, no direct information regarding hydrogen bonding patterns can be established for these systems as the -NH_2_ protons of all adenine bases in question are missing in the NMR spectra. This is usually the case even for adenosine residues forming stable Watson–Crick A-U pairs (66). The H2 protons within the central mismatch exhibit very similar NOE patterns with the neighboring base pairs in both 7A-A and 7A^L^-A systems (Figures 2 and 5). Also, the chemical shifts measured for 7A-A and 7A^L^-A are very similar except for H8 proton, which is expected due to the differences in ribose sugar of RNA and LNA residues (Supplementary Table S11). Similar to the results observed in 7A-G and 7A^L^-G, the findings suggest that the geometries of the central 1 × 1 mismatches in 7A-A and 7A^L^-A are identical. However, for the 7A-A and 7A^L^-A duplexes it is much more difficult to establish the common geometry from the NMR data alone due to the absence of hydrogen bond information. One pairing geometry satisfying the NMR observations is shown in Figure 4B. Finally, the systems of 7A^L^-C and 7A^L^-U were also investigated. However, significant line broadening was observed in the central mismatches, making thorough analysis impossible due to the conformational exchange process occurring in these systems (data not shown).

### Predicted binding free energies are mostly in line with experimental results

As described above, N7-glycosylation of adenine as seen in 7A and 7A^L^ places the functional groups to different regions on adenine compared with natural N9-glycosylated adenine that sets different properties to 7A and 7A^L^ (Figure 1). In order to describe an in-depth understanding of the properties of 7A and 7A^L^, we utilized MD simulations, and performed binding free energy calculations to determine if any structural differences observed in the duplexes containing 7A and 7A^L^ can explain the experimental thermodynamic and NMR data (Tables 1 and 2). Four initial structures were created for each system to scan structurally important conformational space, where the middle base pairs of each duplex were homology modeled to be in *anti*-*anti*, *syn*-*anti*, *anti*-*syn* and *syn*-*syn* orientations (Supplementary Table S5). After cluster and then binding analyses, we found that predicted binding free energies, ΔΔ*G*_pred_, are qualitatively in line with experimental results (Table 2, and Supplementary Tables S12–S23). While destabilizing RNA duplexes when paired with pyrimidines, 7A and 7A^L^ stabilize systems when paired with purines (Table 2). For example, 7A-A, 7A^L^-A, 7A-G and 7A^L^-G are predicted to have lower hybridization energies compared with A-A and A-G, which is in line with experimental thermodynamic data except 7A-G (Tables 1 and 2). As an example, ΔΔ*G*_pred,*X*_, defined as ΔΔ*G*_pred,*X*_ = Δ*G*_pred,*X*,_ − Δ*G*_pred,*Y*_ (*X* = 7A-U, 7A-C, 7A-A, 7A-G, 7A^L^-U, 7A^L^-C, 7A^L^-A and 7A^L^-G and *Y* = A-U, A-C, A-A and A-G), was predicted to be −1.98, −2.04, −0.71 and −1.59 kcal mol^−1^ for 7A-A, 7A^L^-A, 7A-G and 7A^L^-G, respectively, while experimental thermodynamic data measured the corresponding ΔΔ*G*°_37_ values as −0.73, −1.80, 0.99 and −1.28 kcal mol^−1^ (Table 2). Furthermore, computational predictions also display destabilization in 7A-U, 7A^L^-U, 7A-C and 7A^L^-C, which is also observed in thermodynamic data (Tables 1 and 2). The calculated ΔΔ*G*_pred_ values for 7A-U, 7A^L^-U, 7A-C and 7A^L^-C are 3.57, 0.99, 1.17 and 0.42 kcal mol^−1^, respectively, while measured ΔΔ*G*°_37_ values are 4.15, 2.20, 2.07 and 0.39 kcal mol^−1^ (Table 2). Linear regression analysis of the data (predicted versus experimental) shows a correlation coefficient of 0.96. Following the confirmation of a strong correlation between the predictions and experimental outcomes regarding the thermodynamics of the studied sequences, we investigated the structural details extracted from MD calculations.

### When paired with pyrimidines, 7A and 7A^L^ destabilize RNA duplexes due to loss of hydrogen bond and stacking

**Figure 6.**
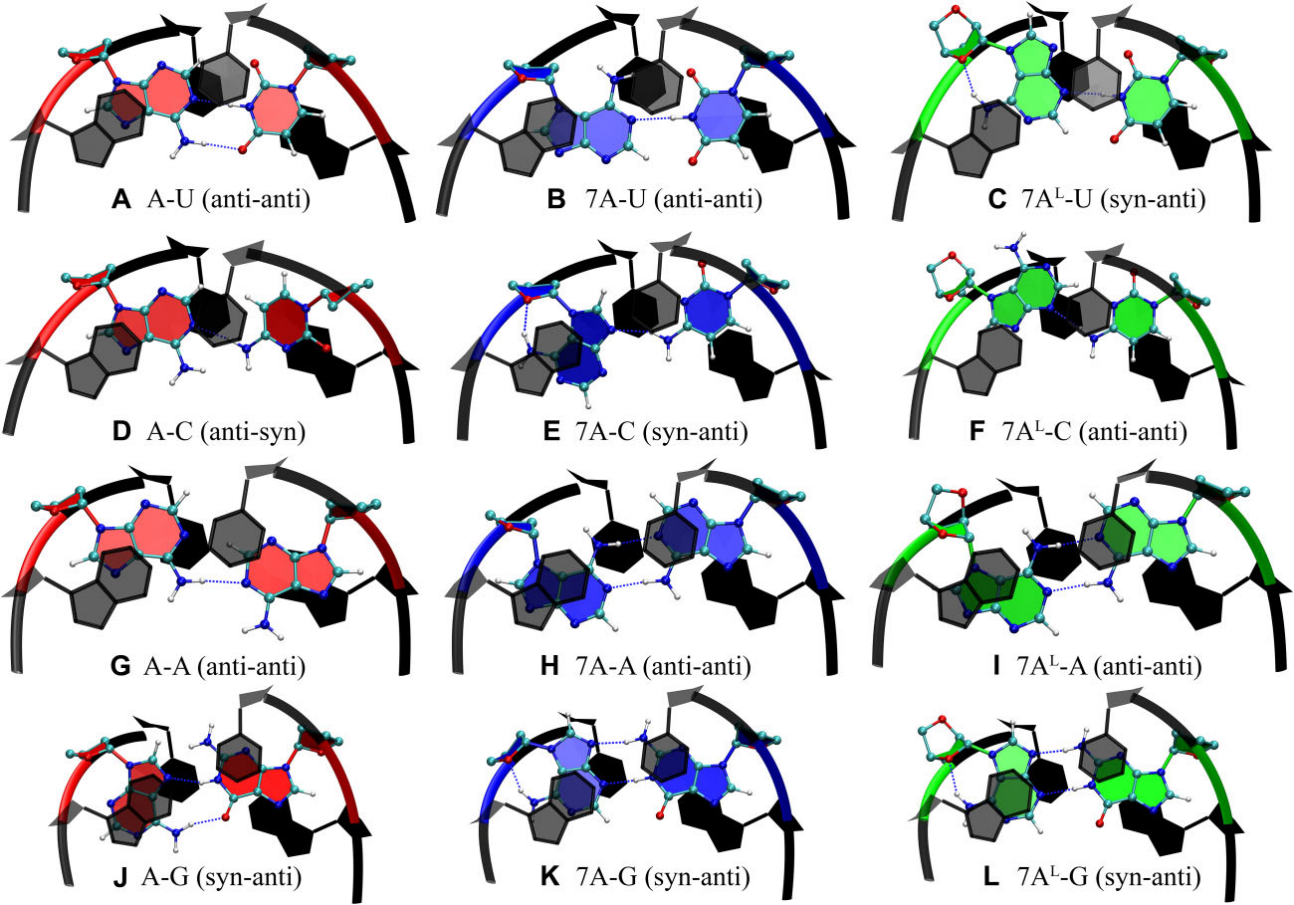
Predicted conformations of middle base pairs in (**A**) A-U, (**B**) 7A-U, (**C**) 7A^L^-U, (**D**) A-C, (**E**) 7A-C, (**F**) 7A^L^-C, (**G**) A-A, (**H**) 7A-A, (**I**) 7A^L^-A, (**J**) A-G, (**K**) 7A-G and (**L**) 7A^L^-G. Red, blue and green are used to highlight base pairs formed by A, 7A and 7A^L^, respectively. Base pairs highlighted in black represent the closing GC base pairs with transparent ones illustrating the top GC base pairs. Dashed blue lines represent the hydrogen bonds. Note that due to inversion of adenine in 7A and 7A^L^, functional groups are re-located, which either can destabilize the system as observed in B and C by losing a hydrogen bond or stabilize the system as observed in H, I, K and L by forming extra hydrogen bonds (Table 2).

Figure 6A–F displays the global minimum structures predicted for the middle base pairs in A-U, 7A-U, 7A^L^-U, A-C, 7A-C and 7A^L^-C by MD calculations (Table 2). As expected, the middle 1 × 1 A:U base pair in A-U is forming 1.98 hydrogen bonds in *anti*-*anti* orientation (Table 2 and Supplementary Table S12). When A is substituted with 7A and 7A^L^ as it is seen in 7A-U and 7A^L^-U, respectively, the average number of hydrogen bonds between the middle base pairs drops to 1.16 and 1.00 (Table 2 and Figure 6A–C). Analyses also display that 1 × 1 7A:U mismatch is flexible and can form *anti*-*anti* and *syn*-*anti* states with relatively similar Δ*G*_pred_ values (Supplementary Table S13). Furthermore, 7A^L^ in 7A^L^-U is in *syn* orientation, which is due to the interaction of its amino group, -NH_2_, with its own O4′ atom (Figure 6C). Due to N7-glycosylation of adenine in 7A and 7A^L^, the amino groups in these residues can interact with the phosphate backbone or with its own O4′ atom by switching to the *syn* orientation as seen in 1 × 1 7A^L^:U mismatch (Figure 6C). Nevertheless, the *anti* orientation is the favored state in RNA over the *syn* orientation, as the transformation from *anti* to *syn* will destabilize the system unless accommodated by additional hydrogen bonds (45). This is exemplified in 1 × 1 G:G internal loops, which prefer *syn*-*anti* orientations and form two hydrogen bonds (67–70). In 1 × 1 7A^L^:U, however, one hydrogen bond is totally lost when 7A^L^ is in *syn* orientation, resulting in reduced stability compared with 1 × 1 A:U (Figure 6A and C and Table 2).

Predicted structure of A-C displays the middle 1 × 1 A:C base pair forming a single hydrogen bond where C is in *syn* orientation (Figure 6D). It is noteworthy to highlight here that 1 × 1 A:C internal loop is a flexible motif, where cluster analysis displays both *anti*-*syn* and *anti*-*anti* orientations forming single hydrogen bonds with relatively similar Δ*G*_pred_ values (Supplementary Table S15). Nevertheless, 7A-C presents a 1 × 1 7A:C mismatch, where 7A is in *syn* orientation forming on average 0.97 hydrogen bonds (Table 2 and Figure 6E). Like A-C, 1 × 1 7A:C mismatch is dynamic, which can also form *anti*-*anti* orientations with relatively similar Δ*G*_pred_ values and single hydrogen bonds (Supplementary Table S16). Similar to the case observed in 7A^L^-U, the *anti* → *syn* transformation does not create an extra hydrogen bond with the complementary strand to stabilize the duplex, which explains why ΔΔ*G*°_37_ between 7A-C and A-C is observed to be 2.07 kcal mol^−1^ and ΔΔ*G*_pred_ is predicted to be 1.17 kcal mol^−1^ (Table 2). The predicted global minimum structure of 7A^L^-C displays a similar scenario (Supplementary Table S17). Although cluster analyses display this system to have *anti*-*anti*, *syn*-*syn*, *syn*-*anti* and *anti*-*syn* orientations with relatively similar Δ*G*_pred_ values with single hydrogen bonds (Supplementary Table S17), predicted global minimum displays 1 × 1 7A^L^:C in *anti*-*anti* orientation preserving the A-form RNA orientation (Figure 6F). This might be the reason why the ΔΔ*G*°_37_ and ΔΔ*G*_pred_ values to be marginal, with values of 0.39 and 0.42 kcal mol^−1^, respectively (Table 2).

### When paired with adenosine, 7A and 7A^L^ stabilize the RNA duplexes by forming base pairs with two hydrogen bonds in *anti*-*anti* orientations

Previous studies showed that 1 × 1 A:A base pairs form a single hydrogen bond state in *anti*-*anti* orientations (71–75). Our cluster analyses and binding studies also determined that the global minimum state of the 1 × 1 A:A mismatch in A-A display similar properties (Figure 6G and Table 2, and Supplementary Table S18). When one of the A is substituted with either 7A or 7A^L^, however, the 1 × 1 loop forms a two-hydrogen bond state in *anti*-*anti* orientation, where each amino group is forming a hydrogen bond with the opposing basic nitrogen located at N1 position (Figure 6H and I, and Supplementary Tables S19 and S20). Calculations are also in line with the NMR results predicting 1 × 1 7A:A and 7A^L^:A mismatches in *anti*-*anti* orientations (Table 2). The predicted global minimum structures extracted from MD calculations are also in agreement with the measured NOE patterns (Supplementary Tables S24 and S25). The formation of an extra hydrogen bond with no observable distortions at the backbone as seen in Figure 6H and I is the reason why 7A-A and 7A^L^-A are forming more stable duplexes compared with A-A (Table 2).

### When paired with guanosine, 7A and 7A^L^ forms two hydrogen bond states in *syn*-*anti* orientations similar to the structures observed in 1 × 1 G:G internal loop motifs

Cluster analyses determined that the 1 × 1 A:G internal loop in A-G prefers *syn*-*anti* orientation forming two hydrogen bonds between the amino group of A and carbonyl group of G, and basic nitrogen of A located at N7 and imino group of G (Figures 1 and 6J, and Supplementary Table S21). The 1 × 1 7A:G and 1 × 1 7A^L^:G mismatches also form two hydrogen bond states in *syn*-*anti* orientations, where the two basic nitrogen atoms of 7A and 7A^L^ located at N3 and N9 form hydrogen bonds with the amino and imino groups of G, respectively (Figure 6K and L and Table 2, and Supplementary Tables S22–S23). These structural predictions display 1 × 1 7A:G and 7A^L^:G loops in *syn*-*anti* orientations in agreement with the NMR observations (Supplementary Tables S26 and S27). This state is very similar to the non-canonical base pairs observed in 1 × 1 G:G internal loop motifs except the location of amino groups (67–70). In 1 × 1 G:G internal loops, one of G is in *syn* orientation, which displays an intramolecular salt bridge between amino group and phosphate backbone, as well as two hydrogen bonds with the opposite G. As described above, when 7A and 7A^L^ are in *syn* orientations, the amino groups are not forming intramolecular interactions with the phosphate backbone but the O4′ atoms that could stabilize the duplexes further (Figure 6K and L).

### Limitations of MD calculations

Here, it is worth looking at the limitations of the MM/3D-RISM binding energy calculations used to predict the structure and thermodynamics of the studied systems. These calculations, performed on an RNA duplex, aim to predict a global minimum by optimizing intermolecular stacking and hydrogen bonding interactions. However, they do not account for the formation of intramolecular salt bridges, such as those observed in the *anti*-*syn* states of 1 × 1 A:G, nor do they consider the effects of backbone distortions caused by base orientations (*syn*-*anti* versus *anti*-*syn*) or changes in intramolecular stacking interactions. For instance, while the predicted loop configuration of 1 × 1 7A:G aligns well with NMR data, the predicted binding (hybridization) energy suggests that 7A-G is 0.71 kcal mol^−1^ more stable than A:G, whereas experimental data shows the opposite (Tables 1 and 2). Specifically, the binding energy calculations overstabilize 7A-G by ΔΔ*G*°_37_ − ΔΔ*G*_pred_ = 1.70 kcal mol^−1^ (Table 2). Similar overestimations are also observed in 7A-U, 7A^L^-U, 7A-C and 7A-A, with overstabilizations by 0.58, 1.21, 0.90 and 1.25 kcal mol^−1^, respectively (Table 2). These discrepancies likely arise because MM/3D-RISM calculations omit intramolecular interactions, and the potential strains imposed on the backbone by specific loop conformations. Additionally, previous NMR studies on 1 × 1 A:G internal loops showed that these loops form one hydrogen bond in the *anti*-*syn* orientation, contrary to our predictions (76,77). Our cluster analyses also indicate that the *anti*-*syn* orientations are stable, but with stabilities <2.5 kcal mol^−1^ compared with the predicted global minimum structure, which is in a *syn*-*anti* orientation (Supplementary Table S21). As a result, binding energy calculations that do not account for changes in intramolecular interactions may yield predictions that do not fully capture the experimental data, thus limiting their accuracy.

### Reproducibility of UV-melting data

The SDs displayed in Table 1 are not formal ‘errors’ in the sense of absolute uncertainty. This reflects the variability in the measured Δ*G*°_37_ values from multiple melting curve analyses, giving an indication of how consistent the measurements are. It does not represent a systematic error or absolute uncertainty due to experimental factors like instrument calibration or sample preparation. Instead, it shows the spread of values obtained from fitting multiple datasets or replicates. Its practical implication is that if one sees a small ‘error’ value in MeltWin, it means that the measurements are consistent across replicates or fitting methods, indicating a reliable estimate of Δ*G*°_37_. A larger ‘error’ value might suggest variability in the melting curves, possibly due to experimental conditions or inconsistencies in curve fitting. To further assess reproducibility, we performed additional UV-melting measurements on eight of the systems listed in Table 1 (see Supplementary Table S28). The results demonstrate that the Δ*G*°_37_ values obtained from this extended dataset are consistent with the trends observed in Table 1, with values within 3% of the corresponding average values.

## Discussion

Although mixtures of N9- and N7-regioisomers are obtained during the chemical synthesis of adenosine, it remains unclear why nature would favor N9-adenosine over N7-adenosine. During the prebiotic RNA world era, nature likely favored RNA molecules that formed thermodynamically stable structures such as N9-regioisomers of adenosine, uridine, cytidine and guanosine, allowing the formation of Watson–Crick AU and GC base pairs. Furthermore, RNA recognition, transcription, splicing and translation, which are important in cell biology, might be enhanced when N9-regioisomers are utilized in the cell. Further evolutionary studies on the functional roles of N9-regioisomers could help the design of synthetic RNA analogs with enhanced properties.

There are numerous modified RNA residues available in the literature where specific sugar and base atoms are modified to manipulate and control the properties of RNA molecules (78–81). These modifications can enhance the stability and biostability of RNA, enhancing specificity and selectivity (80,82). Furthermore, they are utilized in structural studies to stabilize RNA molecules while investigating specific RNA motifs. Moreover, modified RNA residues are used as molecular probes to study RNA structure–function relationships and to describe the mechanism behind RNA-based regulatory processes (83,84). Thus, understanding the structural effects of modified RNA residues will be crucial when utilizing them in biotechnological applications and therapeutic interventions.

Modification of glycosidic linkage from N9 to N7 as observed in 7A and 7A^L^ results in almost 180° inversion of adenine, which exposes different functional groups and basic nitrogen atoms located at N1, N3 and N9 accessible to form hydrogen bonds with the complementary strands (Supplementary Tables S3 and S4). While such modification can disrupt canonical A-form RNA structure, it can also create novel RNA motifs with non-canonical base pairs enhancing the thermodynamic properties. Differences in the sugar pucker and/or the orientation of the N-glycoside bond will cause structural and thermodynamic differences in A→7A and A^L^→7A^L^ mutations. As a result, it is expected that substitution of A and A^L^ with 7A and 7A^L^ will yield different properties as we discuss in this contribution. A comprehensive analysis on modified RNA residues is crucial to potentially understand their roles in RNA folding, recognition and catalysis. Furthermore, advancement in computer technology, combined with experimental techniques, will facilitate the characterization of modified RNA structures, which could be utilized in RNA-based therapeutics and/or structural studies.

The extended repertoire of RNA residues offers diverse applications, including the use of N1-methylguanosine (m^1^G) in structural studies which, when paired with guanosine residues, will specifically stabilize 1 × 1 m^1^G:G mismatches in *syn*-*anti* orientations (85). In this context, the structural and thermodynamic properties of 7A and 7A^L^ present unique opportunities in structural studies. For example, 1 × 1 A:A internal loops observed in RNA CAG repeat expansions causing Huntington’s disease are known to interact with muscleblind-like 1 protein (MBNL1). This interaction provides an opportunity to use 7A and 7A^L^ to investigate the specifics of RNA CAG/MBNL1 interactions, such as how the zinc finger domains of MBNL1 recognize and bind to 1 × 1 A:A internal loops within CAG repeats. Furthermore, 1 × 1 G:G internal loops are known to form *syn*-*anti* orientations, a structural feature observed in RNA CGG and G_4_C_2_ repeat expansions, linked to fragile X-associated tremor/ataxia syndrome, amyotrophic lateral sclerosis and frontotemporal dementia (85). By mutating one of the guanosines in the 1 × 1 G:G pair to 7A, the 1 × 1 G:G (which prefers *anti*-*syn*) can be transformed into 1 × 1 7A:G (which prefers *syn*-*anti*), allowing control over the orientation of G in NMR studies. Such mutation studies can provide invaluable help in structural studies, such as deciphering specific chemical shifts in NMR experiments, which are hard to deconvolute otherwise.

## Supplementary Material

gkae1222_Supplemental_File

## Data Availability

All data are contained within the manuscript and/or supplementary files. Computational data that support the findings of this study are available from the corresponding author upon reasonable request.
