# Supplementary material for: Experimental and computational investigations of RNA duplexes containing N7-regioisomers of adenosine and LNA-adenosine
Source: Nucleic Acids Res. 2024 Dec 23;53(1):gkae1222. doi: 10.1093/nar/gkae1222 (PMC11724317; doi:10.1093/nar/gkae1222)
Supplement: gkae1222_Supplemental_File [file gkae1222_supplemental_file.docx]

Supplementary Information

Experimental and computational investigations of RNA duplexes containing N7-regioisomers of adenosine and LNA-adenosine

**Ilyas Yildirim^1*^**^†^**, Witold Andralojc^2^**^†^**, Amirhossein Taghavi^1,3^, Daniel Baranowski^2^, Zofia Gdaniec^2^, Ryszard Kierzek^2*^, Elzbieta Kierzek^2*^**

^1^ Department of Chemistry and Biochemistry, Florida Atlantic University, Jupiter, FL 33458 USA

^2^ Institute of Bioorganic Chemistry, Polish Academy of Sciences, Noskowskiego 12/14, 61-704 Poznan, Poland

^3^ Department of Chemistry, The Scripps Research Institute, 130 Scripps Way, Jupiter, FL 33458 USA

† These authors contributed equally to this work

* Authors to whom correspondence should be addressed:

[iyildirim@fau.edu](mailto:iyildirim@fau.edu)

rkierzek@ibch.poznan.pl

elzbieta.kierzek@ibch.poznan.pl

| **Table of Contents** | | |
| --- | --- | --- |
| **Supplemental Results.** Synthesis of protected N7-regioisomer of adenosine and its phosphoramidite - Extended. | | **p. S5-S6** |
| **Supplemental Results.** NMR spectra of 7A and 7A^L^. | | **p. S7-S8** |
| **Script S1** | Script used to perform hydrogen bond analyses. | **p. S9-S10** |
| **Script S2** | In-house PERL script to perform cluster analyses. | **p. S11-S13** |
| **Script S3** | Sample script used to perform MM/3D-RISM and NMODE analyses. | **p. S14** |
| **Table S1** | Mass spectrometry (MALDI) analyses of RNA oligonucleotides. | **p. S15** |
| **Table S2** | Experimental and literature ^13^C NMR data for A, 7A and 7A^L^. | **p. S16** |
| **Table S3** | Atom names, types, and RESP charges of 7A. | **p. S17** |
| **Table S4** | Atom names, types, and RESP charges of 7A^L^. | **p. S18** |
| **Table S5** | Details of each RNA system studied computationally. | **p. S19** |
| **Table S6** | Sample input files used in minimization, equilibration, and production runs. | **p. S20** |
| **Table S7** | Experimental and literature ^1^H NMR data for A, 7A and 7A^L^. | **p. S21** |
| **Table S8** | Experimental coupling constants (^1^H-^1^H and ^13^C-^1^H), pseudorotational parameters, and populations of sugar conformations of A, 7A, and 7A^L^. | **p. S22** |
| **Table S9** | Structurally significant NOEs observed in 1×1 mismatches. | **p. S23** |
| **Table S10** | Chemical shifts of aromatic/anomeric protons in 7A-G and 7A^L^-G. | **p. S24** |
| **Table S11** | Chemical shifts of aromatic/anomeric protons in 7A-A and 7A^L^-A. | **p. S25** |
| **Table S12** | Binding free energy calculations of A-U. | **p. S26** |
| **Table S13** | Binding free energy calculations of 7A-U. | **p. S27** |
| **Table S14** | Binding free energy calculations of 7A^L^-U. | **p. S28** |
| **Table S15** | Binding free energy calculations of A-C. | **p. S29** |
| **Table S16** | Binding free energy calculations of 7A-C. | **p. S30** |
| **Table S17** | Binding free energy calculations of 7A^L^-C. | **p. S31** |
| **Table S18** | Binding free energy calculations of A-A. | **p. S32** |
| **Table S19** | Binding free energy calculations of 7A-A. | **p. S33** |
| **Table S20** | Binding free energy calculations of 7A^L^-A. | **p. S34** |
| **Table S21** | Binding free energy calculations of A-G. | **p. S35** |
| **Table S22** | Binding free energy calculations of 7A-G. | **p. S36** |
| **Table S23** | Binding free energy calculations of 7A^L^-G. | **p. S37** |
| **Table S24** | Comparison of NOEs to predicted distances for each cluster in 7A-A. | **p. S38** |
| **Table S25** | Comparison of NOEs to predicted distances for each cluster in 7A^L^-A. | **p. S39** |
| **Table S26** | Comparison of NOEs to predicted distances for each cluster in 7A-G. | **p. S40** |
| **Table S27** | Comparison of NOEs to predicted distances for each cluster in 7A^L^-G. | **p. S41** |
| **Table S28** | Extended UV-melting data. | **p. S42** |
| **Figure S1** | ^1^H NMR spectrum of 7-β-D-ribofuranosyladenine in DMSO-d_6_. | **p. S43** |
| **Figure S2** | ^13^C NMR spectrum of 7-β-D-ribofuranosyladenine in DMSO-d_6_. | **p. S44** |
| **Figure S3** | ^1^H-^1^H COSY spectrum of 7-β-D-ribofuranosyladenine in DMSO-d_6_. | **p. S45** |
| **Figure S4** | ^1^H-^13^C gHSQC spectrum of 7-β-D-ribofuranosyladenine in DMSO-d_6_. | **p. S46** |
| **Figure S5** | ^1^H-^13^C gHMBC spectrum of 7-β-D-ribofuranosyladenine in DMSO-d_6_. | **p. S47** |
| **Figure S6** | ^1^H NMR spectrum of 2′-O,4′-C-Methylene-7-β-D-ribofuranosyladenine in DMSO-d_6_. | **p. S48** |
| **Figure S7** | ^13^C NMR spectrum of 2′-O,4′-C-Methylene-7-β-D-ribofuranosyladenine in DMSO-d_6_. | **p. S49** |
| **Figure S8** | ^1^H-^13^C gHSQC spectrum of 2′-O,4′-C-Methylene-7-β-D-ribofuranosyladenine in DMSO-d_6_. | **p. S50** |
| **Figure S9** | ^1^H-^13^C gHMBC spectrum of 2′-O,4′-C-Methylene-7-β-D-ribofuranosyladenine in DMSO-d_6_. | **p. S51** |
| **Figure S10** | ^1^H NMR spectrum of 9-β-D-ribofuranosyladenine in DMSO-d_6_. | **p. S52** |
| **Figure S11** | ^13^C NMR spectrum of 9-β-D-ribofuranosyladenine in DMSO-d_6_. | **p. S53** |
| **Figure S12** | ^1^H-^13^C gHSQC spectrum of 9-β-D-ribofuranosyladenine in DMSO-d_6_. | **p. S54** |
| **Figure S13** | ^1^H-^13^C gHMBC spectrum of 9-β-D-ribofuranosyladenine in DMSO-d_6_. | **p. S55** |
| **Figure S14** | Fragment of^1^H-^13^C gHMBC spectrum showing long range ^1^H-^1`3^C correlations crucial for determination of D-ribofuranosyl moiety position in adenine. | **p. S56** |
| **Figure S15** | Imino regions in 1D ^1^H NMR spectra recorded at four different temperatures for the duplex containing 7A^L^:G mismatch. | **p. S57** |
| **Figure S16** | Imino-imino and imino-amino/aromatic regions of the 2D ^1^H-^1^H NOESY spectra recorded for 7A-G and 7A^L^-G mismatches. | **p. S58** |
| **Supplemental References** | | **p. S59** |

**Supplemental Results.**

**Synthesis of protected N7-regioisomer of adenosine and its phosphoramidite - Extended.** N7-regioisomer of adenosine (7A) was synthesized according to published procedure with some modifications.(1) Adenine was suspended in HMDS (4 mL/1 mmol adenine) and 10 mg of ammonium sulfate per 1 mmol of adenine was added and refluxed over 16 h. Reaction mixture was then evaporated, dissolved in anhydrous acetonitrile (4 mL/1 mmol scale of synthesis), combined with ß-D-ribofuranose 1,2,3, 5-tetraacetate (1.5 equivalent) and trimethylsilyl trifluoromethanesulfonate (TMSOTf, 1.5 equivalent), and finally stirred at room temperature (RT) for 80 min. After performing thin layer chromatography (TLC) analysis, a saturated aqueous solution of sodium bicarbonate was added to the reaction mixture, which was then extracted three times with dichloromethane. The combined organic layers were dried over anhydrous sodium sulfate, filtered, and evaporated. Silica gel column chromatography purification was performed, and acetylated N7-adenosine was isolated with an approximate yield of 60%. The acetylated N7-adenosine was initially dissolved in methanol (2 mL per 1 mmol) and subsequently mixed with 25% aqueous ammonia (4 mL per 1 mmol). After TLC analysis confirmed the complete removal of acetyl groups within 2 h at RT, the reaction mixture was first evaporated and then co-evaporated with pyridine three times. Afterwards, the mixture was co-evaporated with anhydrous methanol three times. The resulting product was then suspended in anhydrous methanol (3 mL per 1 mM scale of synthesis). N,N-dimethylformamide dimethyl acetal (2.5 equivalents per 1 mmol substrate) was added to the reaction mixture, and the mixture was stirred for 48 h. Once the reaction completion was confirmed by TLC, water was added to the mixture, which was then evaporated after 15 min. After co-evaporating the reaction mixture with anhydrous pyridine three times, dimethoxytrityl chloride (1.05 equivalents per 1 mmol used for the synthesis of N7-adenosine) was added to the mixture and stirred at RT for 2 h. Again, after confirming reaction completion by TLC, a saturated aqueous solution of sodium bicarbonate was added to the mixture, which was then extracted three times with dichloromethane. The combined organic layers were dried over anhydrous sodium sulfate, filtered, and evaporated. Solution was first evaporated and co-evaporated with toluene. The reaction mixture was purified by silica gel column chromatography using a dichloromethane eluent with a methanol gradient. The overall yield of the last three steps was approximately 50%. The final step was protection of 2′-hydroxyl with tert-butyldimethylsilyl protecting group. Product was evaporated three times with anhydrous pyridine and dissolved in anhydrous pyridine (5 mL/1 mmol). To the solution of 5′-O-dimethoxytrityl-N6-dimethylformamidine-N7-adenosine, tert-butyldimethylsilyl chloride (1.2 equivalents) and imidazole (2.5 equivalents) were added, and the mixture was stirred for 16 h at RT. After verifying the completion of the reaction with TLC, an aqueous solution of sodium dihydrogen phosphate was added to the mixture, which was then extracted three times with dichloromethane. The combined organic layers were extracted with aqueous solution of sodium bicarbonate, dried over anhydrous sodium sulfate, filtered, and evaporated. The solution was co-evaporated with toluene three times before being purified by silica gel column chromatography using a dichloromethane eluent with a gradient of ethyl acetate. The yield of the synthesis of the 2′-silyl 7N-adenosine derivative was approximately 45%. The structure of N7-adenosine was verified through 1D and 2D ^1^H and ^13^C NMR experiments (see SI, section "NMR spectra of 7A and 7A^L^”). Protected 3′-O-phosphoramidite of N7-A was prepared according to published procedures using 2-cyanoethyl N,N,N′,N′-tetraisopropylphosphorodiamidite and equivalent amount of tetrazole.(2,3) The yield of the phosphoramidite after silica gel column chromatography was approximately 80%.

**NMR spectra of 7A and 7A^L^.** Structures of 7A and 7A^L^ were confirmed by a series of ^1^H and ^13^C NMR experiments, where the chemical shift data of 7A was observed to be in good agreement with literature.(1,4,5) Additionally, assignment of ^1^H and ^13^C resonances of 7A and 7A^L^ was confirmed by the analysis of 2D spectra (^1^H−^1^H COSY, ^1^H−^13^C HSQC and HMBC), which were compared to adenosine (**Tables S2** and **S7, Figures S1-S14**). Conformational analysis was carried out based on vicinal ^1^H–^1^H and heteronuclear ^13^C-^1^H coupling constants as well as NOE measurements. (**Tables S8** and **S9**)

As previously reported, ribosylation of adenine in position 7 or 9 caused significant differences in ^13^C and ^1^H resonances, especially carbons of C4, C5, C6 and C8 and protons of H2′, H6 and H8 (**Tables S2** and **S7**). Conspicuous downfield shift nearly by 10 ppm for C4 and upfield shift by 9 and 4 ppm for C5 and C6, respectively, were observed that were characteristic pattern of changes of ^13^C resonances for N7-regioisomer of adenosine compared to N9-regioisomer. The same patterns in ^13^C chemical shift changes were also observed in 7A^L^ (**Table S2**). In the case of ^1^H resonances, an upfield shift by ca 0.5 ppm and 0.3 ppm were observed in H2′ and H6, respectively (**Table S7**). Interestingly, the small but opposite effect by ca 0.1 ppm was observed in H2′ of 7A^L^ that might be attributed to fixed i.e. C3′-*endo* conformation of the sugar moiety. The characteristic heteronuclear long-range correlations i.e. C5-H1′ and C8-H1′ observed on ^1^H-^13^C gHMBC spectra (**Figure S14**) were crucial for unambiguous determination of D-ribofuranosyl substitution at N7 position of adenine.

In order to estimate the effects of ribosylation of adenine in position 7 or 9 on sugar puckers and *syn*-anti equilibrium in 7A and 7A^L^, we subjected them to a conformational analysis by means of NMR spectroscopy and PSEUROT calculations approach (**Table S8**).(6,7) Generally, β-D-ribofuranose moiety A and 7A showed predominant preference for S-type sugar puckering, however significant differences in conformer types for N7- and N9-regioisomers of adenosine were observed (**Table S8**). The ribose moiety in adenosine preferred C2′-*endo* and C2′-*endo*- C1′-*exo* sugar puckers where the proportion of the total population were in the range of 64-74%. In case of 7A, the very strong preference for C1′-*exo* conformation were observed, which is the same conformation as obtained from crystallographic structure.(1) On the other hand, the sugar conformation of 7A^L^ adopted N-type sugar puckering i.e. C3′-*endo* conformation as a result of methylene bridge between C4′ and O2′ of ribose moiety. In addition, estimation of N↔S equilibrium by means of simple equation (**Table S8**) confirmed significant predominance of S-type conformers; however, the obtained values needed to be regarded as very approximate compared with those obtained with PSEUROT approach. Conformation around the glycosidic bond (χ) i.e. base orientation with respect to sugar part was determined by observing the NOE enhancement of H1′ and H2′ protons upon irradiation of H8 and by measuring the heteronuclear vicinal coupling constants of ^3^J_H1′C8_ and J_H1′C4_ /J_H1′C5_. Generally, in both 7A and 7A^L^, NOE enhancement was stronger in H2′ proton (1.7%) compared to H3′ thus indicating for preference of *anti*-conformation. However, for H3′ different values of NOE were observed i.e. 1.3% vs 0.9% in 7A^L^ and 7A, respectively that may be derived from axial orientation of C3′-H3′ bond in C3′-*endo* or pseudo axial in C2′-*endo* conformation of sugar part. Approximate values of glycosidic bond angle were extracted from the analysis of ^3^J_H1′C8_ and J_H1′C4_/J_H1′C5_ based on parameter set for the Karplus equations estimated by Ippel et al.(8) The higher values of ^3^J_H1′C8_ compared to J_H1′C5_ confirms the *anti*-conformation with χ torsional angles around 190-220°. Nevertheless, low values of J_H1′C5_ observed for 7A and 7A^L^ may also suggest base moiety to prefer *high-anti* conformation with χ torsional angles around 270°. For example, the crystallographic data of 7A^L^ unequivocally demonstrated orientation of adenine in the *high-anti* region.(1)

**Script S1.** Script used to perform hydrogen bond analyses.

#!/bin/bash

#

res1=`**pwd** | awk '{s=split($1,a,"/"); for(i=1; i<=s; i++){if(a[i] ~ /^anal/){split(a[i],b,"_"); split(b[2],c,"-"); print c[1]}}}'`

res2=`**pwd** | awk '{s=split($1,a,"/"); for(i=1; i<=s; i++){if(a[i] ~ /^anal/){split(a[i],b,"_"); split(b[2],c,"-"); print c[2]}}}'`

#

cat << EOF > ptraj_hbond_anal.in

parm ../../prmtop.complex

trajin ../mdcrd.netcdf

EOF

#

**if** **[[** $res2 =~ "A" ]]; **then**

cat << EOF >> ptraj_hbond_anal.in

hbond hbond_WC1 out hbond_btw_3-8_wc1.txt angle 45 dist 3.5 donormask :3@N6 donorhmask :3@H61 acceptormask :8@N1,N3,N7 avgout hbond_btw_3-8_wc1.avg.dat series uuseries hbond_btw_3-8_wc1

hbond hbond_WC2 out hbond_btw_3-8_wc2.txt angle 45 dist 3.5 donormask :3@N6 donorhmask :3@H62 acceptormask :8@N1,N3,N7 avgout hbond_btw_3-8_wc2.avg.dat series uuseries hbond_btw_3-8_wc2

hbond hbond_WC3 out hbond_btw_3-8_wc3.txt angle 45 dist 3.5 donormask :8@N6 donorhmask :8@H61 acceptormask :3@N1,N3,N7,N9 avgout hbond_btw_3-8_wc3.avg.dat series uuseries hbond_btw_3-8_wc3

hbond hbond_WC4 out hbond_btw_3-8_wc4.txt angle 45 dist 3.5 donormask :8@N6 donorhmask :8@H62 acceptormask :3@N1,N3,N7,N9 avgout hbond_btw_3-8_wc4.avg.dat series uuseries hbond_btw_3-8_wc4

EOF

cpptraj <ptraj_hbond_anal.**in**> out_hbond_anal

#

v1=`paste hbond_btw_3-8_wc1.txt hbond_btw_3-8_wc2.txt | grep -v "#" | awk '{if((\$2 != 0)||(\$4 != 0)){c++}; s++}END{print c/s}'`

v2=`paste hbond_btw_3-8_wc3.txt hbond_btw_3-8_wc4.txt | grep -v "#" | awk '{if((\$2 != 0)||(\$4 != 0)){c++}; s++}END{print c/s}'`

total=`**echo** "$v1 $v2" | awk '{print \$1+\$2}'`

**elif** **[[** $res2 =~ "G" ]]; **then**

**if** **[[** $res1 =~ "7" ]]; **then**

total=`../../../analyze_WC_hbonds_7A-G.sh`

**else**

total=`../../../analyze_WC_hbonds_A-G.sh`

**fi**

**elif** **[[** $res2 =~ "U" ]]; **then**

cat << EOF >> ptraj_hbond_anal.in

hbond hbond_WC1 out hbond_btw_3-8_wc1.txt angle 45 dist 3.5 donormask :3@N6 donorhmask :3@H61 acceptormask :8@O2,O4 avgout hbond_btw_3-8_wc1.avg.dat series uuseries hbond_btw_3-8_wc1

hbond hbond_WC2 out hbond_btw_3-8_wc2.txt angle 45 dist 3.5 donormask :3@N6 donorhmask :3@H62 acceptormask :8@O2,O4 avgout hbond_btw_3-8_wc2.avg.dat series uuseries hbond_btw_3-8_wc2

hbond hbond_WC3 out hbond_btw_3-8_wc3.txt angle 45 dist 3.5 donormask :8@N3 donorhmask :8@H3 acceptormask :3@N1,N3,N7,N9 avgout hbond_btw_3-8_wc3.avg.dat series uuseries hbond_btw_3-8_wc3

EOF

cpptraj <ptraj_hbond_anal.**in**> out_hbond_anal

v1=`paste hbond_btw_3-8_wc1.txt hbond_btw_3-8_wc2.txt | grep -v "#" | awk '{if((\$2 != 0)||(\$4 != 0)){c++}; s++}END{print c/s}'`

v2=`cat hbond_btw_3-8_wc3.txt | grep -v "#" | awk '{s++; t+=\$2}END{print t/s}'`

total=`**echo** "$v1 $v2" | awk '{print \$1+\$2}'`

**elif** **[[** $res2 =~ "C" ]]; **then**

cat << EOF >> ptraj_hbond_anal.in

hbond hbond_WC1 out hbond_btw_3-8_wc1.txt angle 45 dist 3.5 donormask :3@N6 donorhmask :3@H61 acceptormask :8@O2,N3 avgout hbond_btw_3-8_wc1.avg.dat series uuseries hbond_btw_3-8_wc1

hbond hbond_WC2 out hbond_btw_3-8_wc2.txt angle 45 dist 3.5 donormask :3@N6 donorhmask :3@H62 acceptormask :8@O2,N3 avgout hbond_btw_3-8_wc2.avg.dat series uuseries hbond_btw_3-8_wc2

hbond hbond_WC3 out hbond_btw_3-8_wc3.txt angle 45 dist 3.5 donormask :8@N4 donorhmask :8@H41 acceptormask :3@N1,N3,N7,N9 avgout hbond_btw_3-8_wc3.avg.dat series uuseries hbond_btw_3-8_wc3

hbond hbond_WC4 out hbond_btw_3-8_wc4.txt angle 45 dist 3.5 donormask :8@N4 donorhmask :8@H42 acceptormask :3@N1,N3,N7,N9 avgout hbond_btw_3-8_wc4.avg.dat series uuseries hbond_btw_3-8_wc4

EOF

cpptraj <ptraj_hbond_anal.**in**> out_hbond_anal

v1=`paste hbond_btw_3-8_wc1.txt hbond_btw_3-8_wc2.txt | grep -v "#" | awk '{if((\$2 != 0)||(\$4 != 0)){c++}; s++}END{print c/s}'`

v2=`paste hbond_btw_3-8_wc3.txt hbond_btw_3-8_wc4.txt | grep -v "#" | awk '{if((\$2 != 0)||(\$4 != 0)){c++}; s++}END{print c/s}'`

total=`**echo** "$v1 $v2" | awk '{print \$1+\$2}'`

**fi**

#

**echo** $total

**Script S2.** In-house PERL script to perform cluster analyses.

#!/usr/bin/perl -w

#

# Written by Ilyas Yildirim (FAU) on July 24, 2022

#

# This script will uniquely cluster the conformations observed in MD trajectory. The lowest rmsd structures are then

# determined and clustered.

#

**use** Array::Utils qw(:all);

#

$mdcrd = "./mdcrd.nobox.nstep_10.strip_terminal_pairs.netcdf"; # Trajectory file we are analyzing.

$step = 100; # This is the step we will use to extract rst data from mdcrd, which will be used in rmsd calcs.

$cutoff = 0.5; # Cutoff for rmsd.

$refpdb = "reference.pdb"; # Reference .pdb file (which is used to create symmetric states).

$prmtop = "./prmtop.complex"; # Prmtop file

$pcheckf = "ptraj_check.in"; # Dummy ptraj check file.

###################################################################################################################

#

# Check out the size of the mdcrd file. Also, create the netcdf format. We will use this format because it is much faster than trajectory format.

#

$netcdf = "$mdcrd";

$size_mdcrd = check_size("$netcdf");

**print** "$size_mdcrd\n";

###################################################################################################################

#

# Also, check out the number of atoms in the $refpdb is the same as the number of atoms defined in the $prmtop file.

#

$system_size_from_ref = `cat $refpdb | grep ATOM | wc | awk '{print \$1}'`;

chomp($system_size_from_ref);

$system_size_from_prmtop = `grep -A 2 \"%FLAG POINTERS\" $prmtop | tail -1 | awk '{print \$1}'`;

chomp($system_size_from_prmtop);

**if**($system_size_from_ref != $system_size_from_prmtop){

**print** "System sizes defined in $refpdb and $prmtop do not match. Exit ...\n";

**die**;

}

###################################################################################################################

$RMScompound=""; # This is the compound's atom list we will use in RMSd calculation.

open(F2O_compound, "RMScompounds.txt") || die "cannot open RMScompounds.txt: $!";

**while**(<F2O_compound>){

chomp($_);

$RMScompound=$_;

}

**close**(F2O_compound) || die "cannot close RMScompounds.txt: $!";

#

$RMSloop=""; # This is the compound's atom list we will use in RMSd calculation.

open(F2O_compound, "RMSloop.txt") || die "cannot open RMSloop.txt: $!";

**while**(<F2O_compound>){

chomp($_);

$RMSloop=$_;

}

**close**(F2O_compound) || die "cannot close RMSloop.txt: $!";

#

###################################################################################################################

#

# So, we know what the size of the mdcrd is. Now, create a loop to extract the .rst files iteratively.

# Once an .rst is extracted from $mdcrd, the symmetric states will be first created. Later, we will calculate the rmsd.

#

$count = -1; # This variable is to count/create the number of cluster sets. Every cluster related array will use this

# number. Note that array index starts from 0!

@clustered = (); # This is an array which stores the clustered structures. It will be used to check out if a structure is

# already clustered or not.

#

LABEL_STEP:

**for**($i=$step; $i <= $size_mdcrd; $i+=$step){

#

# Check if this particular snapshot/step is already clustered.

#

**for**($j=0; $j <= $#clustered; $j++){

**if**($clustered[$j] == $i){

# We have already clustered $i step, so skip this point.

**next** LABEL_STEP;

}

}

$count++;

#

# We will do two calculations; first we will calculate the average structure of the initial cluster, and second we will

# finalize the clustering process.

#

**for**($l=1; $l <= 2; $l++){

**if**($l == 1){

#

# First phase of clustering: Reference structure is the snapshot taken from the trajectory file, and an initial

# clustering will be done yielding an average structure. This will be used in the second clustering process.

#

# Extract the structure/snapshot from trajectory for initial clustering process.

#

open(F2W, ">$pcheckf") || die "cannot open $pcheckf: $!";

**print** F2W "trajin $netcdf $i $i\n";

**print** F2W "trajout rst restart\n";

**close**(F2W) || die "cannot close $pcheckf: $!";

system("cpptraj $prmtop <$pcheckf> out_check");

system("rm $pcheckf out_check");

#

# Calculate the rmsd with respect to initial structure/snapshot extracted from the trajectory.

#

open(F2W, ">$pcheckf") || die "cannot open $pcheckf: $!";

**print** F2W "reference rst\n";

**print** F2W "trajin $netcdf\n";

**print** F2W "rms reference \"$RMScompound\" \n";

**print** F2W "rms reference out rms \"$RMSloop\" nofit\n";

#

system("cpptraj $prmtop <$pcheckf> out_rms");

system("rm $pcheckf out_rms");

#

# Create the structures satisfying the rmsd cutoff.

#

$line_str = `cat rms | grep -v "#" | awk -v cutoff=$cutoff '{if(\$2<=cutoff){l=l \$1" "}}END{gsub(/\ \$/,"",l); print l}'`;

chomp($line_str);

@tmp_str = split(/ /, $line_str);

#

@minus = array_minus(@tmp_str, @clustered);

#

open(F2W, ">cluster_$count\.txt") || die "cannot open cluster_$count\.txt: $!";

**for**($j=0; $j <= $#minus; $j++){

**print** F2W "$minus[$j]\n";

}

**close**(F2W) || die "cannot close cluster_$count\.txt: $!";

**print** "Step = $i\tCluster $count size = $#minus - Before the use of avg.rst\n";

#

# This is a new section for the code. Previously, the structures in the trajectory are used in clustering process.

# It is possible that sometimes that structure cannot be a good comparison. Thus, we will first find the average

# structure of this new cluster, and then re-do the cluster analyses. This way, structures missed in the first

# clustering process will be included.

#

# First, combine the cluster, and find the average str. The data will be located in the cluster_$count directory.

#

perform_1st_clustering("cluster_$count\.txt");

#

# Now, remove the files created during this process...

#

system("rm -f rst rms");

#

} **elsif** ($l == 2){

#

# We are in the second phase of the clustering where we will use the average structure as the reference conformation

# during the clustering process...

#

# Copy the avg.rst structure from cluster_$count directory as the reference structure for the second phase.

#

system("cp ./cluster_$count/avg.pdb ./pdb");

#

# Now, delete the cluster_$count directory. This is used for initial purposes - to create the average structure.

#

system("rm -dfr cluster_$count");

#

# Store these rst.names.

#

open(F2W, ">$pcheckf") || die "cannot open $pcheckf: $!";

**print** F2W "reference pdb\n";

**print** F2W "trajin $netcdf\n";

**print** F2W "rms reference \"$RMScompound\" \n";

**print** F2W "rms reference out rms \"$RMSloop\" nofit\n";

**close**(F2W) || die "cannot close $pcheckf: $!";

system("cpptraj $prmtop <$pcheckf> out_rms");

#

$line_str = `cat rms | grep -v "#" | awk -v cutoff=$cutoff '{if(\$2<=cutoff){l=l \$1" "}}END{gsub(/\ \$/,"",l); print l}'`;

chomp($line_str);

@tmp_str = split(/ /, $line_str);

#

@minus = array_minus(@tmp_str, @clustered);

@clustered = (@clustered, @minus);

#

# Now, create the final cluster data for $count.

#

open(F2W, ">cluster_$count\.txt") || die "cannot open cluster_$count\.txt: $!";

**for**($j=0; $j <= $#minus; $j++){

**print** F2W "$minus[$j]\n";

}

**close**(F2W) || die "cannot close cluster_$count\.txt: $!";

**print** "Step = $i\tCluster $count size = $#minus - Final Version\n";

#

system("rm -f rst rms");

#

}

}

}

################################################### Subroutines ###################################################

**sub** check_size {

**my**($smdcrd) = (@_);

open(SF2O, ">$pcheckf") || die "cannot open $pcheckf: $!";

**print** SF2O "parm $prmtop\n";

**print** SF2O "trajin $smdcrd 1 1\n"; # Because this is for checking purposes, do not worry about 1 1. Ptraj will print

# the size of the trajectory anyways...

**print** SF2O "rms first :1\n"; # For sure there is at least one residue in the system, so the script will work.

**close**(SF2O) || die "cannot close $pcheckf: $!";

system("cpptraj <$pcheckf> out_check");

**my**($s_size_mdcrd) = `cat out_check | grep \"reading 1\" | awk '{gsub(/\\)/,""); print \$NF}'`;

chomp($s_size_mdcrd);

**return** $s_size_mdcrd;

}

**sub** perform_1st_clustering {

**my**($sfilecs) = (@_);

open(SF2W, ">", "cluster_mdcrd_extract_tmp.sh") || die "cannot open cluster_mdcrd_extract_tmp.sh: $!";

**print** SF2W "#!/bin/sh\n";

**print** SF2W "\n";

**print** SF2W "file=$sfilecs\n";

**print** SF2W "mdcrd=../\"$mdcrd\"\n";

**print** SF2W "prmtop=../\"$prmtop\"\n";

**print** SF2W "\n";

**print** SF2W "dir=`ls -l \$file | awk '{split(\$9,a,\".\"); print a[1]}'`\n";

**print** SF2W "\n";

**print** SF2W "mkdir -p \$dir\n";

**print** SF2W "\n";

**print** SF2W "cd \$dir\n";

**print** SF2W "\n";

**print** SF2W "cat ../\$file | \\\n";

**print** SF2W "awk -v mdcrd=\$mdcrd -v rmsatoms=\"$RMScompound\" '{ \n";

**print** SF2W " print \"trajin \"mdcrd\" \"\$1\" \"\$1;\\\n";

**print** SF2W "} END { \\\n";

**print** SF2W " print \"trajout mdcrd.netcdf netcdf\";\\\n";

**print** SF2W " print \"rms first \\\"\"rmsatoms\"\\\"\";\\\n";

**print** SF2W " print \"average avg.pdb pdb\";\\\n";

**print** SF2W "}' > ptraj_avg.in\n";

**print** SF2W "\n";

**print** SF2W "cpptraj \$prmtop < ptraj_avg.in > out_avg\n";

**print** SF2W "\n";

**print** SF2W "cd ../\n";

**close**(SF2W) || die "cannot close cluster_mdcrd_extract_tmp.sh: $!";

system("bash cluster_mdcrd_extract_tmp.sh; rm cluster_mdcrd_extract_tmp.sh");

}

**Script S3.** Sample script used to perform MM/3D-RISM and NMODE analyses.

#!/bin/sh

#

DO_PARALLEL="mpiexec -np $SLURM_NPROCS"

EXE=$AMBERHOME/bin/MMPBSA.py.MPI

#

# Write input file

#

cat > mmpbsa.**in** << EOF

MMPBSA.py input file for running PB and GB

&general

startframe=1, endframe=100000, interval=1,

keep_files=1, verbose =2,

/

&gb

igb=5, saltcon=0.300,surften=0.0072,surfoff=0.00,

/

&pb

istrng=0.300, fillratio=4.0,inp=1,radiopt=0,

indi=1.0,exdi=80,scale=2,linit=1000,prbrad=1.4,

cavity_surften=0.0072,cavity_offset=0.00,

/

&rism

closure="kh", rism_verbose=0, polardecomp=1, thermo="gf",

/

&nmode

nmode_istrng=0.30,maxcyc=1000,

/

EOF

#

# Execute the program

#

$DO_PARALLEL $EXE -O -i ./mmpbsa.**in** \

-sp ./prmtop.complex \

-cp ./prmtop.complex \

-rp ./prmtop.ss1 \

-lp ./prmtop.ss2 \

-o ./nmode.out \

-y ./mdcrd.netcdf > error.nmode 2>&1 || error

**Table S1**. Mass spectrometry (MALDI) analyses of RNA oligonucleotides described in **Table 1**.

| **Sequence^a^** | **Molecular weight**  **(MALDI measured)** | **Molecular weight (Calculated)** |
| --- | --- | --- |
| UCAGGCAGU | 2853.463 | 2853.422 |
| ACUGCCUGA | 2812.807 | 2814.760 |
| AUCAGGCAGU | 3183.550 | 3183.995 |
| UCAGGCAGUA | 3183.043 | 3183.995 |
| UCAGA^L^CAGU | 2852.756 | 2851.804 |
| UCAG7A^L^CAGU | 2852.747 | 2851.804 |
| ACUGACUGA | 2841.300 | 2839.793 |
| ACUGGCUGA | 2857.835 | 2855.793 |
| ACUGUCUGA | 2818.443 | 2816.753 |
| UCAG7ACAGU | 2841.365 | 2839.793 |
| UCAGACAGU | 2841.472 | 2839.793 |

^a^ 7A = N7-adenosine, A^L^ = LNA-adenosine, 7A^L^ = N7-LNA-adenosine.

**Table S2.** Experimental and literature ^13^C NMR data for A, 7A and 7A^L^ (units in *ppm*).

|  | **C6** | **C2** | **C4** | **C8** | **C5** | **C1′** | **C4′** | **C2′** | **C3′** | **C5′** |
| --- | --- | --- | --- | --- | --- | --- | --- | --- | --- | --- |
| **A** | 156.22 | 152.52 | 149.16 | 140.07 | 119.42 | 88.07 | 86.02 | 73.60 | 70.75 | 61.78 |
| **A^a^** | 157.1 | 153.9 | 148.8 | 141.3 | 121.0 | 89.3 | 87.2 | 74.8 | 71.9 | 62.9 |
| **A^b^** | 156.19 | 152.42 | 149.06 | 139.96 | 119.38 | 87.92 | 85.93 | 73.44 | 70.70 | 61.71 |
| **A^c^** | 156.86 | 153.16 | 149.84 | 140.72 | 120.09 | 88.75 | 86.65 | 74.26 | 71.41 | 62.41 |
| **7A** | 151.50 | 152.62 | 160.70 | 144.42 | 111.05 | 89.19 | 86.17 | 74.82 | 68.78 | 60.33 |
| **7A^a^** | 152.9 | 154.0 | 161.7 | 145.9 | 111.3 | 90.6 | 87.6 | 76.1 | 70.1 | 61.6 |
| **7A^b^** | 151.49 | 152.61 | 160.69 | 144.41 | 110.05 | 89.18 | 86.16 | 74.82 | 68.78 | 60.32 |
| **7A^L^** | 151.83 | 152.81 | 160.13 | 141.92 | 110.4 | 89.86 | 86.01 | 79.31 | 70.13 | 56.83 |

**^a^** Ref (4). **^b^** Ref (1). **^c^** Ref (5).

**Table S3.** Atom names, types, and RESP charges (in electron charge, *e*) of 7A.

| **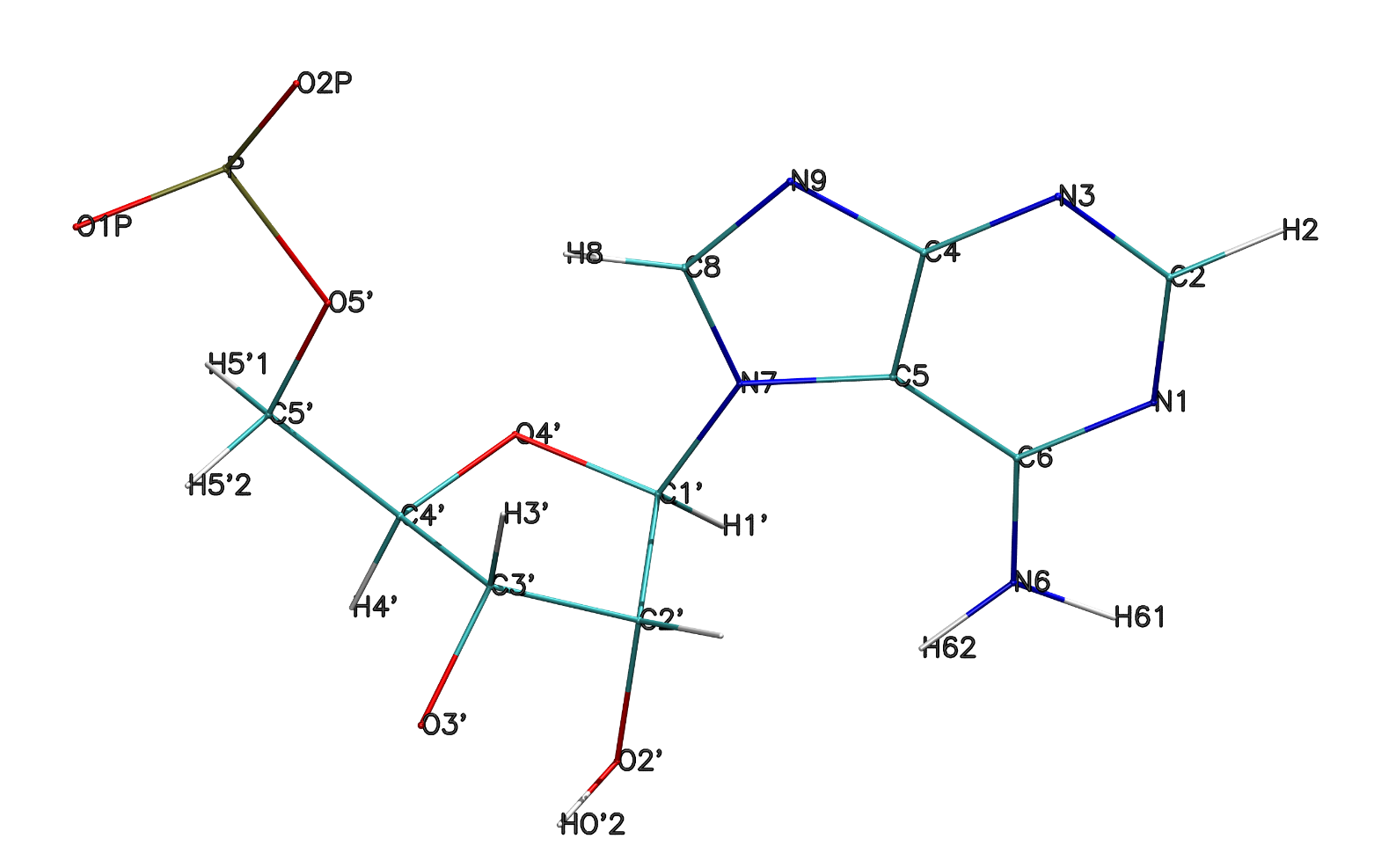** | | | | | |
| --- | --- | --- | --- | --- | --- |
| **Atom Name** | **Atom Type** | **Charge** | **Atom Name** | **Atom Type** | **Charge** |
| P | P | 1.1662 | C6 | CA | 0.969188 |
| O1P | O2 | -0.776 | N6 | N2 | -1.058492 |
| O2P | O2 | -0.776 | H61 | H | 0.447485 |
| O5′ | OS | -0.4989 | H62 | H | 0.447485 |
| C5′ | CI | 0.0558 | N1 | NC | -0.833341 |
| H5′1 | H1 | 0.0679 | C2 | CQ | 0.617045 |
| H5′2 | H1 | 0.0679 | H2 | H5 | 0.051631 |
| C4′ | CT | 0.1065 | N3 | NC | -0.806062 |
| H4′ | H1 | 0.1174 | C4 | CB | 0.931563 |
| O4′ | OS | -0.3548 | C3′ | CT | 0.2022 |
| C1′ | CT | -0.148455 | H3′ | H1 | 0.0615 |
| H1′ | H2 | 0.201447 | C2′ | CT | 0.067 |
| N9 | NB | -0.680395 | H2′1 | H1 | 0.0972 |
| C8 | C1 | 0.194899 | O2′ | OH | -0.6139 |
| H8 | H5 | 0.154794 | HO′2 | HO | 0.4186 |
| N7 | N* | 0.307772 | O3′ | OS | -0.5246 |
| C5 | CB | -0.680564 |  |  |  |

**Table S4.** Atom names, types, and RESP charges (in electron charge, *e*) of 7A^L^.

| **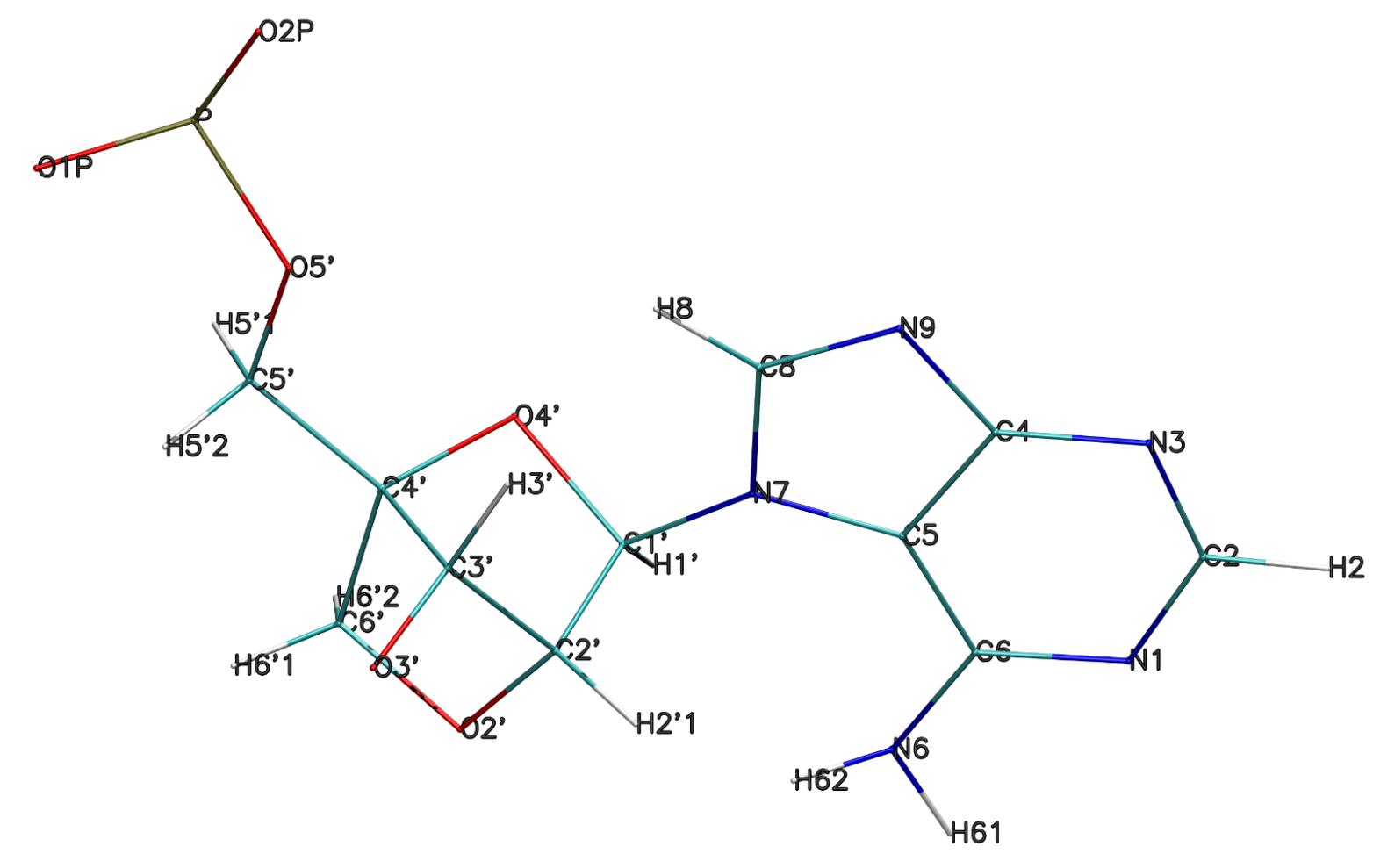** | | | | | |
| --- | --- | --- | --- | --- | --- |
| **Atom Name** | **Atom Type** | **Charge** | **Atom Name** | **Atom Type** | **Charge** |
| P | P | 1.279175 | N6 | N2 | -0.908711 |
| O1P | O2 | -0.810467 | H61 | H | 0.383294 |
| O2P | O2 | -0.810467 | H62 | H | 0.383294 |
| O5′ | OS | -0.533097 | N1 | NC | -0.826693 |
| C5′ | CI | 0.103509 | C2 | CQ | 0.626177 |
| H5′1 | H1 | 0.052405 | H2 | H5 | 0.059195 |
| H5′2 | H1 | 0.052405 | N3 | NC | -0.825942 |
| C4′ | CT | 0.25733 | C4 | CB | 0.994335 |
| O4′ | OS | -0.411377 | C3′ | CT | 0.253446 |
| C1′ | CT | 0.271117 | H3′ | H1 | 0.040572 |
| H1′ | H2 | 0.104505 | C2′ | CT | -0.065876 |
| N9 | NB | -0.705627 | H2′1 | H1 | 0.17102 |
| C8 | C7 | 0.315287 | O2′ | OS | -0.369899 |
| H8 | H5 | 0.112636 | C6′ | CT | -0.007598 |
| N7 | N* | -0.158116 | H6′1 | H1 | 0.093514 |
| C5 | CB | -0.530033 | H6′2 | H1 | 0.093514 |
| C6 | CA | 0.855988 | O3′ | OS | -0.538815 |

**Table S5.** Details of each RNA system studied computationally.

| **System Name** | **Duplexes (5′-3′)** | **Initial**  **Structures^a^** | **Simulation**  **Time (µs)** | **Water/Na^+^/Cl^-^**  **(# of atoms)** |
| --- | --- | --- | --- | --- |
| A-U | 5′ UCAG**A**CAGU  3′ AGUC**U**GUCA | syn-syn  syn-anti  anti-syn  anti-anti | 4  4  4  4 | 9000/29/13 |
| 7A-U | 5′ UCAG**7A**CAGU  3′AGUC **U** GUCA | syn-syn  syn-anti  anti-syn  anti-anti | 4  4  4  4 | 9000/29/13 |
| 7A^L^-U | 5′ UCAG**7A^L^**CAGU  3′ AGUC **U** GUCA | syn-syn  syn-anti  anti-syn  anti-anti | 4  4  4  4 | 9000/29/13 |
| A-C | 5′ UCAG**A**CAGU  3′ AGUC**C**GUCA | syn-syn  syn-anti  anti-syn  anti-anti | 4  4  4  4 | 9000/29/13 |
| 7A-C | 5′ UCAG**7A**CAGU  3′AGUC **C** GUCA | syn-syn  syn-anti  anti-syn  anti-anti | 4  4  4  4 | 9000/29/13 |
| 7A^L^-C | 5′ UCAG**7A^L^**CAGU  3′ AGUC **C** GUCA | syn-syn  syn-anti  anti-syn  anti-anti | 4  4  4  4 | 9000/29/13 |
| A-A | 5′ UCAG**A**CAGU  3′ AGUC**A**GUCA | syn-syn  syn-anti  anti-syn  anti-anti | 4  4  4  4 | 9000/29/13 |
| 7A-A | 5′ UCAG**7A**CAGU  3′AGUC **A** GUCA | syn-syn  syn-anti  anti-syn  anti-anti | 10.7  4  4  4 | 9000/29/13 |
| 7A^L^-A | 5′ UCAG**7A^L^**CAGU  3′ AGUC **A** GUCA | syn-syn  syn-anti  anti-syn  anti-anti | 4  4  4  4 | 9000/29/13 |
| A-G | 5′ UCAG**A**CAGU  3′ AGUC**G**GUCA | syn-syn  syn-anti  anti-syn  anti-anti | 4  4  4  4 | 9000/29/13 |
| 7A-G | 5′ UCAG**7A**CAGU  3′AGUC **G** GUCA | syn-syn  syn-anti  anti-syn  anti-anti | 6.6  4  4  4 | 9000/29/13 |
| 7A^L^-G | 5′ UCAG**7A^L^**CAGU  3′AGUC **G** GUCA | syn-syn  syn-anti  anti-syn  anti-anti | 4  4  4  4 | 9000/29/13 |

^a^ Orientations of glycosidic bonds of the middle base pairs, which are mismatches except AU pair.

**Table S6.** Sample input files used in minimization, equilibration, and production runs.

| Minimization – Step 1  &cntrl  imin = 1,  maxcyc = 10000,  ncyc = 5000,  ntb = 1,  ntr = 1,  cut = 10.0,  pencut=-0.001, nmropt=0,  restraintmask=":1-18 & !@H=", restraint_wt=1.0,  / |
| --- |
| Minimization – Step 2  &cntrl  imin = 1,  maxcyc = 10000,  ncyc = 5000,  ntb = 1,  ntr = 0,  cut = 10.0,  pencut=-0.001, nmropt=0,  / |
| Equilibration – Step 1  &cntrl  imin = 0, irest = 0, ntx = 1,  ntb = 1, cut = 10.0, ntr = 0,  ntc = 2, ntf = 2,  tempi = 0.0, temp0 = 300.0, ntt = 3, gamma_ln = 1.0, ig=-1,  nstlim = 1000000, dt = 0.002,  ntpr = 10000, ntwx = 10000, ntwr = 10000,  pencut=-0.001, nmropt=0,  restraintmask=":1-18 & !@H=", restraint_wt=1.0,  / |
| Equilibration – Step 2  &cntrl  imin = 0, irest = 1, ntx = 5,  ntb = 2, cut = 10.0, ntr = 0, pres0 = 1.0, ntp = 1, taup = 2.0,  ntc = 2, ntf = 2,  tempi = 300.0, temp0 = 300.0, ntt = 3, gamma_ln = 1.0, ig=-1,  nstlim = 1000000, dt = 0.002,  ntpr = 10000, ntwx = 10000, ntwr = 10000,  pencut=-0.001, nmropt=0,  / |
| 40 ns Production run (explicit solvent)  &cntrl  imin=0,  ntx=5,irest=1,  ntpr=10000,ntwr=10000,ntwx=10000,  ntc=2,ntf=2,ntb=2,cut=10,  igb=0,ntr=0,  nstlim=20000000,nmropt=0,dt=0.002,nscm=10000,  ntt=3,gamma_ln=1,tempi=300,temp0=300,ig=-1,  ntp=1,taup=2.0, pres0=1, ioutfm=1,  / |

**Table S7.** Experimental and literature ^1^H NMR data for A, 7A and 7A^L^ (units in ppm).

|  | **H8** | **H2** | **H6** | **H1′** | **H2′** | **H3′** | **H4′** | **H5′/H5′′** | **2′OH/3′OH** | **5′OH** |
| --- | --- | --- | --- | --- | --- | --- | --- | --- | --- | --- |
| **A** | 8.33 (s) | 8.13 (s) | 7.28 (brs) | 5.87 (d) | 4.59 (td) | 4.14 (td) | 3.96 (q) | 3.67 (ddd)  3.54 (ddd) | 5.44 (d)  5.19 (d) | 5.45 (dd) |
| **A^a^** | 8.381 | 8.168 | 7.41 | 5.907 | 4.641 | 4.171 | 3.993 | 3.696  3.580 | 5.51  5.24 | 5.48 |
| **7A** | 8.51 (s) | 8.22 (s) | 6.95 (brs) | 5.81 (d) | 4.08 (q) | 4.12 (m) | 3.99 (q) | 3.70 (ddd)  3.64 (ddd) | 5.58 (d)  5.27 (d) | 5.31 (t) |
| **7A^b^** | 8.52 (s) | 8.22 (s) | 6.99 (s) | 5.82 (d) | 4.07 (q) | 4.12 (m) | 3.99 (q) | 3.68 (m)  3.99 (m) | 5.62 (d)  5.31 (d) | 5.36 (t) |
| **7A^L^** | 8.41 (s) | 8.22 (s) | 6.83 (s) | 6.14 (s) | 4.68 (s) | 4.03 (s) | ---- | 3.75 (brs) | -----  5.12 (brs) | 5.12 (brs) |

**^a^** Ref (5). **^b^** Ref (1). s: singlet, brs: broad singlet, d: doublet, q: quartet, dd: doublet of doublets, t: triplet, q: quartet, m: multiplet.

**Table S8.** Experimental coupling constants ^1^H-^1^H and ^13^C-^1^H (Hz), calculated pseudorotational parameters (P, v_S_ and S%) and population of S% conformers, derived from equation %S=100*J_1′2′_/(J_1′2′_+J_3′4′_) for A, 7A and 7A^L^.

| Nucleoside | J_1′2′_  [Hz] | J_2′3′_  [Hz] | J_3′4′_  [Hz] | C8-H1′  [Hz] | C4-H1′ [Hz]  C5-H1′ [Hz] | P_S_; v_S_; S% | %S=100*J_1′2′_/(J_1′2′_+J_3′4′_) |
| --- | --- | --- | --- | --- | --- | --- | --- |
| A | 6.20 | 4.95 | 3.10 | 4.07-4.13 | 2.1-2.5 | 141.7-169.7  31-39.7  66-74% | 67% |
| A^a^ | 6.018 | 5.040 | 3.009 | 4 | n.d. | 67% | 67% |
| 7A | 7.00 | 6.05 | 3.50 | 3.60-3.65 | 1.1-1.2 | 119-132.7  25.2-37.2  70-91% | 67% |
| 7A^b^ | 7.2 | 6.0 | 5.0 | n.d. | n.d. | n.d. | 59% |
| 7A^L^ | n.d. | n.d. | n.d. | 2.73-2.90 | 0.88-1.05 | C3′-*endo* | n.d. |

**^a^** Ref (4). **^b^** Ref (1).

**Table S9.** Structurally significant NOEs observed for the internal loop mismatch pairs.

| **System** | **Proton pair** | **NOE intensity** |
| --- | --- | --- |
| **7A-G** | 7A5-H2 to C6-H5 | weak |
|  | G14-H1′ to G14-H8 | weak |
|  | 7A5-H1′ to 7A-H8 | strong |
| **7A^L^-G** | 7A^L^5-H2 to C6-H5 | weak |
|  | G14-H1′ to G14-H8 | weak |
|  | 7A^L^5-H1′ to 7A^L^5-H8 | strong |
|  | 7A^L^5-H2 to G14-H1 | medium |
| **7A-A** | 7A5-H2 to C6-H5 | medium |
|  | A14-H2 to C6-H1′ | strong |
|  | A14-H2 to C15-H1′ | strong |
|  | A14-H1′ to A14-H8 | weak |
|  | 7A5-H1′ to 7A5-H8 | weak |
|  | 7A5-H2 to A14-H2 | no NOE |
| **7A^L^-A** | 7A^L^5-H2 to C6-H5 | medium |
|  | A14-H2 to C6-H1′ | strong |
|  | A14-H2 to C15-H1′ | strong |
|  | A14-H1′ to A14-H8 | weak |
|  | 7A^L^5-H1′ to 7A^L^5-H8 | weak |
|  | 7A^L^5-H2 to A14-H2 | no NOE |

**Table S10**. Chemical shifts (in *ppm*) of aromatic and anomeric protons in duplexes containing 1×1 7A:G and 1×1 7A^L^:G mismatches.

| Residue | Chemical shifts of the **7A** duplex | | | Chemical shifts of the **7A^L^** duplex | | | Chemical shift differences between  the **7A** and **7A^L^** duplexes  (Absolute values) | | |
| --- | --- | --- | --- | --- | --- | --- | --- | --- | --- |
|  | H1′ | H6/H8 | H2/H5 | H1′ | H6/H8 | H2/H5 | H1′ | H6/H8 | H2/H5 |
| U1 | 5.470 | 8.025 | 5.801 | 5.487 | 8.036 | 5.811 | 0.017 | 0.011 | 0.010 |
| C2 | 5.656 | 8.014 | 5.824 | 5.666 | 8.020 | 5.827 | 0.010 | 0.006 | 0.003 |
| A3 | 5.899 | 8.040 | 6.921 | 5.895 | 8.032 | 6.842 | 0.004 | 0.008 | 0.079 |
| G4 | 5.591 | 7.233 | --- | 5.703 | 7.388 | --- | 0.112 | 0.155 | --- |
| **7A^(L)^**5 | 5.945 | 8.248 | 8.169 | 6.036 | 8.211 | 8.169 | 0.091 | 0.037 | 0.000 |
| C6 | 5.374 | 7.198 | 5.083 | 5.445 | 7.192 | 4.920 | 0.071 | 0.006 | 0.163 |
| A7 | 5.923 | 8.013 | 7.069 | 5.936 | 8.030 | 7.082 | 0.013 | 0.017 | 0.013 |
| G8 | 5.543 | 7.123 | --- | 5.556 | 7.138 | --- | 0.013 | 0.015 | --- |
| U9 | 5.755 | 7.556 | 5.125 | 5.754 | 7.560 | 5.131 | 0.001 | 0.004 | 0.006 |
| A10 | 5.666 | 8.303 | 7.939 | 5.684 | 8.311 | 7.947 | 0.018 | 0.008 | 0.008 |
| C11 | 5.464 | 7.683 | 5.256 | 5.466 | 7.689 | 5.255 | 0.002 | 0.006 | 0.001 |
| U12 | 5.556 | 7.873 | 5.388 | 5.563 | 7.888 | 5.381 | 0.007 | 0.015 | 0.007 |
| G13 | 5.752 | 7.651 | --- | 5.776 | 7.667 | --- | 0.024 | 0.016 | --- |
| **G14** | 5.672 | 7.562 | --- | 5.637 | 7.429 | --- | 0.035 | 0.133 | --- |
| C15 | 5.532 | 7.467 | 5.233 | 5.519 | 7.469 | 5.198 | 0.013 | 0.002 | 0.035 |
| U16 | 5.458 | 7.865 | 5.341 | 5.447 | 7.873 | 5.373 | 0.011 | 0.008 | 0.032 |
| G17 | 5.694 | 7.630 | --- | 5.683 | 7.617 | --- | 0.011 | 0.013 | --- |
| A18 | 5.973 | 7.824 | 7.832 | 5.978 | 7.821 | 7.844 | 0.005 | 0.003 | 0.012 |

**Table S11**. Chemical shifts (in *ppm*) of aromatic and anomeric protons in duplexes containing 1×1 7A:A and 1×1 7A^L^:A mismatches.

| Residue | Chemical shifts of the **7A** duplex | | | Chemical shifts of the **7A^L^** duplex | | | Chemical shift differences between  the **7A** and **7A^L^** duplexes  (Absolute values) | | |
| --- | --- | --- | --- | --- | --- | --- | --- | --- | --- |
|  | H1′ | H6/H8 | H2/H5 | H1′ | H6/H8 | H2/H5 | H1′ | H6/H8 | H2/H5 |
| U1 | 5.447 | 8.006 | 5.780 | 5.419 | 7.989 | 5.764 | 0.028 | 0.017 | 0.016 |
| C2 | 5.635 | 7.991 | 5.785 | 5.634 | 7.979 | 5.766 | 0.001 | 0.012 | 0.019 |
| A3 | 5.903 | 7.984 | 6.963 | 5.895 | 7.952 | 6.953 | 0.008 | 0.032 | 0.010 |
| G4 | 5.258 | 6.710 | --- | 5.357 | 6.752 | --- | 0.099 | 0.042 | --- |
| **7A^(L)^**5 | 5.811 | 8.335 | 8.100 | 5.696 | 7.741 | 8.138 | 0.115 | 0.594 | 0.038 |
| C6 | 5.537 | 7.471 | 5.050 | 5.497 | 7.617 | 5.005 | 0.040 | 0.146 | 0.045 |
| A7 | 5.829 | 7.962 | 7.090 | 5.806 | 7.939 | 7.079 | 0.023 | 0.023 | 0.011 |
| G8 | 5.559 | 7.150 | --- | 5.549 | 7.139 | --- | 0.010 | 0.011 | --- |
| U9 | 5.737 | 7.545 | 5.115 | 5.734 | 7.542 | 5.111 | 0.003 | 0.003 | 0.004 |
| A10 | 5.619 | 8.274 | 7.900 | 5.586 | 8.262 | 7.885 | 0.033 | 0.012 | 0.015 |
| C11 | 5.460 | 7.665 | 5.234 | 5.460 | 7.664 | 5.229 | 0.000 | 0.001 | 0.005 |
| U12 | 5.571 | 7.864 | 5.389 | 5.574 | 7.866 | 5.389 | 0.003 | 0.002 | 0.000 |
| G13 | 5.807 | 7.766 | --- | 5.811 | 7.763 | --- | 0.004 | 0.003 | --- |
| **A14** | 5.983 | 7.712 | 7.702 | 6.001 | 7.716 | 7.715 | 0.018 | 0.004 | 0.013 |
| C15 | 5.229 | 6.970 | 5.285 | 5.254 | 6.909 | 5.276 | 0.025 | 0.061 | 0.009 |
| U16 | 5.532 | 7.786 | 5.232 | 5.551 | 7.806 | 5.215 | 0.019 | 0.020 | 0.017 |
| G17 | 5.717 | 7.635 | --- | 5.717 | 7.644 | --- | 0.000 | 0.009 | --- |
| A18 | 5.969 | 7.812 | 7.813 | 5.971 | 7.809 | 7.798 | 0.002 | 0.003 | 0.015 |

**Table S12**. Binding free energies (kcal/mol), % fractions, χ torsions, and hydrogen bonds observed in clusters of system A-U (**Table S5**). Values next to ± display the standard deviations.

| **Case^a^** | **Cluster** | **% Fraction^b^** | **ΔG_MM/3D-RISM_**  **(kcal/mol)** | **ΔG_NMODE_**  **(kcal/mol)** | **ΔG_MM/3D-RISM_ - ΔG_NMODE_**  **(kcal/mol)** | **χ_5_-χ_14_^c^** | **Hydrogen**  **Bond^d^** |
| --- | --- | --- | --- | --- | --- | --- | --- |
| 5′-AG**A**CA-3′  3′-UC**U**GU-5′ | cluster_3 | 2.3 | -24.13 ± 3.24 | -25.99 ± 0.98 | 1.86 ± 3.38 | anti-anti | 1.98 |
|  | cluster_0 | 19.5 | -23.48 ± 3.31 | -25.92 ± 1.33 | 2.44 ± 3.57 | anti-anti | 2.00 |
|  | cluster_6 | 1.6 | -23.06 ± 3.36 | -25.74 ± 1.35 | 2.67 ± 3.62 | anti-anti | 1.99 |
|  | cluster_5 | 1.1 | -23.35 ± 3.59 | -26.06 ± 0.96 | 2.70 ± 3.72 | anti-anti | 1.86 |
|  | cluster_1 | 10.8 | -22.84 ± 3.18 | -26.00 ± 0.87 | 3.16 ± 3.29 | anti-anti | 1.97 |
|  | cluster_4 | 2.2 | -22.14 ± 3.34 | -25.65 ± 1.20 | 3.51 ± 3.55 | anti-anti | 2.00 |
|  | cluster_2 | 4.8 | -22.38 ± 3.21 | -26.04 ± 0.97 | 3.66 ± 3.35 | anti-anti | 1.93 |
|  | cluster_90 | 1.7 | -22.34 ± 3.22 | -26.15 ± 1.23 | 3.81 ± 3.44 | syn-anti | 1.98 |
|  | cluster_10 | 3.5 | -22.00 ± 3.29 | -25.97 ± 0.93 | 3.97 ± 3.42 | anti-anti | 1.98 |
|  | cluster_7 | 1.9 | -21.61 ± 2.99 | -26.38 ± 0.81 | 4.77 ± 3.10 | anti-anti | 1.89 |
|  | cluster_75 | 2.3 | -17.13 ± 3.31 | -26.01 ± 1.57 | 8.89 ± 3.66 | syn-anti | 0.99 |

^a^ System name (**Table S5**). ^b^ Percentage of structures observed in the combined trajectory representing the cluster. ^c^ Chi torsions of the middle basepairs highlighted in bold. ^d^ Total number of hydrogen bonds observed in middle basepairs.

**Table S13**. Binding free energies (kcal/mol), % fractions, χ torsions, and hydrogen bonds observed in clusters of system 7A-U (**Table S5**). Values next to ± display the standard deviations.

| **Case^a^** | **Cluster** | **% Fraction^b^** | **ΔG_MM/3D-RISM_**  **(kcal/mol)** | **ΔG_NMODE_**  **(kcal/mol)** | **ΔG_MM/3D-RISM_ - ΔG_NMODE_**  **(kcal/mol)** | **χ_5_-χ_14_^c^** | **Hydrogen**  **Bond^d^** |
| --- | --- | --- | --- | --- | --- | --- | --- |
| 5′-AG**7A**CA-3′  3′-UC **U** GU-5′ | cluster_4 | 1.8 | -20.83 ± 3.31 | -26.26 ± 0.86 | 5.43 ± 3.42 | anti-anti | 1.16 |
|  | cluster_2 | 2.1 | -20.12 ± 3.45 | -25.56 ± 1.56 | 5.44 ± 3.79 | anti-anti | 0.96 |
|  | cluster_146 | 4.4 | -19.94 ± 3.12 | -25.45 ± 0.87 | 5.51 ± 3.23 | syn-anti | 0.99 |
|  | cluster_157 | 1.7 | -20.25 ± 3.31 | -25.78 ± 1.12 | 5.54 ± 3.50 | syn-anti | 0.99 |
|  | cluster_150 | 2.6 | -19.76 ± 3.26 | -25.44 ± 1.11 | 5.68 ± 3.45 | syn-anti | 0.98 |
|  | cluster_144 | 9.5 | -19.84 ± 3.40 | -25.52 ± 1.46 | 5.69 ± 3.70 | syn-anti | 0.99 |
|  | cluster_145 | 1.3 | -19.85 ± 3.23 | -25.89 ± 1.12 | 6.04 ± 3.42 | syn-anti | 1.00 |
|  | cluster_148 | 6.6 | -19.58 ± 3.40 | -25.80 ± 1.14 | 6.22 ± 3.58 | syn-anti | 0.99 |
|  | cluster_0 | 6.4 | -19.66 ± 3.21 | -26.20 ± 1.19 | 6.54 ± 3.43 | anti-anti | 0.96 |
|  | cluster_5 | 2.7 | -17.98 ± 2.99 | -25.01 ± 1.03 | 7.03 ± 3.17 | anti-anti | 0.00 |
|  | cluster_158 | 1.9 | -18.68 ± 3.42 | -25.75 ± 0.99 | 7.07 ± 3.56 | syn-anti | 0.98 |
|  | cluster_147 | 1.6 | -17.52 ± 3.27 | -25.44 ± 1.56 | 7.92 ± 3.62 | syn-anti | 0.91 |
|  | cluster_8 | 1.3 | -17.26 ± 3.02 | -25.23 ± 1.06 | 7.97 ± 3.21 | anti-anti | 0.00 |
|  | cluster_6 | 1.4 | -16.54 ± 3.10 | -25.07 ± 1.04 | 8.52 ± 3.27 | anti-anti | 0.00 |
|  | cluster_3 | 2.5 | -15.53 ± 2.99 | -25.27 ± 0.90 | 9.73 ± 3.12 | anti-anti | 0.00 |

^a^ System name (**Table S5**). ^b^ Percentage of structures observed in the combined trajectory representing the cluster. ^c^ Chi torsions of the middle basepairs highlighted in bold. ^d^ Total number of hydrogen bonds observed in middle basepairs.

**Table S14**. Binding free energies (kcal/mol), % fractions, χ torsions, and hydrogen bonds observed in clusters of system 7A^L^-U (**Table S5**). Values next to ± display the standard deviations.

| **Case^a^** | **Cluster** | **% Fraction^b^** | **ΔG_MM/3D-RISM_**  **(kcal/mol)** | **ΔG_NMODE_**  **(kcal/mol)** | **ΔG_MM/3D-RISM_ - ΔG_NMODE_**  **(kcal/mol)** | **χ_5_-χ_14_^c^** | **Hydrogen**  **Bond^d^** |
| --- | --- | --- | --- | --- | --- | --- | --- |
| 5′-AG**7A^L^**CA-3′  3′-UC **U** GU-5′ | cluster_99 | 1.7 | -22.70 ± 3.37 | -25.55 ± 1.12 | 2.85 ± 3.55 | syn-anti | 1 |
|  | cluster_93 | 1.1 | -22.04 ± 3.25 | -25.70 ± 1.36 | 3.67 ± 3.52 | syn-anti | 0.99 |
|  | cluster_90 | 18.5 | -20.98 ± 3.36 | -25.56 ± 1.37 | 4.58 ± 3.63 | syn-anti | 0.99 |
|  | cluster_94 | 3.1 | -20.71 ± 3.48 | -25.43 ± 1.44 | 4.71 ± 3.77 | syn-anti | 0.98 |
|  | cluster_92 | 4 | -20.95 ± 3.29 | -25.72 ± 1.08 | 4.77 ± 3.46 | syn-anti | 0.99 |
|  | cluster_8 | 1.9 | -19.28 ± 3.04 | -24.31 ± 0.78 | 5.03 ± 3.14 | anti-anti | 0 |
|  | cluster_2 | 4.7 | -20.95 ± 3.26 | -26.11 ± 0.87 | 5.16 ± 3.38 | anti-anti | 1.03 |
|  | cluster_10 | 1.6 | -18.95 ± 2.94 | -24.14 ± 0.85 | 5.19 ± 3.06 | anti-anti | 0 |
|  | cluster_91 | 4 | -20.07 ± 3.25 | -25.64 ± 0.98 | 5.58 ± 3.40 | syn-anti | 0.98 |
|  | cluster_11 | 1.3 | -18.59 ± 2.96 | -24.36 ± 0.84 | 5.77 ± 3.08 | anti-anti | 0 |
|  | cluster_0 | 6.4 | -18.40 ± 2.97 | -24.38 ± 0.81 | 5.99 ± 3.08 | anti-anti | 0 |
|  | cluster_7 | 1.1 | -18.31 ± 3.10 | -24.33 ± 0.74 | 6.01 ± 3.19 | anti-anti | 0 |
|  | cluster_97 | 1.2 | -19.29 ± 3.28 | -25.93 ± 1.10 | 6.64 ± 3.46 | syn-anti | 0.97 |
|  | cluster_3 | 1 | -19.07 ± 3.24 | -26.32 ± 0.80 | 7.25 ± 3.34 | anti-anti | 0.93 |
|  | cluster_1 | 7 | -17.29 ± 2.98 | -24.55 ± 0.81 | 7.26 ± 3.09 | anti-anti | 0 |
|  | cluster_17 | 1.2 | -17.01 ± 2.76 | -24.53 ± 0.79 | 7.52 ± 2.87 | anti-anti | 0 |
|  | cluster_4 | 1.3 | -16.84 ± 3.01 | -24.49 ± 0.74 | 7.65 ± 3.10 | anti-anti | 0 |

^a^ System name (**Table S5**). ^b^ Percentage of structures observed in the combined trajectory representing the cluster. ^c^ Chi torsions of the middle basepairs highlighted in bold. ^d^ Total number of hydrogen bonds observed in middle basepairs.

**Table S15**. Binding free energies (kcal/mol), % fractions, χ torsions, and hydrogen bonds observed in clusters of system A-C (**Table S5**). Values next to ± display the standard deviations.

| **Case^a^** | **Cluster** | **% Fraction^b^** | **ΔG_MM/3D-RISM_**  **(kcal/mol)** | **ΔG_NMODE_**  **(kcal/mol)** | **ΔG_MM/3D-RISM_ - ΔG_NMODE_**  **(kcal/mol)** | **χ_5_-χ_14_^c^** | **Hydrogen**  **Bond^d^** |
| --- | --- | --- | --- | --- | --- | --- | --- |
| 5′-AG**A**CA-3′  3′-UC**C**GU-5′ | cluster_86 | 5.8 | -21.16 ± 3.39 | -25.51 ± 1.21 | 4.34 ± 3.60 | anti-syn | 1.00 |
|  | cluster_93 | 1.1 | -20.94 ± 3.22 | -25.61 ± 1.30 | 4.67 ± 3.47 | anti-syn | 0.99 |
|  | cluster_87 | 6.1 | -19.89 ± 3.39 | -24.68 ± 1.36 | 4.80 ± 3.65 | anti-syn | 1.00 |
|  | cluster_0 | 1.8 | -20.34 ± 3.07 | -25.20 ± 1.16 | 4.87 ± 3.28 | anti-anti | 1.00 |
|  | cluster_88 | 1.3 | -19.89 ± 3.27 | -25.00 ± 1.26 | 5.11 ± 3.50 | anti-syn | 1.00 |
|  | cluster_94 | 1.5 | -19.53 ± 3.40 | -24.83 ± 1.16 | 5.30 ± 3.59 | anti-syn | 0.99 |
|  | cluster_85 | 6.3 | -19.61 ± 3.42 | -25.22 ± 1.43 | 5.61 ± 3.71 | anti-syn | 1.00 |
|  | cluster_8 | 1.3 | -19.64 ± 3.14 | -25.39 ± 1.37 | 5.75 ± 3.42 | anti-anti | 1.00 |
|  | cluster_11 | 3.3 | -19.38 ± 3.30 | -25.16 ± 1.11 | 5.77 ± 3.49 | anti-anti | 1.00 |
|  | cluster_6 | 3.1 | -18.98 ± 3.09 | -25.34 ± 1.15 | 6.36 ± 3.30 | anti-anti | 1.00 |
|  | cluster_1 | 2.6 | -19.47 ± 3.03 | -25.93 ± 0.75 | 6.46 ± 3.12 | anti-anti | 1.00 |
|  | cluster_21 | 1.4 | -18.52 ± 3.10 | -24.99 ± 1.16 | 6.47 ± 3.31 | anti-anti | 1.00 |
|  | cluster_92 | 1.8 | -18.54 ± 3.63 | -25.14 ± 1.31 | 6.60 ± 3.86 | anti-syn | 1.00 |
|  | cluster_89 | 2.5 | -18.22 ± 3.50 | -24.84 ± 1.46 | 6.61 ± 3.79 | anti-syn | 0.99 |
|  | cluster_10 | 1.1 | -18.95 ± 2.99 | -25.83 ± 0.69 | 6.88 ± 3.07 | anti-anti | 0.99 |
|  | cluster_20 | 1.2 | -17.71 ± 3.28 | -25.21 ± 1.12 | 7.50 ± 3.47 | anti-anti | 1.00 |
|  | cluster_90 | 1.3 | -17.89 ± 3.36 | -25.40 ± 1.41 | 7.50 ± 3.64 | anti-syn | 1.00 |

^a^ System name (**Table S5**). ^b^ Percentage of structures observed in the combined trajectory representing the cluster. ^c^ Chi torsions of the middle basepairs highlighted in bold. ^d^ Total number of hydrogen bonds observed in middle basepairs.

**Table S16**. Binding free energies (kcal/mol), % fractions, χ torsions, and hydrogen bonds observed in clusters of system 7A-C (**Table S5**). Values next to ± display the standard deviations.

| **Case^a^** | **Cluster** | **% Fraction^b^** | **ΔG_MM/3D-RISM_**  **(kcal/mol)** | **ΔG_NMODE_**  **(kcal/mol)** | **ΔG_MM/3D-RISM_ - ΔG_NMODE_**  **(kcal/mol)** | **χ_5_-χ_14_^c^** | **Hydrogen**  **Bond^d^** |
| --- | --- | --- | --- | --- | --- | --- | --- |
| 5′-AG**7A**CA-3′  3′-UC **C** GU-5′ | cluster_149 | 1 | -18.20 ± 3.24 | -23.71 ± 2.30 | 5.51 ± 3.97 | syn-anti | 0.97 |
|  | cluster_4 | 1.1 | -19.01 ± 3.11 | -25.75 ± 0.92 | 6.73 ± 3.25 | anti-anti | 0.99 |
|  | cluster_2 | 2.3 | -18.65 ± 3.14 | -25.45 ± 1.23 | 6.80 ± 3.37 | anti-anti | 0.99 |
|  | cluster_1 | 7.3 | -18.64 ± 3.10 | -25.45 ± 1.19 | 6.81 ± 3.32 | anti-anti | 0.99 |
|  | cluster_146 | 2.1 | -17.69 ± 3.18 | -24.75 ± 1.09 | 7.05 ± 3.37 | syn-anti | 1.00 |
|  | cluster_0 | 2.1 | -18.73 ± 3.02 | -25.88 ± 0.88 | 7.16 ± 3.14 | anti-anti | 1.00 |
|  | cluster_5 | 1.3 | -18.31 ± 3.26 | -25.48 ± 1.23 | 7.18 ± 3.48 | anti-anti | 0.97 |
|  | cluster_142 | 1.8 | -17.82 ± 3.03 | -25.16 ± 1.17 | 7.34 ± 3.25 | syn-anti | 0.95 |
|  | cluster_136 | 4.8 | -17.48 ± 3.13 | -25.20 ± 1.36 | 7.72 ± 3.41 | syn-anti | 1.00 |
|  | cluster_7 | 3.7 | -17.86 ± 3.18 | -25.59 ± 1.31 | 7.73 ± 3.44 | anti-anti | 0.99 |
|  | cluster_141 | 4.2 | -17.14 ± 3.09 | -25.06 ± 1.42 | 7.92 ± 3.40 | syn-anti | 1.00 |
|  | cluster_135 | 3 | -16.83 ± 3.07 | -24.90 ± 1.27 | 8.07 ± 3.32 | syn-anti | 0.99 |
|  | cluster_138 | 1.2 | -17.06 ± 3.44 | -25.18 ± 1.25 | 8.12 ± 3.66 | syn-anti | 1.00 |
|  | cluster_8 | 1.6 | -17.44 ± 3.09 | -25.80 ± 1.02 | 8.36 ± 3.25 | anti-anti | 0.99 |
|  | cluster_144 | 1.2 | -16.70 ± 3.24 | -25.08 ± 1.20 | 8.39 ± 3.46 | syn-anti | 1.00 |
|  | cluster_145 | 1.4 | -16.18 ± 3.26 | -24.99 ± 1.37 | 8.81 ± 3.54 | syn-anti | 0.97 |
|  | cluster_140 | 1.7 | -15.34 ± 3.30 | -25.06 ± 1.40 | 9.72 ± 3.59 | syn-anti | 1.00 |

^a^ System name (**Table S5**). ^b^ Percentage of structures observed in the combined trajectory representing the cluster. ^c^ Chi torsions of the middle base pairs highlighted in bold. ^d^ Total number of hydrogen bonds observed in middle base pairs.

**Table S17**. Binding free energies (kcal/mol), % fractions, χ torsions, and hydrogen bonds observed in clusters of system 7A^L^-C (**Table S5**). Values next to ± display the standard deviations.

| **Case^a^** | **Cluster** | **% Fraction^b^** | **ΔG_MM/3D-RISM_**  **(kcal/mol)** | **ΔG_NMODE_**  **(kcal/mol)** | **ΔG_MM/3D-RISM_ - ΔG_NMODE_**  **(kcal/mol)** | **χ_5_-χ_14_^c^** | **Hydrogen**  **Bond^d^** |
| --- | --- | --- | --- | --- | --- | --- | --- |
| 5′-AG**7A^L^**CA-3′  3′-UC **C** GU-5′ | cluster_7 | 1.6 | -20.33 ± 3.11 | -25.09 ± 0.86 | 4.76 ± 3.23 | anti-anti | 0.99 |
|  | cluster_2 | 1.4 | -19.01 ± 3.08 | -24.55 ± 1.32 | 5.54 ± 3.35 | anti-anti | 0.98 |
|  | cluster_252 | 1.5 | -19.91 ± 3.38 | -25.45 ± 1.10 | 5.55 ± 3.55 | syn-syn | 1 |
|  | cluster_238 | 2 | -19.92 ± 3.06 | -25.79 ± 1.03 | 5.87 ± 3.23 | syn-syn | 0.99 |
|  | cluster_169 | 1.3 | -18.86 ± 3.04 | -24.92 ± 1.28 | 6.06 ± 3.30 | syn-anti | 1 |
|  | cluster_92 | 1.4 | -18.50 ± 3.37 | -24.76 ± 1.12 | 6.25 ± 3.55 | anti-syn | 0.97 |
|  | cluster_1 | 1.6 | -18.19 ± 3.13 | -24.51 ± 1.06 | 6.32 ± 3.31 | anti-anti | 0.99 |
|  | cluster_170 | 3.4 | -18.63 ± 3.08 | -25.28 ± 1.45 | 6.65 ± 3.40 | syn-anti | 0.96 |
|  | cluster_171 | 3 | -18.44 ± 3.17 | -25.09 ± 1.19 | 6.65 ± 3.38 | syn-anti | 0.99 |
|  | cluster_172 | 2.9 | -18.19 ± 3.24 | -25.17 ± 1.12 | 6.98 ± 3.42 | syn-anti | 1 |
|  | cluster_249 | 4.7 | -18.41 ± 3.17 | -25.40 ± 0.95 | 6.99 ± 3.31 | syn-syn | 0.99 |
|  | cluster_3 | 2.2 | -17.44 ± 2.94 | -24.65 ± 0.87 | 7.21 ± 3.06 | anti-anti | 0 |
|  | cluster_173 | 1 | -17.79 ± 3.30 | -25.12 ± 1.14 | 7.34 ± 3.49 | syn-anti | 1 |
|  | cluster_5 | 1.2 | -17.52 ± 3.26 | -25.06 ± 0.94 | 7.53 ± 3.39 | anti-anti | 0.29 |
|  | cluster_4 | 1.3 | -16.83 ± 3.13 | -24.44 ± 1.28 | 7.61 ± 3.39 | anti-anti | 0.01 |
|  | cluster_0 | 2.8 | -16.35 ± 3.19 | -24.34 ± 1.08 | 7.99 ± 3.37 | anti-anti | 0 |
|  | cluster_10 | 1.4 | -16.33 ± 3.06 | -24.66 ± 0.94 | 8.34 ± 3.20 | anti-anti | 0 |

^a^ System name (**Table S5**). ^b^ Percentage of structures observed in the combined trajectory representing the cluster. ^c^ Chi torsions of the middle base pairs highlighted in bold. ^d^ Total number of hydrogen bonds observed in middle base pairs.

**Table S18**. Binding free energies (kcal/mol), % fractions, χ torsions, and hydrogen bonds observed in clusters of system A-A (**Table S5**). Values next to ± display the standard deviations.

| **Case^a^** | **Cluster** | **% Fraction^b^** | **ΔG_MM/3D-RISM_**  **(kcal/mol)** | **ΔG_NMODE_**  **(kcal/mol)** | **ΔG_MM/3D-RISM_ - ΔG_NMODE_**  **(kcal/mol)** | **χ_5_-χ_14_^c^** | **Hydrogen**  **Bond^d^** |
| --- | --- | --- | --- | --- | --- | --- | --- |
| 5′-AG**A**CA-3′  3′-UC**A**GU-5′ | cluster_21 | 1 | -21.44 ± 3.62 | -24.92 ± 1.15 | 3.48 ± 3.80 | anti-anti | 0.96 |
|  | cluster_3 | 1.8 | -21.14 ± 3.27 | -25.33 ± 0.81 | 4.19 ± 3.37 | anti-anti | 1.00 |
|  | cluster_2 | 1.6 | -18.97 ± 3.58 | -25.12 ± 0.92 | 6.15 ± 3.69 | anti-anti | 0.78 |
|  | cluster_11 | 2.7 | -18.49 ± 3.32 | -24.89 ± 0.99 | 6.40 ± 3.46 | anti-anti | 0.00 |
|  | cluster_5 | 2.8 | -18.11 ± 3.58 | -24.82 ± 1.03 | 6.71 ± 3.73 | anti-anti | 0.00 |
|  | cluster_44 | 2 | -18.03 ± 3.36 | -24.78 ± 1.10 | 6.76 ± 3.54 | anti-anti | 0.03 |
|  | cluster_0 | 4.5 | -18.26 ± 3.29 | -25.21 ± 1.25 | 6.95 ± 3.52 | anti-anti | 0.00 |
|  | cluster_13 | 1.3 | -18.01 ± 3.31 | -25.07 ± 1.08 | 7.06 ± 3.48 | anti-anti | 0.00 |
|  | cluster_8 | 3.7 | -17.75 ± 3.26 | -25.04 ± 1.19 | 7.29 ± 3.47 | anti-anti | 0.00 |
|  | cluster_18 | 1.1 | -17.75 ± 3.13 | -25.12 ± 1.12 | 7.37 ± 3.32 | anti-anti | 0.00 |
|  | cluster_14 | 1.5 | -17.69 ± 3.27 | -25.30 ± 1.10 | 7.61 ± 3.45 | anti-anti | 0.00 |
|  | cluster_12 | 1.8 | -16.83 ± 3.25 | -24.74 ± 1.03 | 7.91 ± 3.41 | anti-anti | 0.00 |
|  | cluster_7 | 2 | -17.02 ± 3.07 | -25.05 ± 1.09 | 8.03 ± 3.26 | anti-anti | 0.00 |
|  | cluster_9 | 1.3 | -17.37 ± 3.07 | -25.43 ± 1.00 | 8.06 ± 3.22 | anti-anti | 0.00 |

^a^ System name (**Table S5**). ^b^ Percentage of structures observed in the combined trajectory representing the cluster. ^c^ Chi torsions of the middle base pairs highlighted in bold. ^d^ Total number of hydrogen bonds observed in middle base pairs.

**Table S19**. Binding free energies (kcal/mol), % fractions, χ torsions, and hydrogen bonds observed in clusters of system 7A-A (**Table S5**). Values next to ± display the standard deviations.

| **Case^a^** | **Cluster** | **% Fraction^b^** | **ΔG_MM/3D-RISM_**  **(kcal/mol)** | **ΔG_NMODE_**  **(kcal/mol)** | **ΔG_MM/3D-RISM_ - ΔG_NMODE_**  **(kcal/mol)** | **χ_5_-χ_14_^c^** | **Hydrogen**  **Bond^d^** |
| --- | --- | --- | --- | --- | --- | --- | --- |
| 5′-AG**7A**CA-3′  3′-UC **A** GU-5′ | cluster_1 | 3.8 | -24.42 ± 3.38 | -25.93 ± 0.86 | 1.50 ± 3.49 | anti-anti | 1.99 |
|  | cluster_3 | 2.1 | -24.11 ± 3.50 | -25.92 ± 0.79 | 1.81 ± 3.59 | anti-anti | 2.00 |
|  | cluster_2 | 9 | -23.99 ± 3.17 | -26.10 ± 0.74 | 2.11 ± 3.26 | anti-anti | 2.00 |
|  | cluster_0 | 11.5 | -23.15 ± 3.18 | -26.10 ± 0.70 | 2.94 ± 3.26 | anti-anti | 1.99 |
|  | cluster_103 | 1.9 | -20.57 ± 3.25 | -23.80 ± 1.97 | 3.23 ± 3.80 | syn-anti | 1.00 |
|  | cluster_6 | 1.3 | -22.42 ± 3.26 | -26.09 ± 0.73 | 3.66 ± 3.34 | anti-anti | 1.99 |
|  | cluster_98 | 1.6 | -20.66 ± 3.50 | -24.54 ± 1.87 | 3.89 ± 3.97 | syn-anti | 0.99 |
|  | cluster_105 | 1.9 | -19.58 ± 3.21 | -24.15 ± 2.01 | 4.57 ± 3.79 | syn-anti | 1.00 |
|  | cluster_106 | 1.2 | -18.23 ± 3.03 | -23.70 ± 1.90 | 5.47 ± 3.58 | syn-anti | 1.00 |
|  | cluster_203 | 1.1 | -16.76 ± 3.42 | -26.08 ± 0.92 | 9.32 ± 3.54 | syn-syn | 0.95 |
|  | cluster_182 | 1.1 | -14.97 ± 3.29 | -25.60 ± 1.29 | 10.63 ± 3.53 | syn-syn | 0.97 |
|  | cluster_204 | 1.8 | -14.99 ± 3.40 | -26.51 ± 1.26 | 11.52 ± 3.62 | syn-syn | 0.92 |

^a^ System name (**Table S5**). ^b^ Percentage of structures observed in the combined trajectory representing the cluster. ^c^ Chi torsions of the middle base pairs highlighted in bold. ^d^ Total number of hydrogen bonds observed in middle base pairs.

**Table S20**. Binding free energies (kcal/mol), % fractions, χ torsions, and hydrogen bonds observed in clusters of system 7A^L^-A (**Table S5**). Values next to ± display the standard deviations.

| **Case^a^** | **Cluster** | **% Fraction^b^** | **ΔG_MM/3D-RISM_**  **(kcal/mol)** | **ΔG_NMODE_**  **(kcal/mol)** | **ΔG_MM/3D-RISM_ - ΔG_NMODE_**  **(kcal/mol)** | **χ_5_-χ_14_^c^** | **Hydrogen**  **Bond^d^** |
| --- | --- | --- | --- | --- | --- | --- | --- |
| 5′-AG**7A^L^**CA-3′  3′-UC **A** GU-5′ | cluster_16 | 1.2 | -23.26 ± 3.48 | -24.69 ± 0.98 | 1.44 ± 3.62 | anti-anti | 1.99 |
|  | cluster_4 | 2.2 | -23.52 ± 3.48 | -25.04 ± 0.93 | 1.52 ± 3.61 | anti-anti | 1.98 |
|  | cluster_0 | 3.1 | -22.57 ± 3.21 | -24.96 ± 0.76 | 2.39 ± 3.30 | anti-anti | 1.98 |
|  | cluster_169 | 3.8 | -20.16 ± 3.26 | -25.62 ± 1.26 | 5.45 ± 3.50 | syn-anti | 1.00 |
|  | cluster_8 | 1 | -18.09 ± 3.31 | -23.76 ± 1.23 | 5.67 ± 3.53 | anti-anti | 0.00 |
|  | cluster_9 | 1.7 | -19.55 ± 3.46 | -25.29 ± 0.90 | 5.73 ± 3.58 | anti-anti | 0.00 |
|  | cluster_5 | 1.8 | -19.14 ± 3.25 | -25.12 ± 1.03 | 5.98 ± 3.40 | anti-anti | 0.00 |
|  | cluster_64 | 1.5 | -17.97 ± 3.10 | -24.07 ± 1.91 | 6.10 ± 3.64 | anti-syn | 0.00 |
|  | cluster_7 | 1.7 | -18.49 ± 3.30 | -24.87 ± 1.27 | 6.38 ± 3.54 | anti-anti | 0.00 |
|  | cluster_187 | 1 | -18.42 ± 3.19 | -24.97 ± 1.13 | 6.55 ± 3.39 | syn-anti | 0.00 |
|  | cluster_178 | 1.1 | -18.49 ± 3.13 | -25.04 ± 1.40 | 6.55 ± 3.43 | syn-anti | 0.00 |
|  | cluster_170 | 1.3 | -19.13 ± 3.18 | -25.72 ± 1.22 | 6.59 ± 3.41 | syn-anti | 1.00 |
|  | cluster_71 | 1.4 | -16.27 ± 3.05 | -23.29 ± 0.94 | 7.02 ± 3.20 | anti-syn | 0.00 |
|  | cluster_174 | 1.2 | -18.28 ± 3.21 | -25.37 ± 1.15 | 7.09 ± 3.41 | syn-anti | 1.00 |
|  | cluster_226 | 1.2 | -17.01 ± 3.53 | -24.88 ± 1.31 | 7.87 ± 3.76 | syn-syn | 0.98 |
|  | cluster_225 | 4.1 | -16.18 ± 3.47 | -24.97 ± 1.31 | 8.80 ± 3.71 | syn-syn | 0.99 |
|  | cluster_243 | 1 | -15.77 ± 3.49 | -24.89 ± 1.18 | 9.12 ± 3.69 | syn-syn | 0.93 |
|  | cluster_230 | 1.7 | -16.68 ± 3.20 | -26.17 ± 1.11 | 9.49 ± 3.39 | syn-syn | 0.99 |
|  | cluster_232 | 1.5 | -16.57 ± 3.15 | -26.23 ± 1.20 | 9.66 ± 3.37 | syn-syn | 0.94 |

^a^ System name (**Table S5**). ^b^ Percentage of structures observed in the combined trajectory representing the cluster. ^c^ Chi torsions of the middle base pairs highlighted in bold. ^d^ Total number of hydrogen bonds observed in middle base pairs.

**Table S21**. Binding free energies (kcal/mol), % fractions, χ torsions, and hydrogen bonds observed in clusters of system A-G (**Table S5**). Values next to ± display the standard deviations.

| **Case^a^** | **Cluster** | **% Fraction^b^** | **ΔG_MM/3D-RISM_**  **(kcal/mol)** | **ΔG_NMODE_**  **(kcal/mol)** | **ΔG_MM/3D-RISM_ - ΔG_NMODE_**  **(kcal/mol)** | **χ_5_-χ_14_^c^** | **Hydrogen**  **Bond^d^** |
| --- | --- | --- | --- | --- | --- | --- | --- |
| 5′-AG**A**CA-3′  3′-UC**G**GU-5′ | cluster_159 | 5.6 | -23.71 ± 3.14 | -26.52 ± 0.91 | 2.81 ± 3.27 | syn-anti | 1.99 |
|  | cluster_167 | 3.4 | -22.94 ± 3.30 | -26.68 ± 0.82 | 3.74 ± 3.40 | syn-anti | 2.00 |
|  | cluster_170 | 1.7 | -22.11 ± 3.44 | -26.34 ± 0.90 | 4.23 ± 3.56 | syn-anti | 1.92 |
|  | cluster_163 | 2.9 | -21.97 ± 3.27 | -26.61 ± 0.84 | 4.64 ± 3.37 | syn-anti | 1.99 |
|  | cluster_83 | 2.1 | -20.06 ± 3.15 | -25.34 ± 1.42 | 5.29 ± 3.46 | anti-syn | 1.00 |
|  | cluster_81 | 1.5 | -19.40 ± 3.07 | -25.30 ± 1.33 | 5.90 ± 3.35 | anti-syn | 1.00 |
|  | cluster_86 | 2 | -18.80 ± 2.86 | -25.12 ± 1.35 | 6.31 ± 3.16 | anti-syn | 1.00 |
|  | cluster_91 | 1.2 | -18.52 ± 3.17 | -25.29 ± 1.21 | 6.77 ± 3.39 | anti-syn | 1.00 |
|  | cluster_1 | 5.8 | -17.68 ± 3.05 | -25.36 ± 0.74 | 7.68 ± 3.14 | anti-anti | 0.00 |
|  | cluster_74 | 1.6 | -18.75 ± 3.02 | -26.45 ± 1.30 | 7.70 ± 3.28 | anti-syn | 1.00 |
|  | cluster_77 | 1.3 | -18.57 ± 2.97 | -26.34 ± 1.29 | 7.77 ± 3.24 | anti-syn | 1.00 |
|  | cluster_5 | 1.9 | -16.84 ± 3.34 | -25.32 ± 0.77 | 8.48 ± 3.43 | anti-anti | 0.00 |
|  | cluster_76 | 1 | -17.45 ± 3.17 | -26.13 ± 1.29 | 8.68 ± 3.42 | anti-syn | 1.00 |
|  | cluster_218 | 2.3 | -15.55 ± 3.54 | -25.44 ± 0.97 | 9.89 ± 3.67 | syn-syn | 1.00 |
|  | cluster_221 | 1.2 | -15.37 ± 3.47 | -25.72 ± 0.88 | 10.35 ± 3.58 | syn-syn | 1.00 |

^a^ System name (**Table S5**). ^b^ Percentage of structures observed in the combined trajectory representing the cluster. ^c^ Chi torsions of the middle base pairs highlighted in bold. ^d^ Total number of hydrogen bonds observed in middle base pairs.

**Table S22**. Binding free energies (kcal/mol), % fractions, χ torsions, and hydrogen bonds observed in clusters of system 7A-G (**Table S5**). Values next to ± display the standard deviations.

| **Case^a^** | **Cluster** | **% Fraction^b^** | **ΔG_MM/3D-RISM_**  **(kcal/mol)** | **ΔG_NMODE_**  **(kcal/mol)** | **ΔG_MM/3D-RISM_ - ΔG_NMODE_**  **(kcal/mol)** | **χ_5_-χ_14_^c^** | **Hydrogen**  **Bond^d^** |
| --- | --- | --- | --- | --- | --- | --- | --- |
| 5′-AG**7A**CA-3′  3′-UC **G** GU-5′ | cluster_170 | 1.2 | -24.39 ± 3.27 | -26.49 ± 0.57 | 2.10 ± 3.32 | syn-anti | 1.54 |
|  | cluster_169 | 5 | -23.92 ± 3.43 | -26.54 ± 0.85 | 2.63 ± 3.54 | syn-anti | 1.63 |
|  | cluster_168 | 3.8 | -23.64 ± 3.22 | -26.51 ± 0.80 | 2.88 ± 3.32 | syn-anti | 1.45 |
|  | cluster_172 | 1.6 | -23.21 ± 3.34 | -26.43 ± 0.99 | 3.22 ± 3.49 | syn-anti | 1.82 |
|  | cluster_2 | 2.2 | -22.37 ± 3.66 | -26.51 ± 0.93 | 4.13 ± 3.77 | anti-anti | 1.00 |
|  | cluster_0 | 6 | -21.71 ± 3.51 | -26.77 ± 0.91 | 5.06 ± 3.63 | anti-anti | 1.00 |
|  | cluster_9 | 1.1 | -20.00 ± 3.28 | -26.65 ± 0.83 | 6.66 ± 3.39 | anti-anti | 1.00 |
|  | cluster_67 | 1.3 | -16.60 ± 3.06 | -23.62 ± 1.46 | 7.02 ± 3.39 | anti-syn | 0.00 |
|  | cluster_79 | 1.4 | -17.23 ± 2.95 | -25.39 ± 1.38 | 8.17 ± 3.26 | anti-syn | 0.00 |

^a^ System name (**Table S5**). ^b^ Percentage of structures observed in the combined trajectory representing the cluster. ^c^ Chi torsions of the middle base pairs highlighted in bold. ^d^ Total number of hydrogen bonds observed in middle base pairs.

**Table S23**. Binding free energies (kcal/mol), % fractions, χ torsions, and hydrogen bonds observed in clusters of system 7A^L^-G (**Table S5**). Values next to ± display the standard deviations.

| **Case^a^** | **Cluster** | **% Fraction^b^** | **ΔG_MM/3D-RISM_**  **(kcal/mol)** | **ΔG_NMODE_**  **(kcal/mol)** | **ΔG_MM/3D-RISM_ – ΔG_NMODE_**  **(kcal/mol)** | **χ_5_-χ_14_^c^** | **Hydrogen**  **Bond^d^** |
| --- | --- | --- | --- | --- | --- | --- | --- |
| 5′-AG**7A^L^**CA-3′  3′-UC **G** GU-5′ | cluster_122 | 7.9 | -25.03 ± 3.40 | -26.25 ± 0.93 | 1.22 ± 3.53 | syn-anti | 1.75 |
|  | cluster_124 | 4.4 | -25.07 ± 3.35 | -26.42 ± 0.78 | 1.35 ± 3.44 | syn-anti | 1.74 |
|  | cluster_125 | 2.9 | -24.14 ± 3.36 | -26.22 ± 0.83 | 2.08 ± 3.46 | syn-anti | 1.53 |
|  | cluster_1 | 4.4 | -23.03 ± 3.62 | -25.30 ± 0.87 | 2.27 ± 3.73 | anti-anti | 1.00 |
|  | cluster_123 | 1.6 | -23.83 ± 3.27 | -26.34 ± 0.85 | 2.51 ± 3.38 | syn-anti | 1.77 |
|  | cluster_0 | 4.3 | -23.07 ± 3.58 | -25.61 ± 0.89 | 2.54 ± 3.69 | anti-anti | 1.00 |
|  | cluster_5 | 2.5 | -22.42 ± 3.59 | -25.36 ± 0.71 | 2.94 ± 3.66 | anti-anti | 1.00 |
|  | cluster_2 | 1.3 | -22.49 ± 3.50 | -25.49 ± 0.92 | 3.00 ± 3.62 | anti-anti | 1.00 |
|  | cluster_3 | 1.7 | -21.38 ± 3.47 | -25.39 ± 0.79 | 4.01 ± 3.56 | anti-anti | 1.00 |
|  | cluster_4 | 1 | -20.99 ± 3.53 | -25.84 ± 1.47 | 4.85 ± 3.82 | anti-anti | 1.00 |
|  | cluster_58 | 1.1 | -17.95 ± 3.00 | -23.69 ± 1.37 | 5.74 ± 3.30 | anti-syn | 0.00 |
|  | cluster_59 | 1.3 | -17.30 ± 3.05 | -23.66 ± 1.40 | 6.36 ± 3.36 | anti-syn | 0.00 |
|  | cluster_48 | 3 | -17.73 ± 3.07 | -24.98 ± 1.53 | 7.25 ± 3.43 | anti-syn | 0.00 |
|  | cluster_52 | 1.1 | -17.16 ± 2.88 | -25.10 ± 0.96 | 7.94 ± 3.04 | anti-syn | 0.00 |
|  | cluster_51 | 2.1 | -16.92 ± 3.10 | -25.12 ± 1.01 | 8.20 ± 3.25 | anti-syn | 0.00 |

^a^ System name (**Table S5**). ^b^ Percentage of structures observed in the combined trajectory representing the cluster. ^c^ Chi torsions of the middle base pairs highlighted in bold. ^d^ Total number of hydrogen bonds observed in middle base pairs.

**Table S24**. Comparison of structurally significant NOEs to predicted distances for each cluster in 7A-A (see also **Tables S9** and **S19**). Values next to ± display the standard deviations.

| **Case^a^** | **Cluster** | **ΔG^b^** | **%**  **Frac.^c^** | **χ_5_-χ_14_^d^** | **H-Bond^e^** | **7A5@H2- C6@H5** | | **A14@H2- C6@H1′** | | **A14@H2- C15@H1′** | | **A14@H1′- A14@H8** | | **7A5@H1′- 7A5@H8** | | **7A5@H2- A14@H2** | |
| --- | --- | --- | --- | --- | --- | --- | --- | --- | --- | --- | --- | --- | --- | --- | --- | --- | --- |
|  |  |  |  |  |  | **Predicted**  **Distance**  **(Å)** | **NOE**  **Intensity** | **Predicted**  **Distance**  **(Å)** | **NOE**  **Intensity** | **Predicted**  **Distance**  **(Å)** | **NOE**  **Intensity** | **Predicted**  **Distance**  **(Å)** | **NOE**  **Intensity** | **Predicted**  **Distance**  **(Å)** | **NOE**  **Intensity** | **Predicted**  **Distance**  **(Å)** | **NOE**  **Intensity** |
| **7A-A** | cluster_1 | 1.50 | 3.8 | *anti*-*anti* | 1.99 | 4.3 ± 0.4 | Medium | 3.1 ± 0.4 | Strong | 3.3 ± 0.4 | Strong | 3.7 ± 0.1 | Weak | 3.8 ± 0.1 | Weak | 6.8 ± 0.3 | No NOE |
|  | cluster_3 | 1.81 | 2.1 | *anti*-*anti* | 2.00 | 4.6 ± 0.4 |  | 3.1 ± 0.4 |  | 3.3 ± 0.4 |  | 3.7 ± 0.1 |  | 3.8 ± 0.1 |  | 6.8 ± 0.3 |  |
|  | cluster_2 | 2.11 | 9 | *anti*-*anti* | 2.00 | 4.6 ± 0.4 |  | 3.1 ± 0.4 |  | 3.4 ± 0.5 |  | 3.7 ± 0.1 |  | 3.8 ± 0.1 |  | 6.8 ± 0.3 |  |
|  | cluster_0 | 2.94 | 11.5 | *anti*-*anti* | 1.99 | 4.5 ± 0.4 |  | 3.2 ± 0.4 |  | 3.3 ± 0.4 |  | 3.8 ± 0.1 |  | 3.8 ± 0.1 |  | 6.8 ± 0.3 |  |
|  | cluster_103 | 3.23 | 1.9 | *syn*-*anti* | 1.00 | 4.4 ± 0.5 |  | 3.5 ± 0.5 |  | 3.6 ± 0.5 |  | 3.8 ± 0.1 |  | 2.6 ± 0.1 |  | 9.3 ± 0.3 |  |
|  | cluster_6 | 3.66 | 1.3 | *anti*-*anti* | 1.99 | 4.4 ± 0.5 |  | 3.3 ± 0.4 |  | 3.4 ± 0.5 |  | 3.7 ± 0.1 |  | 3.8 ± 0.1 |  | 6.8 ± 0.3 |  |
|  | cluster_98 | 3.89 | 1.6 | *syn*-*anti* | 0.99 | 5.4 ± 0.5 |  | 3.3 ± 0.5 |  | 3.4 ± 0.5 |  | 3.7 ± 0.1 |  | 2.6 ± 0.1 |  | 9.4 ± 0.3 |  |
|  | cluster_105 | 4.57 | 1.9 | *syn*-*anti* | 1.00 | 5.1 ± 0.5 |  | 3.5 ± 0.5 |  | 3.4 ± 0.5 |  | 3.8 ± 0.1 |  | 2.6 ± 0.1 |  | 9.5 ± 0.4 |  |
|  | cluster_106 | 5.47 | 1.2 | *syn*-*anti* | 1.00 | 4.3 ± 0.4 |  | 4.1 ± 0.7 |  | 3.6 ± 0.6 |  | 3.8 ± 0.1 |  | 2.5 ± 0.1 |  | 9.5 ± 0.4 |  |
|  | cluster_203 | 9.32 | 1.1 | *syn*-*syn* | 0.95 | 4.2 ± 0.4 |  | 9.0 ± 0.4 |  | 6.8 ± 0.6 |  | 2.5 ± 0.1 |  | 2.6 ± 0.1 |  | 6.4 ± 0.4 |  |
|  | cluster_182 | 10.63 | 1.1 | *syn*-*syn* | 0.97 | 4.0 ± 0.3 |  | 10.0 ± 0.4 |  | 7.9 ± 0.4 |  | 2.6 ± 0.1 |  | 2.5 ± 0.1 |  | 6.8 ± 0.3 |  |
|  | cluster_204 | 11.52 | 1.8 | *syn*-*syn* | 0.92 | 4.1 ± 0.4 |  | 10.1 ± 0.4 |  | 7.7 ± 0.4 |  | 2.6 ± 0.1 |  | 2.5 ± 0.1 |  | 6.7 ± 0.3 |  |

^a^ System name (**Table S5**). ^b^ In kcal/mol. ^c^ Percentage of structures observed in the combined trajectory representing the cluster. ^d^ Chi torsions of the middle mismatches. ^e^ Total number of hydrogen bonds observed in middle base pairs. For residue numbering, see **Figure 2**. Red highlighted sections display MD data not agreeing with NMR results.

**Table S25**. Comparison of structurally significant NOEs to predicted distances for each cluster in 7A^L^-A (see also **Tables S9** and **S20**). Values next to ± display the standard deviations.

| **Case^a^** | **Cluster** | **ΔG^b^** | **%**  **Frac.^c^** | **χ_5_-χ_14_^d^** | **H-Bond^e^** | **7A^L^5@H2- C6@H5** | | **A14@H2- C6@H1′** | | **A14@H2- C15@H1′** | | **A14@H1′- A14@H8** | | **7A^L^5@H1′- 7A5@H8** | | **7A^L^5@H2- A14@H2** | |
| --- | --- | --- | --- | --- | --- | --- | --- | --- | --- | --- | --- | --- | --- | --- | --- | --- | --- |
|  |  |  |  |  |  | **Predicted**  **Distance**  **(Å)** | **NOE**  **Intensity** | **Predicted**  **Distance**  **(Å)** | **NOE**  **Intensity** | **Predicted**  **Distance**  **(Å)** | **NOE**  **Intensity** | **Predicted**  **Distance**  **(Å)** | **NOE**  **Intensity** | **Predicted**  **Distance**  **(Å)** | **NOE**  **Intensity** | **Predicted**  **Distance**  **(Å)** | **NOE**  **Intensity** |
| **7A^L^-A** | cluster_16 | 1.44 | 1.2 | *anti*-*anti* | 1.99 | 4.0 ± 0.4 | Medium | 3.1 ± 0.4 | Strong | 3.7 ± 0.5 | Strong | 3.8 ± 0.1 | Weak | 3.8 ± 0.1 | Weak | 6.8 ± 0.3 | No NOE |
|  | cluster_4 | 1.52 | 2.2 | *anti*-*anti* | 1.98 | 3.8 ± 0.4 |  | 3.1 ± 0.4 |  | 3.5 ± 0.5 |  | 3.8 ± 0.1 |  | 3.8 ± 0.1 |  | 6.8 ± 0.3 |  |
|  | cluster_0 | 2.39 | 3.1 | *anti*-*anti* | 1.98 | 4.0 ± 0.4 |  | 3.2 ± 0.4 |  | 3.6 ± 0.5 |  | 3.8 ± 0.1 |  | 3.8 ± 0.1 |  | 6.8 ± 0.3 |  |
|  | cluster_169 | 5.45 | 3.8 | *syn*-*anti* | 1.00 | 4.2 ± 0.4 |  | 3.1 ± 0.4 |  | 3.3 ± 0.5 |  | 3.8 ± 0.1 |  | 2.6 ± 0.1 |  | 9.3 ± 0.4 |  |
|  | cluster_8 | 5.67 | 1 | *anti*-*anti* | 0.00 | 3.9 ± 0.4 |  | 5.9 ± 0.7 |  | 6.2 ± 0.5 |  | 3.8 ± 0.1 |  | 3.8 ± 0.1 |  | 2.3 ± 0.3 |  |
|  | cluster_9 | 5.73 | 1.7 | *anti*-*anti* | 0.00 | 4.9 ± 0.4 |  | 6.5 ± 0.5 |  | 6.4 ± 0.4 |  | 3.8 ± 0.1 |  | 3.7 ± 0.1 |  | 2.5 ± 0.3 |  |
|  | cluster_5 | 5.98 | 1.8 | *anti*-*anti* | 0.00 | 4.7 ± 0.4 |  | 6.8 ± 0.5 |  | 6.4 ± 0.4 |  | 3.8 ± 0.1 |  | 3.7 ± 0.1 |  | 2.4 ± 0.3 |  |
|  | cluster_64 | 6.10 | 1.5 | *anti*-*syn* | 0.00 | 5.0 ± 0.3 |  | 11.9 ± 0.4 |  | 11.0 ± 0.4 |  | 2.5 ± 0.1 |  | 3.8 ± 0.1 |  | 8.0 ± 0.3 |  |
|  | cluster_7 | 6.38 | 1.7 | *anti*-*anti* | 0.00 | 4.9 ± 0.4 |  | 6.2 ± 0.5 |  | 6.1 ± 0.5 |  | 3.8 ± 0.1 |  | 3.8 ± 0.1 |  | 2.3 ± 0.3 |  |
|  | cluster_187 | 6.55 | 1 | *syn*-*anti* | 0.00 | 4.2 ± 0.5 |  | 5.6 ± 0.7 |  | 6.0 ± 0.5 |  | 3.8 ± 0.1 |  | 2.6 ± 0.1 |  | 5.1 ± 0.6 |  |
|  | cluster_178 | 6.55 | 1.1 | *syn*-*anti* | 0.00 | 3.7 ± 0.4 |  | 5.3 ± 0.7 |  | 6.0 ± 0.5 |  | 3.8 ± 0.1 |  | 2.6 ± 0.1 |  | 3.9 ± 0.4 |  |
|  | cluster_170 | 6.59 | 1.3 | *syn*-*anti* | 1.00 | 3.9 ± 0.3 |  | 3.4 ± 0.4 |  | 3.3 ± 0.5 |  | 3.8 ± 0.1 |  | 2.6 ± 0.1 |  | 9.1 ± 0.4 |  |
|  | cluster_71 | 7.02 | 1.4 | *anti*-*syn* | 0.00 | 5.1 ± 0.4 |  | 11.2 ± 0.4 |  | 8.7 ± 0.5 |  | 2.5 ± 0.1 |  | 3.8 ± 0.1 |  | 7.1 ± 0.4 |  |
|  | cluster_174 | 7.09 | 1.2 | *syn*-*anti* | 1.00 | 4.0 ± 0.4 |  | 3.7 ± 0.4 |  | 3.1 ± 0.4 |  | 3.7 ± 0.1 |  | 2.6 ± 0.1 |  | 9.1 ± 0.4 |  |
|  | cluster_226 | 7.87 | 1.2 | *syn*-*syn* | 0.98 | 3.9 ± 0.3 |  | 9.8 ± 0.3 |  | 8.3 ± 0.4 |  | 2.7 ± 0.1 |  | 2.6 ± 0.1 |  | 7.0 ± 0.3 |  |
|  | cluster_225 | 8.80 | 4.1 | *syn*-*syn* | 0.99 | 3.9 ± 0.3 |  | 9.8 ± 0.3 |  | 8.0 ± 0.4 |  | 2.7 ± 0.1 |  | 2.6 ± 0.1 |  | 6.8 ± 0.3 |  |
|  | cluster_243 | 9.12 | 1 | *syn*-*syn* | 0.93 | 4.0 ± 0.3 |  | 9.4 ± 0.4 |  | 8.0 ± 0.4 |  | 2.6 ± 0.1 |  | 2.6 ± 0.1 |  | 7.4 ± 0.4 |  |
|  | cluster_230 | 9.49 | 1.7 | *syn*-*syn* | 0.99 | 3.9 ± 0.3 |  | 9.7 ± 0.4 |  | 7.9 ± 0.4 |  | 2.6 ± 0.1 |  | 2.6 ± 0.1 |  | 7.0 ± 0.3 |  |
|  | cluster_232 | 9.66 | 1.5 | *syn*-*syn* | 0.94 | 4.0 ± 0.3 |  | 8.9 ± 0.4 |  | 7.4 ± 0.5 |  | 2.6 ± 0.1 |  | 2.6 ± 0.1 |  | 7.0 ± 0.5 |  |

^a^ System name (**Table S5**). ^b^ In kcal/mol. ^c^ Percentage of structures observed in the combined trajectory representing the cluster. ^d^ Chi torsions of the middle mismatches. ^e^ Total number of hydrogen bonds observed in middle base pairs. For residue numbering, see **Figure 2**. Red highlighted sections display MD data not agreeing with NMR results.

**Table S26**. Comparison of structurally significant NOEs to predicted distances for each cluster in 7A-G (see also **Tables S9** and **S22**). Values next to ± display the standard deviations.

| **Case^a^** | **Cluster** | **ΔG**  **(kcal/mol)** | **% Fraction^b^** | **χ_5_-χ_14_^c^** | **Hydrogen**  **Bond^d^** | **7A5@H2-C6@H5** | | **G14@H1′-G14@H8** | | **7A5@H1′-7A5@H8** | |
| --- | --- | --- | --- | --- | --- | --- | --- | --- | --- | --- | --- |
|  |  |  |  |  |  | **Predicted**  **Distance (Å)** | **NOE**  **Intensity** | **Predicted**  **Distance (Å)** | **NOE**  **Intensity** | **Predicted**  **Distance (Å)** | **NOE**  **Intensity** |
| **7A-G** | cluster_170 | 2.10 | 1.2 | *syn*-*anti* | 1.54 | 4.0 ± 0.4 | Weak | 3.8 ± 0.1 | Weak | 2.7 ± 0.1 | Strong |
|  | cluster_169 | 2.63 | 5.0 | *syn*-*anti* | 1.63 | 3.6 ± 0.3 |  | 3.8 ± 0.1 |  | 2.6 ± 0.1 |  |
|  | cluster_168 | 2.88 | 3.8 | *syn*-*anti* | 1.45 | 3.9 ± 0.4 |  | 3.8 ± 0.1 |  | 2.6 ± 0.1 |  |
|  | cluster_172 | 3.22 | 1.6 | *syn*-*anti* | 1.82 | 3.7 ± 0.4 |  | 3.7 ± 0.1 |  | 2.6 ± 0.1 |  |
|  | cluster_2 | 4.13 | 2.2 | *anti*-*anti* | 1.00 | 4.3 ± 0.5 |  | 3.8 ± 0.1 |  | 3.8 ± 0.1 |  |
|  | cluster_0 | 5.06 | 6.0 | *anti*-*anti* | 1.00 | 4.3 ± 0.4 |  | 3.8 ± 0.1 |  | 3.8 ± 0.1 |  |
|  | cluster_9 | 6.66 | 1.1 | *anti*-*anti* | 1.00 | 4.3 ± 0.5 |  | 3.8 ± 0.1 |  | 3.8 ± 0.1 |  |
|  | cluster_67 | 7.02 | 1.3 | *anti*-*syn* | 0.00 | 5.3 ± 0.4 |  | 2.6 ± 0.1 |  | 3.7 ± 0.1 |  |
|  | cluster_79 | 8.17 | 1.4 | *anti*-*syn* | 0.00 | 5.2 ± 0.4 |  | 2.5 ± 0.1 |  | 3.7 ± 0.1 |  |

^a^ System name (**Table S5**). ^b^ Percentage of structures observed in the combined trajectory representing the cluster. ^c^ Chi torsions of the middle mismatches. ^d^ Total number of hydrogen bonds observed in middle base pairs. For residue numbering, see **Figure 2**. Red highlighted sections display MD data not agreeing with NMR results.

**Table S27**. Comparison of structurally significant NOEs to predicted distances for each cluster in 7A^L^-G (see also **Tables S9** and **S23**). Values next to ± display the standard deviations.

| **Case^a^** | **Cluster** | **ΔG**  **(kcal/mol)** | **% Fraction^b^** | **χ_5_-χ_14_^c^** | **Hydrogen**  **Bond^d^** | **7A^L^5@H2-C6@H5** | | **G14@H1′-G14@H8** | | **7A^L^5@H1′-7A^L^5@H8** | | **7A^L^5@H2-G14@H1** | |
| --- | --- | --- | --- | --- | --- | --- | --- | --- | --- | --- | --- | --- | --- |
|  |  |  |  |  |  | **Predicted**  **Distance (Å)** | **NOE**  **Intensity** | **Predicted**  **Distance (Å)** | **NOE**  **Intensity** | **Predicted**  **Distance (Å)** | **NOE**  **Intensity** | **Predicted**  **Distance (Å)** | **NOE**  **Intensity** |
| **7A^L^-G** | cluster_122 | 1.22 | 7.9 | *syn*-*anti* | 1.75 | 3.8 ± 0.3 | Weak | 3.8 ± 0.1 | Weak | 2.6 ± 0.1 | Strong | 3.1 ± 0.3 | Medium |
|  | cluster_124 | 1.35 | 4.4 | *syn*-*anti* | 1.74 | 3.7 ± 0.3 |  | 3.8 ± 0.1 |  | 2.6 ± 0.1 |  | 3.1 ± 0.3 |  |
|  | cluster_125 | 2.08 | 2.9 | *syn*-*anti* | 1.53 | 3.9 ± 0.3 |  | 3.8 ± 0.1 |  | 2.6 ± 0.1 |  | 2.9 ± 0.3 |  |
|  | cluster_1 | 2.27 | 4.4 | *anti*-*anti* | 1.00 | 3.9 ± 0.4 |  | 3.8 ± 0.1 |  | 3.8 ± 0.1 |  | 2.1 ± 0.2 |  |
|  | cluster_123 | 2.51 | 1.6 | *syn*-*anti* | 1.77 | 3.9 ± 0.3 |  | 3.8 ± 0.1 |  | 2.6 ± 0.1 |  | 3.6 ± 0.4 |  |
|  | cluster_0 | 2.54 | 4.3 | *anti*-*anti* | 1.00 | 4.0 ± 0.4 |  | 3.8 ± 0.1 |  | 3.8 ± 0.1 |  | 2.1 ± 0.2 |  |
|  | cluster_5 | 2.94 | 2.5 | *anti*-*anti* | 1.00 | 3.9 ± 0.4 |  | 3.8 ± 0.1 |  | 3.8 ± 0.1 |  | 2.2 ± 0.3 |  |
|  | cluster_2 | 3.00 | 1.3 | *anti*-*anti* | 1.00 | 4.1 ± 0.4 |  | 3.8 ± 0.1 |  | 3.8 ± 0.1 |  | 2.2 ± 0.2 |  |
|  | cluster_3 | 4.01 | 1.7 | *anti*-*anti* | 1.00 | 3.7 ± 0.4 |  | 3.8 ± 0.1 |  | 3.8 ± 0.1 |  | 2.4 ± 0.2 |  |
|  | cluster_4 | 4.85 | 1 | *anti*-*anti* | 1.00 | 5.5 ± 0.4 |  | 3.8 ± 0.1 |  | 3.7 ± 0.1 |  | 5.9 ± 0.3 |  |
|  | cluster_58 | 5.74 | 1.1 | *anti*-*syn* | 0.00 | 4.9 ± 0.3 |  | 2.6 ± 0.1 |  | 3.8 ± 0.1 |  | 6.9 ± 0.3 |  |
|  | cluster_59 | 6.36 | 1.3 | *anti*-*syn* | 0.00 | 5.1 ± 0.4 |  | 2.6 ± 0.1 |  | 3.8 ± 0.1 |  | 6.9 ± 0.4 |  |
|  | cluster_48 | 7.25 | 3 | *anti*-*syn* | 0.00 | 4.8 ± 0.4 |  | 2.5 ± 0.1 |  | 3.8 ± 0.1 |  | 7.4 ± 0.4 |  |
|  | cluster_52 | 7.94 | 1.1 | *anti*-*syn* | 0.00 | 5.3 ± 0.3 |  | 2.5 ± 0.1 |  | 3.8 ± 0.1 |  | 7.5 ± 0.4 |  |
|  | cluster_51 | 8.20 | 2.1 | *anti*-*syn* | 0.00 | 5.1 ± 0.3 |  | 2.5 ± 0.1 |  | 3.8 ± 0.1 |  | 7.3 ± 0.4 |  |

^a^ System name (**Table S5**). ^b^ Percentage of structures observed in the combined trajectory representing the cluster. ^c^ Chi torsions of the middle mismatches. ^d^ Total number of hydrogen bonds observed in middle base pairs. For residue numbering, see **Figure 2**. Red highlighted sections display MD data not agreeing with NMR results.

**Table S28.** Extended UV-melting experimental data performed on A-A, 7A-A, AL-A, 7AL-A, A-G, 7A-G, AL-G, and 7AL-G.^a^

| **Duplexes**  **(5′-3′)** | **Short**  **notation** | **Average of curve fits** | | | |  | **T_M_^-1^ vs log C_T_ plots** | | | | |
| --- | --- | --- | --- | --- | --- | --- | --- | --- | --- | --- | --- |
|  |  | **-ΔH˚**  **(kcal/mol)** | **-ΔS˚**  **(eu)** | **-ΔG˚_37_**  **(kcal/mol)** | **T_M_^b^**  **(˚C)** |  | **-ΔH˚**  **(kcal/mol)** | **-ΔS˚**  **(eu)** | **-ΔG˚_37_^c^**  **(kcal/mol)** | **-ΔG˚_37,average_^d^**  **(kcal/mol)** | **T_M_^b^**  **(˚C)** |
| 5′ UCAG**A**CAGU  3′ AGUC**A**GUCA | A-A | 70.7 ± 5.8  61.8 ± 5.4 | 202.6 ± 18.6  175.5 ± 17.6 | 7.84 ± 0.16  7.34 ± 0.11 | 42.9  41.1 |  | 68.4 ± 10.2  67.5 ± 2.7 | 195.4 ± 32.5  194.1 ± 8.6 | 7.80 ± 0.33 (3%)  7.34 ± 0.03 (3%) | 7.57 | 42.8  40.8 |
| 5′ UCAG**7A**CAGU  3′AGUC **A**GUCA | 7A-A | 72.3 ± 4.1  73.3 ± 2.7  67.3 ± 2.7 | 205.9 ± 12.7  208.4 ± 8.7  189.5 ± 8.8 | 8.49 ± 0.19  8.69 ± 0.07  8.57 ± 0.11 | 45.6  46.4  46.7 |  | 79.1 ± 7.1  72.9 ± 4.5  67.2 ± 3.5 | 227.9 ± 22.6  207.0 ± 14.1  189.5 ± 11.2 | 8.53 ± 0.14 (1%)  8.67 ± 0.10 (1%)  8.55 ± 0.07 (0%) | 8.58 | 45.0  46.4  46.6 |
| 5′ UCAG**A^L^**CAGU  3′ AGUC**A** GUCA | A^L^-A | 64.2 ± 3.8  64.1 ± 4.6 | 180.5 ± 11.7  179.6 ± 14.6 | 8.23 ± 0.18  8.40 ± 0.10 | 45.4  46.3 |  | 58.5 ± 4.1  61.8 ± 4.3 | 162.5 ± 13.0  172.5 ±13.7 | 8.13 ± 0.10 (1%)  8.34 ± 0.10 (1%) | 8.24 | 45.7  46.5 |
| 5′ UCAG**7A^L^**CAGU  3′ AGUC **A** GUCA | 7A^L^-A | 73.2 ± 2.0  73.7 ± 4.9 | 204.6 ± 5.9  204.4 ± 15.0 | 9.80 ± 0.17  10.28 ± 0.25 | 51.5  53.6 |  | 67.6 ± 2.1  69.5 ± 2.0 | 187.0 ± 6.7  191.5 ± 6.1 | 9.60 ± 0.07 (2%)  10.06 ± 0.08 (2%) | 9.83 | 51.8  53.6 |
| 5′ UCAG**A**CAGU  3′ AGUC**G**GUCA | A-G | 64.9 ± 2.2  69.7 ± 4.5  67.5 ± 1.7  77.4 ± 2.8 | 197.0 ± 6.8  196.5 ± 14.0  189.7 ± 5.3  219.7 ± 11.1 | 8.73 ± 0.13  8.77 ± 0.20  8.62 ± 0.10  9.24 ± 0.20 | 47.1  47.3  46.9  48.3 |  | 64.5 ± 3.3  71.0 ± 3.5  73.2 ± 3.2  71.2 ± 2.8 | 180.3 ± 10.3  200.7 ± 11.0  207.7 ± 10.2  200.6 ± 8.7 | 8.61 ± 0.08 (2%)  8.77 ± 0.10 (0%)  8.74 ± 0.08 (1%)  9.05 ± 0.08 (3%) | 8.79 | 47.3  47.1  46.9  48.4 |
| 5′ UCAG**7A**CAGU  3′AGUC **G** GUCA | 7A-G | 59.4 ± 1.5  61.2 ± 1.4  64.4 ± 2.8 | 166.8 ± 4.0  172.6 ± 4.2  182.4 ± 9.2 | 7.62 ± 0.06  7.64 ± 0.08  7.85 ± 0.12 | 42.8  42.7  43.5 |  | 60.0 ± 4.3  64.4 ± 3.3  73.8 ± 3.2 | 168.8 ± 13.9  183.0 ± 10.6  209.0 ± 10.2 | 7.62 ± 0.08 (1%)  7.66 ± 0.05 (1%)  7.92 ± 0.04 (2%) | 7.73 | 42.8  42.6  43.0 |
| 5′ UCAG**A^L^**CAGU  3′ AGUC **G** GUCA | A^L^-G | 69.1 ± 2.8  74.9 ± 8.6 | 192.1 ± 9.0  209.2 ± 26.5 | 9.49 ± 0.02  10.02 ± 0.37 | 50.9  52.1 |  | 61.1 ± 1.0  68.8 ± 2.8 | 167.0 ± 3.2  190.6 ± 8.8 | 9.32 ± 0.02 (2%)  9.70 ± 0.12 (2%) | 9.51 | 51.8  52.1 |
| 5′ UCAG**7A^L^**CAGU  3′AGUC **G** GUCA | 7A^L^-G | 74.8 ± 2.4  82.2 ± 4.0 | 208.9 ± 7.7  229.7 ± 11.7 | 9.98 ± 0.08  11.00 ± 0.33 | 52.0  54.8 |  | 71.8 ± 2.6  70.9 ± 2.9 | 199.8 ± 8.3  195.0 ± 8.8 | 9.89 ± 0.08 (3%)  10.39 ± 0.15 (2%) | 10.14 | 52.2  54.9 |

^a^ Solution: 1 M sodium chloride, 20 mM sodium cacodylate, 0.5 mM Na_2_EDTA, pH 7. ^b^ Calculated for 10^-4^ M oligomer concentration (7A = N7 regioisomer of A; 7A^L^ = N7 regioisomer of LNA-A; A^L^ = LNA-A). ^c^ Measured ΔG˚_37_ values. Values in parantheses represent |ΔG˚_37_ - ΔG˚_37,Average_|/ΔG˚_37_, indicating percentage difference from ΔG˚_37,Average_. ^d^ Average values calculated from measured ΔG˚_37_ values. Data shown in green corresponds to newly synthesized duplexes, while data shown in red represents additional UV-melting measurements performed on the original sample.

**Figure S1.** ^1^H NMR spectrum of 7-β-D-ribofuranosyladenine in DMSO-d_6_.

**Figure S2.** ^13^C NMR spectrum of 7-β-D-ribofuranosyladenine in DMSO-d_6_.

**Figure S3.** ^1^H-^1^H COSY spectrum of 7-β-D-ribofuranosyladenine in DMSO-d_6_.

**Figure S4.** ^1^H-^13^C gHSQC spectrum of 7-β-D-ribofuranosyladenine in DMSO-d_6_.

**Figure S5.** ^1^H-^13^C gHMBC spectrum of 7-β-D-ribofuranosyl adeninę in DMSO-d_6_. The C5-H1′ cross-peak diagnostic for the N7 substitution is marked with a red circle.

**Figure S6.** ^1^H NMR spectrum of 2′-O,4′-C-Methylene-7-β-D-ribofuranosyladenine in DMSO-d_6_.

**Figure S7.** ^13^C NMR spectrum of 2′-O,4′-C-Methylene-7-β-D-ribofuranosyladenine in DMSO-d_6_.

**Figure S8.** ^1^H-^13^C gHSQC spectrum of 2′-O,4′-C-Methylene-7-β-D-ribofuranosyladenine in DMSO-d_6_.

**Figure S9.** ^1^H-^13^C gHMBC spectrum of 2′-O,4′-C-Methylene-7-β-D-ribofuranosyladenine in DMSO-d_6_. The C5-H1′ cross-peak diagnostic for the N7 substitution is marked with a red circle.

**Figure S10.** ^1^H NMR spectrum of 9-β-D-ribofuranosyladenine in DMSO-d_6_.

**Figure S11.** ^13^C NMR spectrum of 9-β-D-ribofuranosyladenine in DMSO-d_6_.

**Figure S12.** ^1^H-^13^C gHSQC spectrum of 9-β-D-ribofuranosyladenine in DMSO-d_6_.

**Figure S13.** ^1^H-^13^C gHMBC spectrum of 9-β-D-ribofuranosyladenine in DMSO-d_6_. The C4-H1′ cross-peak diagnostic for the N9 substitution is marked with a red circle.

**Figure S14.** Fragments of ^1^H-^13^C gHMBC spectra of 9-β-D-ribofuranosyladenine, 7-β-D-ribofuranosyladenine and 2′-O,4′-C-Methylene-7-β-D-ribofuranosyladenine (left to right) showing long range ^1^H-^13^C correlations crucial for determination of D-ribofuranosyl moiety position with respect to adenine base (C4-H1′ and C5-H1′ cross-peaks diagnostic respectively for N9 and N7 substitutions are marked with red circles).

**Figure S15.** Imino regions in 1D ^1^H NMR spectra recorded at four different temperatures for the duplex containing 7A^L^:G mismatch. The imino proton belonging to G_14_ in the mismatch is highlighted in red. For residue numbering see **Figure 2**.

| 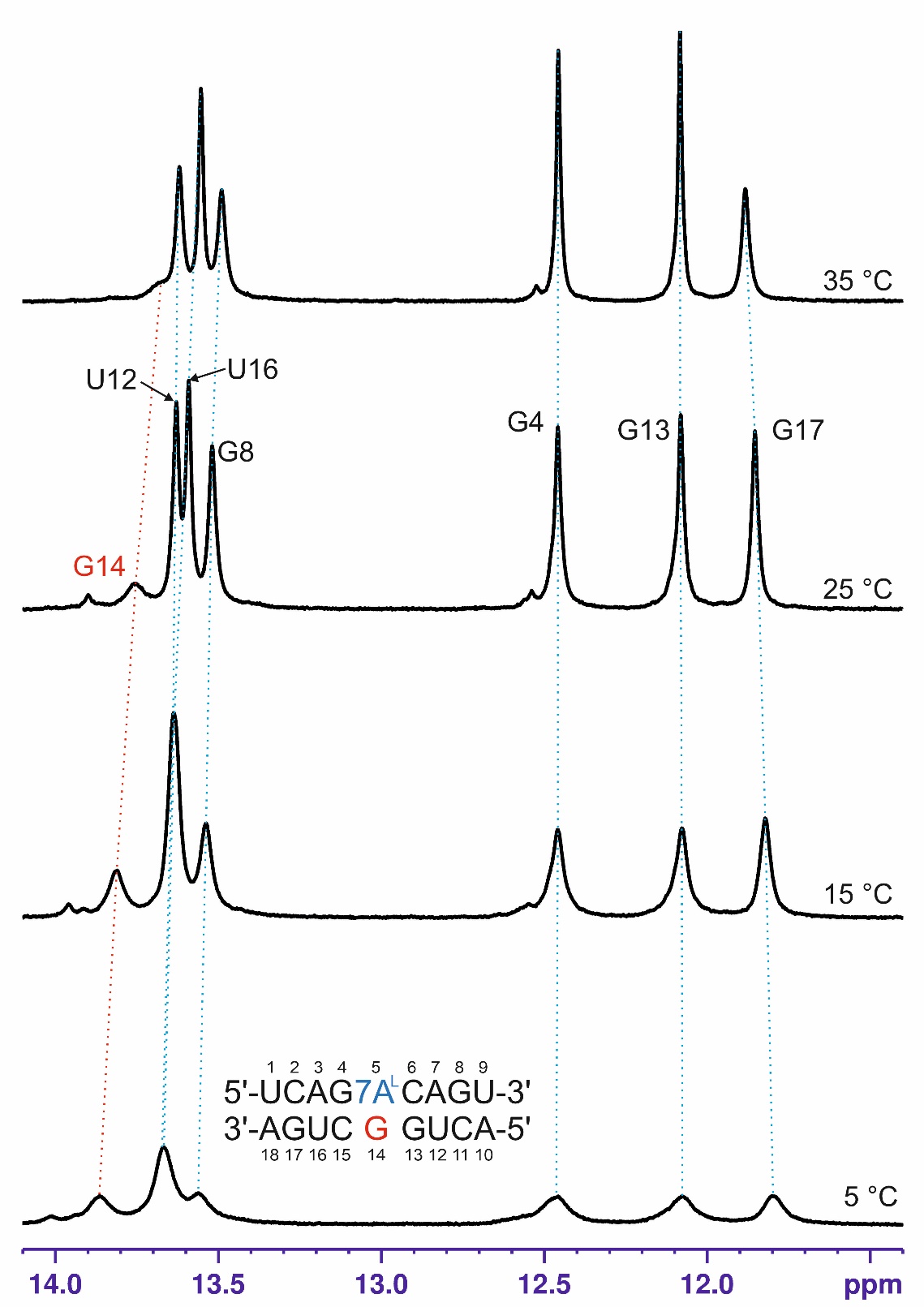 |
| --- |

**Figure S16.** The imino-imino and imino-amino/aromatic regions of the 2D ^1^H-^1^H NOESY spectra recorded for duplexes containing 1×1 7A:G and 1×1 7A^L^:G mismatches. Significant NOEs discussed in the text are marked in magenta. For residue numbering see **Figure 2**.

| **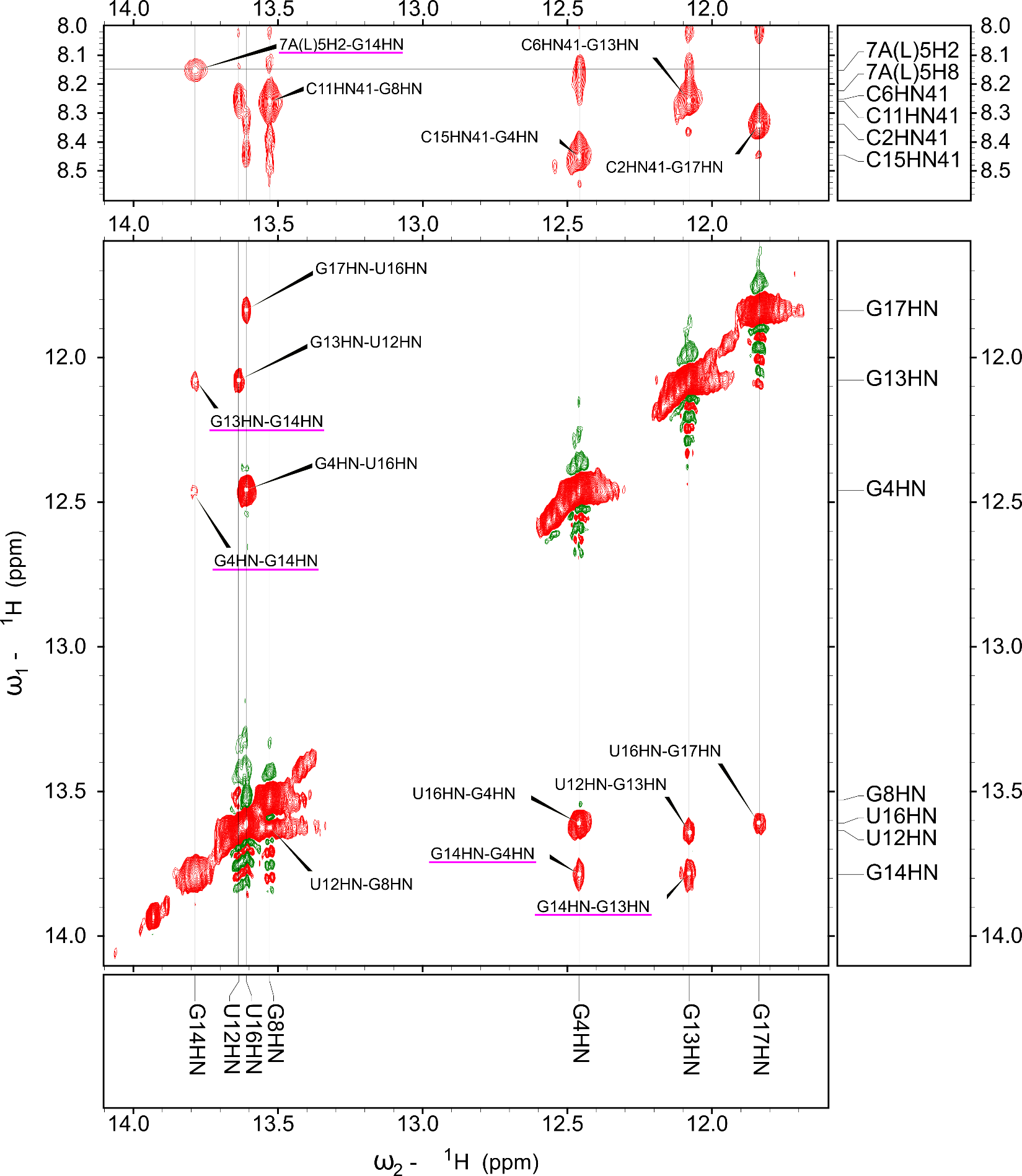** |
| --- |

**Supplemental References.**

1. Framski, G., Gdaniec, Z., Gdaniec, M. and Boryski, J. (2006) A reinvestigated mechanism of ribosylation of adenine under silylating conditions. *Tetrahedron*, **62**, 10123-10129.

2. Beaucage, S.L. and Caruthers, M.H. (1981) Deoxynucleoside phosphoramidites - A new class of key intermediates for deoxypolynucleotide synthesis. *Tetrahedron Lett.*, **22**, 1859-1862.

3. McBride, L.J. and Caruthers, M.H. (1983) An investigation of several deoxynucleoside phosphoramidites useful for synthesizing deoxyoligonucleotides. *Tetrahedron Lett.*, **24**, 245-248.

4. Akhrem, A.A., Mikhailopulo, I.A. and Abramov, A.F. (1979) 13C nuclear magnetic resonance spectroscopy of selected adenine nucleosides: Structural correlation and conformation about the glycosidic bond. *Org. Magn. Reson.*, **12**, 247-253.

5. SDBSWeb. <https://sdbs.db.aist.go.jp>.

6. Altona, C. and Sundaralingam, M. (1972) Conformational analysis of the sugar ring in nucleosides and nucleotides. A new description using the concept of pseudorotation. *J. Am. Chem. Soc.*, **94**, 8205-8212.

7. De Leeuw, F.A.A.M. and Altona, C. (1983) Computer-assisted pseudorotation analysis of five-membered rings by means of proton spin–spin coupling constants: Program PSEUROT. *J. Comput. Chem.*, **4**, 428-437.

8. Ippel, J.H., Wijmenga, S.S., de Jong, R., Heus, H.A., Hilbers, C.W., de Vroom, E., van der Marel, G.A. and van Boom, J.H. (1996) Heteronuclear Scalar Couplings in the Bases and Sugar Rings of Nucleic Acids: Their Determination and Application in Assignment and Conformational Analysis. *Magn. Reson. Chem.*, **34**, S156-S176.
